# Targeting the PDK1/c‐Myc/SOX10 Signaling in Oligodendrocytes Alleviates Neuropathic Pain

**DOI:** 10.1002/advs.202516426

**Published:** 2026-04-16

**Authors:** Pingping Qiao, Lifang Guo, Guochao Yang, Chaoli Huang, He Wang, Jian‐Jun Yang, Guiquan Chen, Yimin Hu

**Affiliations:** ^1^ State Key Laboratory of Pharmaceutical Biotechnology Department of Neurosurgery Nanjing Drum Tower Hospital, Affiliated Hospital of Medical School Jiangsu Key Laboratory of Molecular Medicine Model Animal Research Center Medical School Nanjing University Nanjing China; ^2^ Co‐innovation Center of Neuroregeneration Nantong University Nantong China; ^3^ Department of Laser Surgery Hospital of Dermatology Chinese Academy of Medical Sciences and Peking Union Medical College Nanjing China; ^4^ Department of Anesthesiology and Perioperative Medicine The First Affiliated Hospital of Nanjing Medical University Nanjing China; ^5^ Department of Anesthesiology Pain and Perioperative Medicine The First Affiliated Hospital of Zhengzhou University Zhengzhou China; ^6^ Department of Anesthesiology Hospital of Dermatology Chinese Academy of Medical Sciences and Peking Union Medical College Nanjing China

**Keywords:** c‐Myc, myelin, neuropathic pain, oligodendrocyte, PDK1, SOX10

## Abstract

Neuropathic pain (NPP) is a critical clinical challenge with limited therapeutic options. While neuronal mechanisms have been extensively studied, the contribution of oligodendrocyte (OL) homeostasis to NPP pathogenesis is poorly understood. Here, we show that chronic constriction injury (CCI) causes demyelination and downregulation of 3‐phosphoinositide‐dependent kinase 1 (PDK1) in the central nervous system (CNS) in mice. Functional analysis of inducible OL lineage‐specific *Pdk1* conditional knockout (*Pdk1* cKO) mice reveals mechanical allodynia and thermal hyperalgesia, a pattern of sensory changes that closely resembles NPP in CCI mice. RNA sequencing (RNA‐seq), morphological, and molecular analyses demonstrate that PDK1 deficiency impairs myelination via the c‐Myc/SOX10 axis. Notably, pharmacological enhancement of remyelination or AAV‐mediated knockdown of c‐Myc restores nodal integrity and alleviates NPP in *Pdk1* cKO mice. Our findings establish PDK1‐mediated OL homeostasis as a critical determinant of NPP pathogenesis and identify c‐Myc modulation as a novel therapeutic strategy for NPP.

## Graphical abstract

This work reveals that oligodendrocyte homeostasis, mediated by PDK1, is a critical determinant of neuropathic pain (NPP) pathogenesis. Disruption of PDK1 in oligodendrocytes impairs SOX10‐dependent myelination programs through c‐Myc accumulation, leading to disrupted myelination and the pathophysiology of NPP. Targeting the PDK1/c‐Myc/SOX10 axis restores oligodendrocyte differentiation, enhances remyelination, and alleviates NPP, highlighting a promising therapeutic strategy.

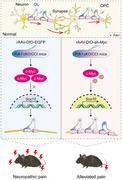

## 1 Introduction

NPP is defined as pain caused by a lesion or disease of the somatosensory nervous system [1], constituting a debilitating chronic condition that affects 7–10% of the global population [2]. Characterized by long‐term chronic pain within affected innervation territories or corresponding body parts, NPP presents a formidable clinical challenge due to its refractory nature. Accumulating evidence underscores the pathophysiological complexity of NPP, wherein hyperexcitability of nociceptive neurons and chronic activation of non‐neuronal cells (particularly microglia and astrocytes) drive central sensitization and maladaptive plasticity. However, beyond the neuron‐glia axis, the integrity of myelin, a critical determinant of efficient saltatory conduction, remains understudied in NPP pathogenesis.

OLs, the myelinating cells of the CNS, arise from oligodendrocyte precursor cells (OPCs) and orchestrate axonal ensheathment, metabolic support, and neurotransmitter regulation. Lifelong myelin remodeling by OLs ensures the maintenance of Nodes of Ranvier and nerve impulse rapid saltatory conduction [3]. Alterations in node length are closely associated with changes in nerve conduction velocity, and elongation of nodes is generally linked to reduced conduction speed [4]. Notably, NPP‐associated insults (e.g., trauma, neuroinflammation, or metabolic dysfunction) frequently precipitate OL loss or myelin injury, leading to axonal vulnerability, conduction deficits, and aberrant nociceptive signaling [5]. Despite the pivotal role of OL differentiation and remyelination in preserving axonal homeostasis, their contribution to NPP progression remains inadequately explored.

PDK1 (also known as PDPK1), a master regulator of the PI3K/Akt signaling pathway, mediates diverse CNS developmental processes, including neurogenesis, cell survival, and cytoskeletal dynamics [6, 7, 8]. Clinical evidence indicates that a heterozygous missense mutation of *PDK1* (c.1139G > A; p.G380D) is associated with developmental delay and early‐onset refractory epilepsy in patients [9]. While our recent study reveals a critical role of PDK1 in the survival of neural progenitor cells (NPCs) [10], it has been shown that PDK1 is also required for the proliferation of NPCs in the cortex and cerebellum [8, 11]. PDK1 may also play an important role in cognitive functions, since disruption of the PDK1 substrate‐docking site has been found to cause learning impairment [12]. Despite these established roles in disease pathogenesis, the function of oligodendrocytic PDK1 in NPP remains unresolved.

To address the above question, we first established a CCI‐induced mouse model of NPP [13]. We observed deficient myelination in the ipsilateral spinal cords and downregulation of PDK1 in OL lineage cells of CCI mice. To explore the underlying mechanisms, we generated inducible *Pdk1* cKO mice by breeding floxed *Pdk1* mice with *NG2‐CreERT2* animals [14]. We found that the *Pdk1* cKO mouse model exhibits remarkable NPP‐related phenotypes. Mechanistically, we identified a novel OL‐autonomous regulatory axis for NPP: while PDK1 governs proteasomal degradation of c‐Myc, the latter liberates *Sox10* to execute transcriptional programs for myelination. Disruption of the PDK1/c‐Myc/SOX10 axis triggers tripartite pathology: demyelination, axonal dystrophy, and functional deficits. These factors collectively exacerbate central sensitization and mechanical allodynia. Together, this study establishes the oligodendrocytic PDK1/c‐Myc /SOX10 axis as a promising therapeutic strategy to restore myelin homeostasis and alleviate NPP.

## 2 Results

### 2.1 Demyelination in the Ipsilateral Spinal Cords Correlates with Hyperalgesia in NPP

**FIGURE 1:**
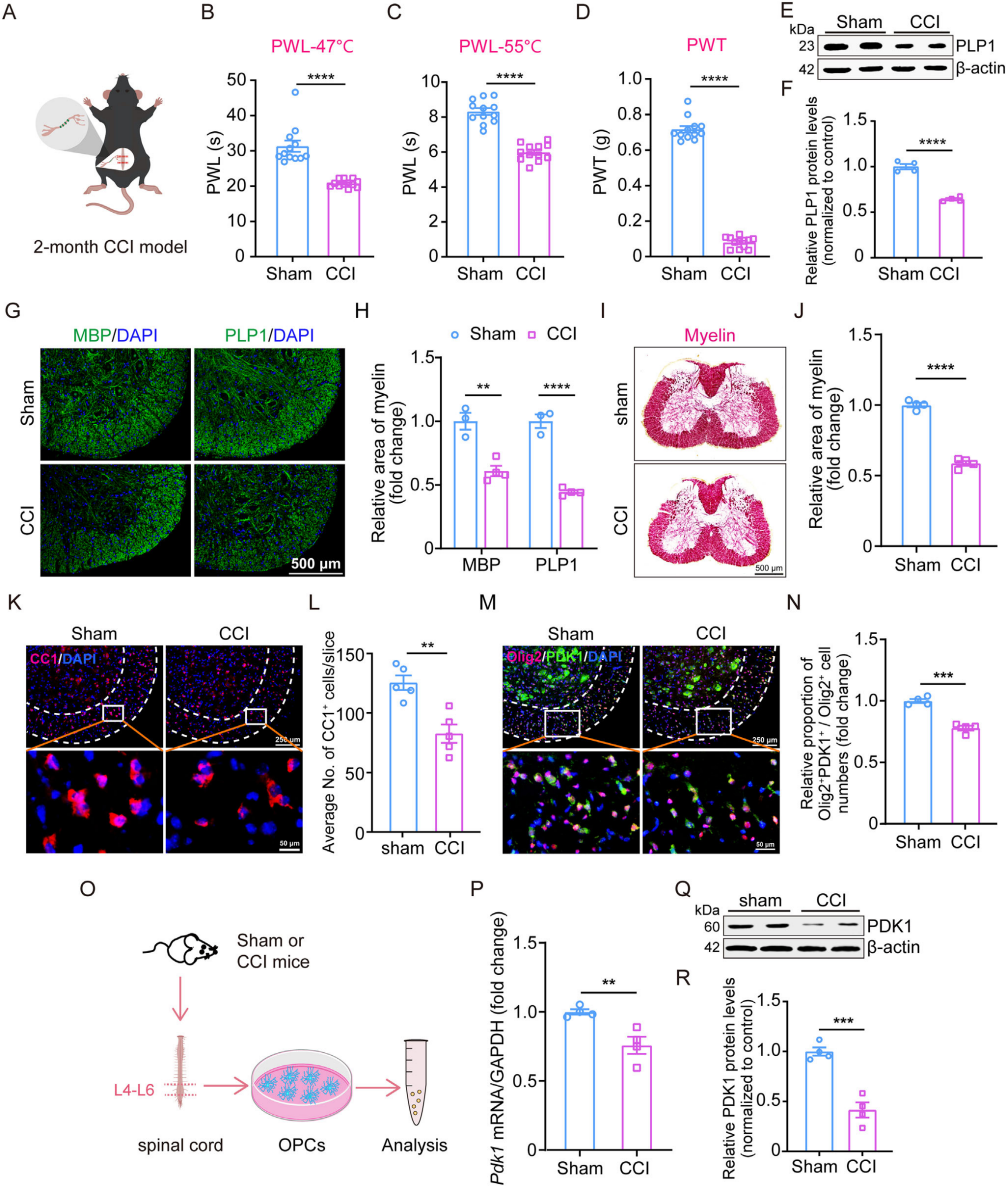
CCI mice developed hyperalgesia accompanied by ipsilateral spinal cord myelin defects and an OL‐specific reduction in PDK1. A. Schematic diagram of the CCI‐induced mouse NPP model. B‐D. PWL (B, C) and PWT (D) were assessed at day 14 after the CCI surgery. Both the PWL and the PWT tests revealed significant mechanical and thermal hyperalgesia in CCI mice compared to sham controls (unpaired t test; mean ± SEM; n = 12 mice per group). E. WB images of PDK1 and β‐actin. F. Quantification of PLP1 protein levels normalized to β‐actin. The WB analysis showed a significant reduction in the expression levels of myelin‐associated proteins, PLP1, in the ipsilateral spinal cords following CCI injury (unpaired t test; mean ± SEM; n = 4 mice per group). G. IHC staining of MBP (green, left) or PLP1 (green, right). Scale bar = 500 µm. H. Quantification of MBP^+^ and PLP1^+^ myelinated area in the ventral and lateral white matter. IHC staining showed a marked decrease in myelinated fibers in the ipsilateral spinal cords after CCI injury (unpaired t test; mean ± SEM; n ≥ 4 mice per group). I. Representative images of TrueGold myelin staining in sham and CCI mice. Scale bar = 500 µm. J. Quantification of myelinated fiber area in the ventral and lateral white matter. TrueGold staining revealed a significant reduction in myelin density in the spinal cords of CCI mice compared with sham controls (unpaired t test; mean ± SEM; n = 4 mice per group). K. Representative IHC images of CC1 (red) in the ipsilateral spinal cords. The zone between the two white dashed lines demarcates the territory of the spinal cord's white matter. White Box: The highlighted white matter area selected for magnification. Scale bar = 250 and 50 µm. L. Quantification of averaged CC1^+^. IHC staining of CC1 showed a significantly reduced number of OLs in the ipsilateral spinal cords compared to sham controls (unpaired t test; mean ± SEM; n ≥ 4 mice per group). M. Representative IHC images of Olig2 (red) and PDK1 (green) in the ipsilateral spinal cords. Scale bar = 250 and 50 µm. N. The percentage of Olig2^+^PDK1^+^/ Olig2^+^ cell numbers was significantly downregulated after CCI injury (unpaired t test; mean ± SEM; n = 5 mice per group). O. Schematic illustration of primary OPC culture. P. qRT‐PCR analysis of *Pdk1* was significantly decreased in primary OPC after CCI injury (unpaired t test; mean ± SEM; n = 4 mice per group). Q. WB images of PDK1 and β‐actin. R. Quantification of PDK1 protein levels normalized to β‐actin. WB analysis showed that the protein levels of PDK1 were remarkably downregulated in the primary OPCs after CCI injury compared to controls (unpaired t test; mean ± SEM; n = 5 mice per group). *p< 0.05; **p< 0.01; ***p< 0.001; ****p< 0.0001; ns, no significant difference.

To investigate the mechanisms underlying NPP, we established a CCI mouse model [13], a well‐characterized preclinical paradigm for studying neuropathy (Figure 1A). Behavioral assessments, including thermal and mechanical nociceptive tests, demonstrated pronounced hyperalgesia in CCI mice on day 14 after CCI surgery (Figure 1B–D). Specifically, thermal allodynia, measured by paw withdrawal latency (PWL), and mechanical hypersensitivity, evaluated via the 50% paw withdrawal threshold (PWT), were significantly exacerbated in CCI mice compared to sham controls (Figure 1B–D). Independent analyses of male and female mice yielded consistent outcomes (Figure S1). Given the critical role of myelination in nociceptive processing, we next examined myelination status in the ipsilateral spinal cords. Western blot (WB) analysis revealed a marked reduction in proteolipid protein 1 (PLP1), a key myelin structural component, in CCI mice relative to sham‐operated controls (Figure 1E,F). Furthermore, quantitative RT‐PCR (qRT‐PCR) confirmed substantial downregulation of myelin‐related transcripts, including *Mbp*, *Plp1*, *Mag*, and *Mog*, in the ipsilateral spinal cords of CCI mice (Figure S2A). Immunohistochemical (IHC) validation demonstrated a pronounced loss of myelin basic protein‐positive (MBP^+^) and PLP1^+^ fibers in CCI mice, corroborating the biochemical data (Figure 1G,H). We also performed the TrueGold myelin staining [15] in CCI and sham‐operated mice. In line with MBP and PLP1 immunostaining results, quantitative analysis confirmed marked myelin loss in the spinal white matter of CCI mice relative to sham controls (Figure 1I,J). These findings indicate that NPP‐associated hyperalgesia is closely associated with myelin fiber deficits in the mouse spinal cords, suggesting that targeting myelin integrity may serve as a potential regulator of NPP sensitization.

### 2.2 Dysregulation of PDK1 in OL Lineage Cells is Linked to NPP Pathogenesis

Given the observed demyelination in CCI mice, we next investigated the underlying molecular mechanisms. IHC analysis revealed a significant depletion of OL lineage cells, e.g., CC1^+^ (mature OLs), PDGFRα^+^ (OPCs), and Olig2^+^ (total OL lineage cells), in the white matter of CCI ipsilateral spinal cords vs. sham controls (Figure 1K,L; Figure S2B–E), suggesting disrupted OL lineage homeostasis post‐injury. In addition, qRT‐PCR revealed a robust upregulation of mRNA levels of multiple pro‐inflammatory cytokines and chemokines, including *Tnf*, *Il1b*, *Il13*, *Il6*, and *Ifng*, in the ipsilateral spinal cords following CCI (Figure S2F), consistent with previous studies reporting spinal neuroinflammation and glial activation in NPP models [16, 17]. Considering that PDK1 serves as a pivotal kinase within the PI3K/Akt pathway, regulating OL survival, differentiation, and myelin maintenance [18], we next detected its expression in the spinal cords after CCI injury. Notably, co‐immunostaining experiments demonstrated pronounced downregulation of PDK1 specifically in Olig2^+^ OL lineage cells of CCI mice (Figure 1M,N), while neuronal (NeuN*^+^*) and astrocytic (GFAP^+^) PDK1 levels were unaltered (Figure S2G,H). Concomitantly, we observed reduced phosphorylation of Akt at Thr308 (p‐Akt^Thr308^) in OL lineage cells (Figure S2I,J), confirming pathway suppression in this compartment. Notably, this reduction in PDK1 activity was evident at 14 days post‐CCI but not at early time points (Figure S2K,L). To further validate this cell‐type‐specific deficit, primary OPCs isolated from CCI spinal cords exhibited decreased *Pdk1* mRNA and protein expression (Figure 1O–R), corroborating in vivo findings. Collectively, OL‐specific PDK1 dysregulation represents a distinct mechanism contributing to a deficit in remyelination under NPP conditions, independent of neuronal signaling.

### 2.3 Loss of PDK1 in OL Lineage Cells Drives Pain Hypersensitivity and Spinal Cord Hypomyelination

**FIGURE 2:**
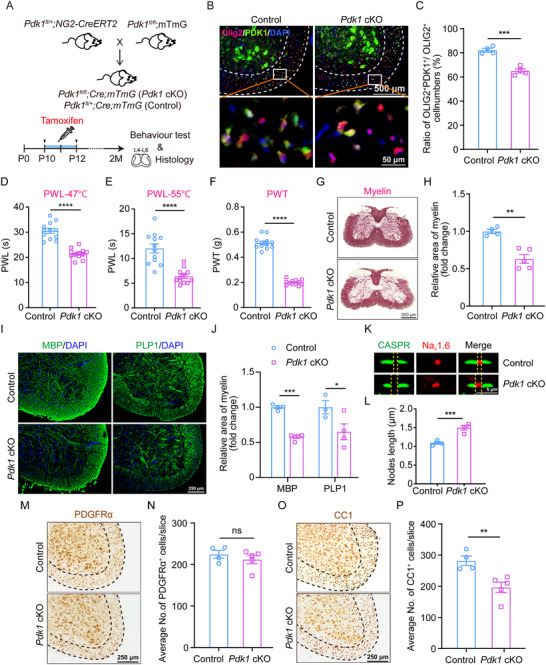
OL lineage‐specific *Pdk1* knockout caused persistent pain hypersensitivity, spinal cord demyelination, and reduced OL numbers. A. Strategy of constructing *Pdk1* cKO mice. B. Double staining of Olig2 (red) and PDK1 (green) in the spinal cords. Scale bar = 500 and 50 µm. C. Quantification of the ratio of Olig2^+^PDK1^+^/ Olig2^+^ cells in the spinal cords of the two groups of mice. The ratio of Olig2^+^PDK1^+^/ Olig2^+^ cells was significantly decreased in the *Pdk1* cKO spinal cords (unpaired t test; mean ± SEM; n = 4 mice per group). D‐F. Mechanical and thermal hypersensitivity were assessed using PWL (D, E) and PWT (F) behavioral tests. The PWL and the PWT tests revealed significant mechanical and thermal hyperalgesia in *Pdk1* cKO mice compared to littermate controls (unpaired t test; mean ± SEM; n = 12 mice per group). G. TrueGold staining of spinal cords. Scale bar = 500 µm. H. Quantification of the relative area of myelin fibers in the ventral and lateral white matter. There was a significant difference in the spinal cords between the control and *Pdk1* cKO mice (unpaired t test; mean ± SEM; n ≥ 4 mice per group). I. Representative IHC images for MBP (left) and PLP1 (right). Scale bar = 250 µm. J. Quantification of MBP^+^ (green) or PLP1^+^ (green) area in the ventral and lateral white matter. The areas labeled by MBP or PLP1 showed myelination patterns consistent with those observed in TrueGold staining (unpaired t test; mean ± SEM; n ≥ 3 mice per group). K. Double IHC staining of CASPR (green) and Na_v_1.6 (red). Scale bar = 5 µm. L. Quantification of the node length. Double IHC staining of CASPR and Na_v_1.6 revealed elongated nodal segments in the spinal cords of *Pdk1* cKO mice (unpaired t test; mean ± SEM; n = 4 mice per group). M, N. Representative IHC images (M) and quantification (N) of averaged PDGFRα^+^ cells in the spinal cords of control and *Pdk1* cKO mice. IHC results showed no significant difference between the two groups (unpaired t test; mean ± SEM; n ≥ 4 mice per group). Scale bar = 250 µm. O. Representative IHC images of CC1 in ventral and lateral white matter. Scale bar = 250 µm. P. Quantification of averaged CC1^+^ cell numbers. IHC staining indicated an obvious decline in CC1^+^ cells in the *Pdk1* cKO spinal cords (unpaired t test; mean ± SEM; n ≥ 4 mice per group). Scale bar = 250 µm. *p< 0.05; **p< 0.01; ***p< 0.001; ****p< 0.0001; ns, no significant difference.

To explore the potential relationship between PDK1 dysfunction in OL lineage cells and NPP, we generated *Pdk1* cKO (*Pdk1^fl/fl^;NG2‐CreERT2;mTmG*) mice by crossing *Pdk1^fl/+^;NG2‐CreERT2* mice with *Pdk1^fl/fl^;mTmG* mice to assess the Cre recombinase expression pattern and to validate OL‐specific *Pdk1* deletion (Figure 2A; Figure S3A). Tamoxifen was administered to mice three times (postnatal days 10–12, P10‐P12). At this developmental stage, OLs in the mouse spinal cords are largely mature (Figure S3B,C), allowing us to investigate how PDK1 regulates myelination without influencing OPC proliferation. We harvested L4‐L6 spinal cord tissues from mice at 2 months. IHC, qRT‐PCR, and WB analyses confirmed efficient *Pdk1* deletion in spinal cord OL lineage cells of *Pdk1* cKO mice (Figure 2B,C; Figure S3D–F). We also generated *Pdk1^fl/fl^;Olig1‐Cre* mice; However, these mice exhibited lethality around P21, precluding functional assessments (Figure S3G). To evaluate the impact of PDK1 deficiency on pain sensitivity, we conducted behavioral assays in 2‐month‐old control and *Pdk1* cKO mice. *Pdk1* cKO mice exhibited significantly lower thresholds in both PWL and PWT tests compared to controls (Figure 2D–F).

To determine whether OL lineage‐specific *Pdk1* deletion causes broad neurological impairment beyond pain hypersensitivity, we performed additional behavioral tests assessing itch response and motor coordination. In the spontaneous scratching test, *Pdk1* cKO mice showed no significant difference in scratching bouts compared with control littermates (Figure S3H), indicating the absence of spontaneous itch‐like behavior. In the open‐field test (OFT), *Pdk1* cKO mice displayed motor activity similar to that of control mice, showing no changes in either total distance traveled or average speeds (Figure S3I,J). Similarly, balance beam walk (BBW) performance was indistinguishable between *Pdk1* cKO and control mice, as shown by similar traversal times to cross the beam and foot‐slip frequencies (Figure S3K,L). Furthermore, in the rotarod test (RT), both genotypes displayed comparable latency to fall across repeated trials (Figure S3M–P), suggesting intact motor coordination and balance. Together, these results demonstrate that *Pdk1* deletion in OL lineage cells does not induce generalized motor or sensory deficits, but rather produces a selective pain hypersensitivity phenotype.

To elucidate the cellular and molecular mechanisms underlying NPP, we initially examined the structural integrity of white matter. Nissl staining revealed that the proportion of white matter was reduced in the *Pdk1* cKO mouse spinal cords compared to controls (Figure S4A,B). Subsequently, we employed multiple approaches to assess myelination status. qRT‐PCR analysis revealed a marked decrease of myelin‐related transcripts, including *Plp1*, *Mbp*, *Mag*, and *Mog* (Figure S4C). Consistent with these findings, WB analysis confirmed a pronounced reduction in PLP1 and MBP protein expression (Figure S4D,E). Further validation using the TrueGold myelin staining kit showed a significant decrease in myelin fiber density in *Pdk1* cKO spinal cords compared to controls (Figure 2G,H). Additionally, IHC demonstrated substantial loss of MBP^+^ and PLP1^+^ myelin fibers in the *Pdk1* cKO spinal cords (Figure 2I,J). Notably, IHC analysis of nodal markers (CASPR and Na_v_1.6) revealed increased nodal length in *Pdk1* cKO mice (Figure 2K,L), indicating compromised nodal integrity secondary to impaired myelination.

To investigate whether OL loss and impaired myelination in the ventrolateral white matter leads to secondary remodeling of dorsal horn circuits, we examined the density of key synaptic markers. IHC analysis showed a significant reduction in Synaptophysin^+^ (pre‐synaptic) and Homer1^+^ (post‐synaptic) puncta on MAP2‐expression neurons in the dorsal horn of *Pdk1* cKO mice (Figure S4F–I). Furthermore, we observed a marked decrease in VGAT^+^ (Vesicular GABA transporter) inhibitory terminals (Figure S4J,K), suggesting an impairment of inhibitory modulation. This synaptic loss, coupled with nodal disruption and Na_v_1.6 redistribution in the white matter, suggests that ventrolateral white matter pathology may trigger a state of synaptic disinhibition to alter synaptic plasticity within the spinal sensory circuitry.

Collectively, these findings demonstrate that OL lineage‐specific *Pdk1* deletion disrupts new myelination, compromises nodal integrity, and induces secondary synaptic remodeling, ultimately contributing to NPP pathogenesis.

### 2.4 Impaired OL Differentiation and Decreased OL Populations in the Spinal Cords of *Pdk1* cKO Mice

We investigated the cellular basis of hypomyelination in *Pdk1* cKO mice through systematic analysis of OL lineage cells in the spinal cords. We carried out a series of IHC staining in OL lineage cells, and quantitative analysis showed that the density of OPCs (PDGFRα^+^) was maintained, whereas mature OLs (CC1^+^) and total OL lineage cells (Olig2^+^) were significantly reduced in the white matter tracts of *Pdk1* cKO mice compared with controls (Figure 2M–P; Figure S5A,B). Notably, given that the *NG2‐CreERT2* line can also drive recombination in the CNS vascular pericytes [19], we examined whether the observed phenotypes involved pericyte alterations using PDGFRβ staining. Our results showed that the density and morphology of PDGFRβ^+^ pericytes remained intact and were indistinguishable between control and *Pdk1* cKO mice (Figure S5C,D). This indicates that vascular pericytes are relatively resistant to PDK1 deficiency compared to the OL lineage in spinal white matter, thereby ruling out vascular structural defects as the contributor to the observed myelin loss and subsequent pain behaviors. BrdU pulse‐labeling assay demonstrated preserved proliferation of OPCs, as evidenced by unchanged proportion of Olig2^+^BrdU^+^/ Olig2^+^ cell numbers (Figure S5E,F). In contrast, the portion of CC1^+^BrdU^+^ cells, newly differentiated OLs, was markedly reduced (Figure S5G,H). These results indicate that PDK1 primarily regulates the differentiation of OPCs into OLs, rather than the progenitor pool.

TUNEL‐based apoptosis quantification showed comparable cell death rates between genotypes (Figure S6A), excluding aberrant survival as a confounding factor. Moreover, IHC verified uncompromised neuronal (NeuN^+^), astrocytic (GFAP^+^), and microglial (IBA1^+^) populations (Figure S6B,C), reinforcing the cell‐autonomous role of PDK1 in OL lineage progression. These findings nominally validate PDK1 as a druggable target for remyelination therapies in NPP disorders.

### 2.5 PDK1 Deficiency Disrupts OL Differentiation and Nascent Myelination via *Sox10*‐Dependent Transcriptional Regulation in the Spinal Cords

**FIGURE 3:**
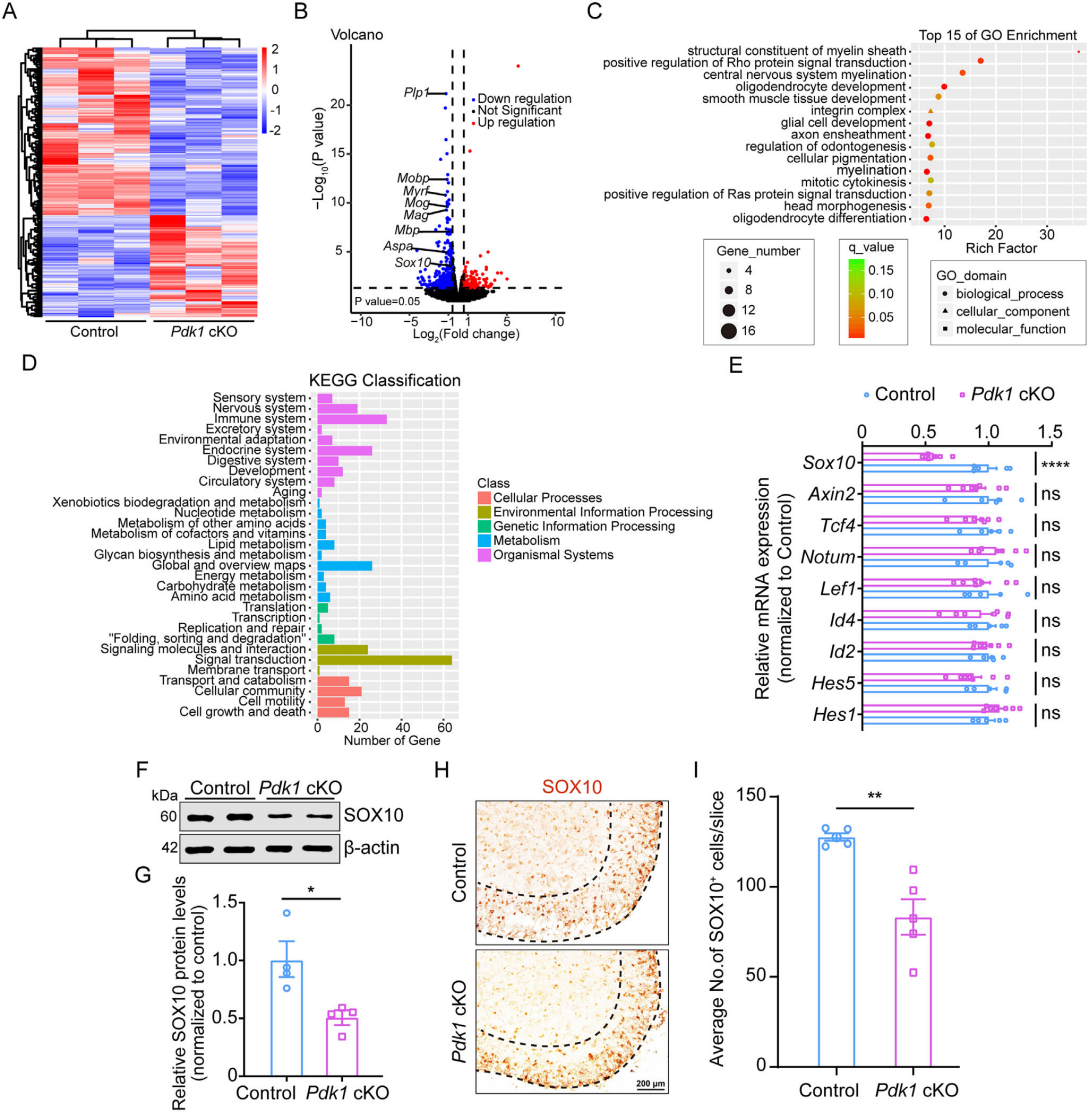
OL lineage‐specific *Pdk1* deletion disrupted OL differentiation and new myelination by downregulating SOX10 in the mouse spinal cords. A. Heatmap illustrated the expression profiles of different genes in the spinal cords of control and *Pdk1* cKO mice. B. RNA‐seq results presented as a volcano plot showing differentially expressed genes between control and *Pdk1* cKO mice. C. GO analysis revealed the enrichment of several signaling pathways in *Pdk1* cKO mice. D. KEGG pathway enrichment analysis reveals significant enrichment of differentially expressed genes in pathways associated with transcription, translation, protein folding, sorting, and degradation. E. qRT‐PCR analysis revealed a decrease in *Sox10*, but no significant changes in the mRNA levels of inhibitory transcription factors, including *Hes1*, *Hes5*, *Id2*, *Id4*, *Lef1*, *Notum*, *Tcf4*, and *Axin2*, in the spinal cords of *Pdk1* cKO mice (unpaired t test; mean ± SEM; n ≥ 5 mice per group). F, G. Representative WB images (F) and quantification (G) of SOX10 in the spinal cords. WB analysis of SOX10 indicated a downregulated expression in the *Pdk1* cKO spinal cords. (unpaired t test; mean ± SEM; n = 4 mice per group). H. IHC staining for SOX10. Scale bar = 200 µm. I. Quantification of SOX10^+^ cells in the ventral and lateral white matter. IHC staining for SOX10 confirmed a marked decrease in SOX10^+^ cells in the spinal cords of *Pdk1* cKO mice (unpaired t test; mean ± SEM; n = 5 mice per group). *p< 0.05; **p< 0.01; ***p< 0.001; ****p< 0.0001; ns, no significant difference.

To elucidate the molecular mechanisms underlying hypomyelination in *Pdk1* cKO mice, we performed RNA‐seq analysis using RNA samples prepared from spinal cord tissues of mice at 2 months. The results revealed 237 upregulated and 391 downregulated genes in *Pdk1* cKO mice compared to littermate controls (Figure 3A). Strikingly, this transcriptional profile demonstrated a significant downregulation of key regulators of OL differentiation and myelination, including the transcription factor gene *Sox10* and its downstream effector genes *Myrf*, *Mbp*, *Plp1*, *Mag*, *Mog*, and *Mobp* (Figure 3B). Gene Ontology (GO) enrichment analysis further corroborated these findings, showing that differentially expressed genes were highly enriched in biological processes essential for OL maturation, myelination, and axon ensheathment (Figure 3C). Kyoto Encyclopedia of Genes and Genomes (KEGG) pathway analysis of the RNA‐seq data revealed pronounced enrichment in transcription, translation, protein folding, and degradation pathways (Figure 3D), suggesting that PDK1 deficiency disrupts OL differentiation at the transcriptional level by inhibiting the expression of a range of myelination‐related genes. Consistent with the RNA‐seq data, qRT‐PCR analysis confirmed a significant reduction in *Sox10* mRNA levels in *Pdk1* cKO mice at 2 months compared to littermate controls (Figure 3E). In contrast, the expression levels of transcriptional repressors, including *Hes1*, *Hes5*, *Id2*, *Id4*, and *Tcf4*, remained unchanged in *Pdk1* cKO mice (Figure 3E). Moreover, WB and IHC analyses further demonstrated decreased SOX10 protein levels in *Pdk1* cKO spinal cords (Figure 3F–I). Given the pivotal role of SOX10 in OL maturation and myelin gene expression, these results indicate that PDK1 regulates OL differentiation in spinal cords via SOX10. The absence of significant changes in OPC proliferation or apoptosis (Figure S5A,B; Figure S6A) reinforces that PDK1 specifically governs the transition from OPCs to mature OLs without affecting cell survival or initial proliferation. Collectively, our findings confirm the critical role of PDK1 in the formation of new myelin fibers.

### 2.6 Oligodendrocytic PDK1 Mediates NPP via the c‐Myc/SOX10 Axis

To elucidate the mechanistic basis of PDK1‐dependent OL differentiation, we examined the phosphorylation status of its downstream targets, Akt and GSK‐3β, in *Pdk1* cKO mice. WB analysis revealed a significant reduction in levels of Akt phosphorylated at Thr308 (p‐Akt^Thr308^) in *Pdk1* cKO mouse spinal cords at 2 months compared to littermate controls (Figure S7A,B). Correspondingly, p‐GSK‐3β^Ser9^ levels were markedly decreased (Figure S7A,B), confirming the inhibition of Akt signaling caused by PDK1 deletion.

**FIGURE 4:**
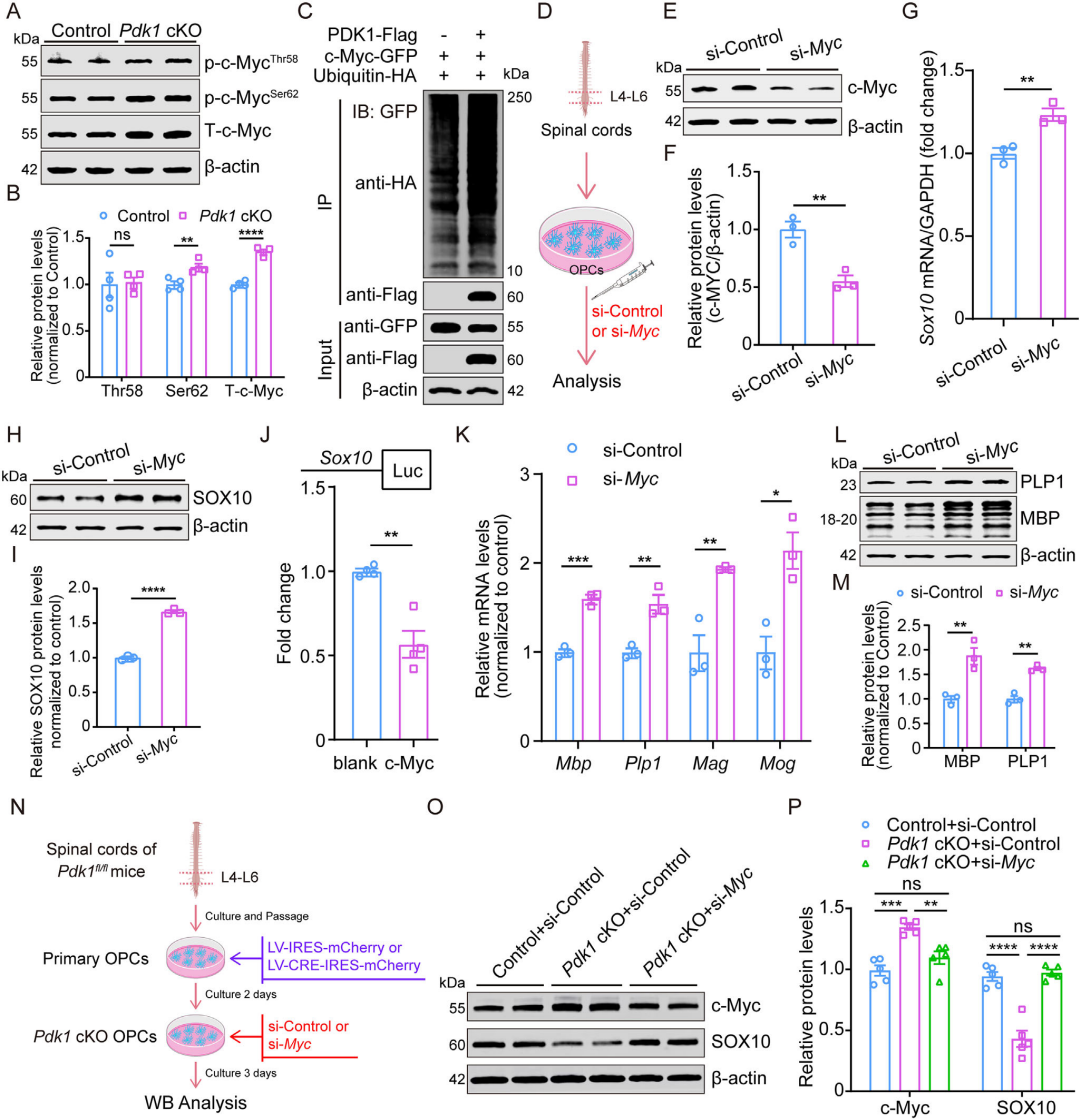
PDK1 deficiency suppressed *Sox10* transcription and myelination via the accumulation of c‐Myc protein during OL development in the spinal cords. A, B. WB analysis (A) and quantification (B) of p‐c‐Myc^Thr58^/T‐c‐Myc, p‐c‐Myc^Ser62^/T‐c‐Myc, and T‐c‐Myc/β‐actin in the spinal cords of control and *Pdk1* cKO mice. Those data revealed the increased ratio of p‐c‐Myc^Ser62^/T‐c‐Myc and T‐c‐Myc/ β‐actin, but no change in the proportion of p‐c‐Myc^Thr58^/T‐c‐Myc in *Pdk1* cKO mice (unpaired t test; mean ± SEM; n = 4 mice per group). C. Co‐IP experiments were used to verify PDK1‐mediated c‐Myc‐specific ubiquitination levels. Lysates from cells co‐transfected with PDK1‐Flag, c‐Myc‐GFP, and Ubiquitin‐HA plasmids were immunoprecipitated using anti‐HA beads, followed by WB with β‐actin, anti‐GFP, and anti‐Flag antibodies. These results showed inhibited ubiquitin degradation in the absence of PDK1 (n = 3 biological replicates for each experiment). D. Schematic illustration of primary OPCs transfection with control or *Myc* siRNA. E, F. Representative WB images (E) and quantification (F) of c‐Myc in the spinal cords. WB validation indicated an efficient silence of c‐Myc (unpaired t test; mean ± SEM; n = 3 biological replicates for each experiment). G. qRT‐PCR analysis of *Sox10* mRNA levels was significantly upregulated following c‐Myc silence compared to the controls (unpaired t test; mean ± SEM; n = 3 biological replicates for each experiment). H. WB images of SOX10 and β‐actin. I. Quantification of SOX10 levels normalized to β‐actin. WB analysis revealed that the protein levels of SOX10 were significantly increased after the silencing of c‐Myc (unpaired t test; mean ± SEM; n = 3 biological replicates for each experiment). J. Luciferase reporter assays demonstrated that c‐Myc suppressed the promoter activity of *Sox10* (unpaired t test; mean ± SEM; n = 4 biological replicates for each experiment). K‐M. qRT‐PCR (K) and WB (L, M) results showed that the expression levels of myelin‐associated genes (e.g., *Mbp*, *Plp1*) were markedly upregulated upon c‐Myc knockdown (unpaired t test; mean ± SEM; n = 3 biological replicates for each experiment). N. Schematic illustration of primary OPC culture, lentivirus infection, and transfection. c‐Myc was silenced in PDK1‐deficient OPCs to assess the expression of SOX10. O. WB images of c‐Myc, SOX10, and β‐actin in primary OPCs cultured in (N). P. Quantification of c‐Myc and SOX10 protein levels. WB analysis indicated that c‐Myc inhibition rescued the downregulation of SOX10 caused by PDK1 deletion (one‐way ANOVA; mean ± SEM; n = 5 mice per group). *p< 0.05; **p< 0.01; ***p< 0.001; ****p< 0.0001; ns, no significant difference.

To further dissect the underlying molecular mechanisms, we wondered whether c‐Myc (encoded by the *Myc* gene) might be a critical mediator for PDK1‐dependent OL differentiation. As a well‐characterized member of the Myc family of proto‐oncogenes, c‐Myc plays important roles in diverse cellular processes, including proliferation, differentiation, and apoptosis [20]. Moreover, it has previously been shown that c‐Myc is involved in OL differentiation [21]. To this end, we conducted the following experiments. While qRT‐PCR analysis showed unchanged *Myc* mRNA levels in *Pdk1* cKO mice at 2 months compared to littermate controls (Figure S7C), WB revealed significantly elevated levels of total c‐Myc protein (T‐c‐Myc) in *Pdk1* cKO mouse spinal cords (Figure 4A,B). Notably, PDK1 deficiency did not affect the ratio of p‐c‐Myc^Thr58^ to T‐c‐Myc (Figure 4A,B), suggesting that the upregulated c‐Myc protein levels were independent of Akt/GSK‐3β signaling in this context. To further exclude the possibility that c‐Myc accumulation was a secondary effect of Akt inhibition, we treated primary OPCs with the Akt‐specific inhibitor MK2206 [22]. Interestingly, WB analysis showed that MK2206 treatment did not lead to an increase in c‐Myc protein levels (Figure S7D), in contrast to the trend observed in *Pdk1* cKO mice. Notably, this differs from the robust c‐Myc accumulation detected in *Pdk1* cKO tissues, suggesting that PDK1 may regulate c‐Myc stability through an Akt‐independent mechanism.

The increase in c‐Myc protein but not mRNA levels prompted us to investigate whether PDK1 directly regulates the turnover of c‐Myc. Using the ScanSite 4.0 prediction tool, we identified Ser62 as the evolutionarily conserved PDK1 recognition site in c‐Myc (Figure S7E). We cultured Oli‐neu cells and overexpressed PDK1 and c‐Myc through plasmid transfection. Co‐immunoprecipitation (Co‐IP) experiments indicated direct interaction between PDK1 and c‐Myc (Figure S7F). In contrast, mutated c‐Myc (T→A at Ser62) significantly disrupted interaction with PDK1 (Figure S7G,H), implicating phosphorylation‐dependent binding. Notably, WB results revealed a significant increase in the p‐c‐Myc^Ser62^/T‐c‐Myc ratio in *Pdk1* cKO mice compared with littermate controls (Figure 4A,B). We next performed ubiquitination assays and found that PDK1 overexpression enhanced c‐Myc ubiquitination (Figure 4C). Simultaneously, we treated cultured cells with either the proteasome inhibitor N‐cbz‐Leu‐Leu‐leucinal (MG132; to block c‐Myc degradation) or the protein synthesis inhibitor cycloheximide (CHX; to inhibit new c‐Myc synthesis). We observed that PDK1 depletion affected c‐Myc protein levels only when synthesis was inhibited (CHX treatment), but not when degradation was blocked (MG132 treatment) (Figure S8). These results establish PDK1 as a critical post‐translational regulator of c‐Myc stability.

To elucidate the relationship between c‐Myc and SOX10, primary OPC cultures were performed (Figure 4D–F). We found that siRNA‐mediated c‐Myc knockdown yielded a pronounced upregulation of SOX10 (Figure 4G–I). Luciferase reporter assay showed that c‐Myc overexpression suppressed *Sox10* promoter activity (Figure 4J). In contrast, c‐Myc knockdown resulted in increased mRNA levels of *Mbp*, *Plp1*, *Mag*, and *Mog* (Figure 4K). WB results confirmed elevated protein levels of MBP and PLP1 upon c‐Myc knockdown (Figure 4L,M). Following the culture and passaging of spinal cord tissue from *Pdk1^fl/fl^* mice to obtain highly pure primary OPCs, these cells were treated with lentivirus (LV‐IRES‐mCherry or LV‐CRE‐IRES‐mCherry) to generate *Pdk1* cKO OPCs, which were subsequently transfected with siRNA (si‐Control or si‐*Myc*) and harvested for protein analysis (Figure 4N). WB results showed restored SOX10 levels in *Myc* siRNA‐treated *Pdk1* cKO OPCs compared with the control group (Figure 4O,P). Together, these data suggest that PDK1 deficiency causes aberrant c‐Myc accumulation via disrupting the Ser62 site phosphorylation and subsequent ubiquitination, leading to repressed SOX10‐dependent myelination programs.

### 2.7 Restoration of Myelination Attenuates NPP in *Pdk1* cKO Mice

**FIGURE 5:**
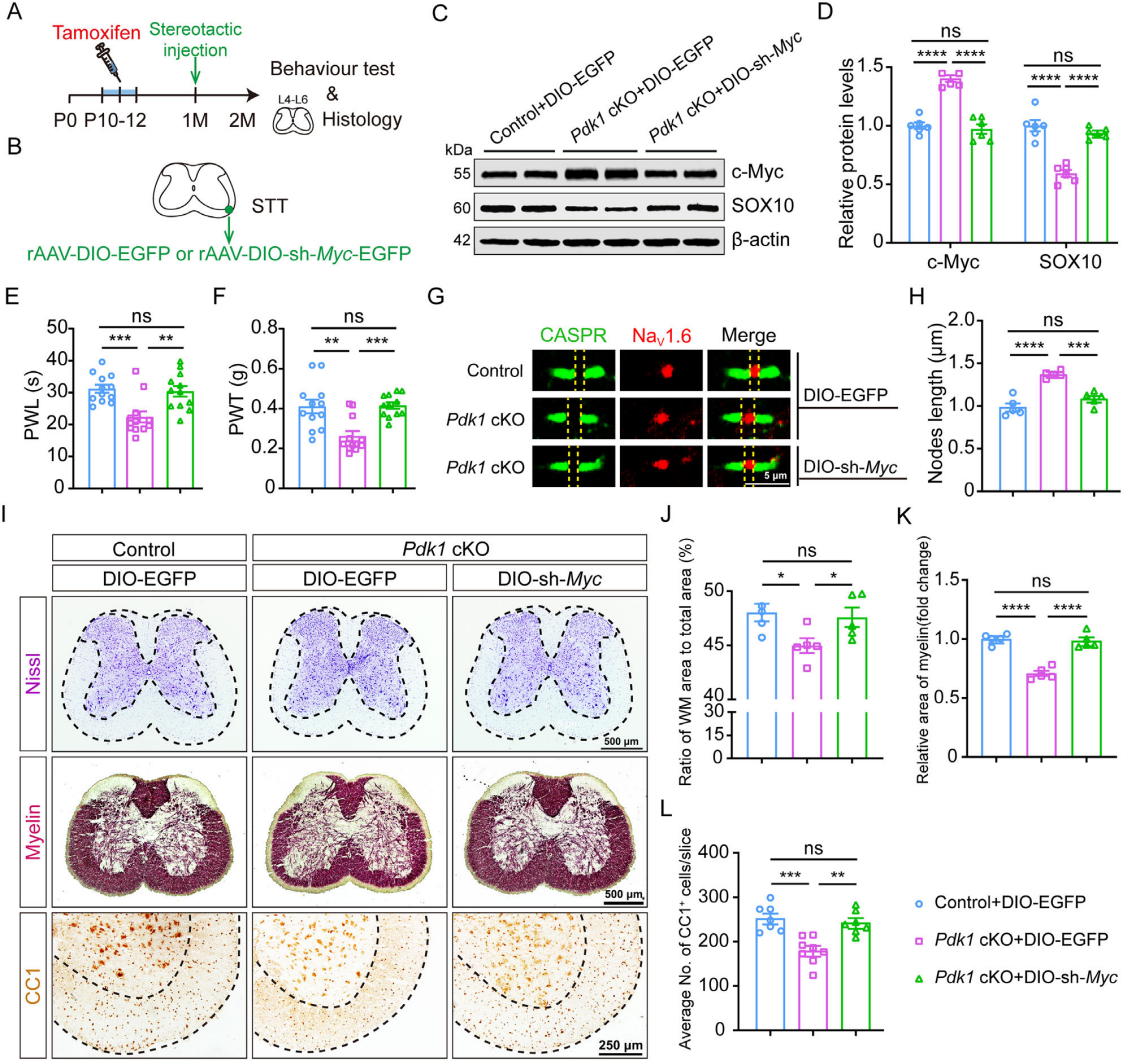
Genetic modulation of c‐Myc in OLs restored myelination and alleviated NPP in *Pdk1* cKO mice. A. Schematic diagram illustrating the experimental timeline. B. Stereotactic viral injections were performed into the ventrolateral white matter of the L4‐L6 spinal cords to target the STT. C, D. Representative WB images (C) and quantification (D) of c‐Myc and SOX10. WB analysis of spinal cord lysates showed that shRNA‐mediated knockdown of c‐Myc significantly reduced both c‐Myc and SOX10 protein levels in *Pdk1* cKO mice compared to control‐treated animals (one‐way ANOVA; mean ± SEM; n = 5 mice per group). E, F. Behavioral tests, including PWL (E) and PWT (F), demonstrated that suppression of c‐Myc in OLs significantly increased pain thresholds in *Pdk1* cKO mice (one‐way ANOVA; mean ± SEM; n = 12 mice per group). G. IHC staining of CASPR (green) and Na_v_1.6 (red) in spinal cords. Scale bar = 5 µm. H. Quantification of node length. IHC staining of Nodes of Ranvier revealed that c‐Myc inhibition restored nodal segment organization in the spinal cords of *Pdk1* cKO mice (one‐way ANOVA; mean ± SEM; n = 5 mice per group). I. Representative images of Nissl (top), Myelin (middle), and CC1 (bottom) staining in the spinal cords. Scale bar = 500 µm and 250 µm. J‐L. Histological analyses further showed that white matter (J), myelin fibers (K), and OL numbers (L) in the ventral and lateral white matter were significantly improved following c‐Myc knockdown, reaching levels comparable to those in control mice (one‐way ANOVA; mean ± SEM; n = 5 mice per group). *p< 0.05; **p< 0.01; ***p< 0.001; ****p< 0.0001; ns, no significant difference.

To test the importance of the oligodendrocytic PDK1/c‐Myc/SOX10 axis in NPP, we employed AAV‐ and Cre‐dependent expression of shRNAs (rAAV‐DIO‐EGFP or rAAV‐DIO‐sh‐*Myc*‐EGFP) to selectively knock down c‐Myc in *Pdk1* cKO spinal cords (Figure 5A,B). Given that the spinothalamic tract (STT), located in the ventrolateral white matter, is the principal ascending pathway for pain signaling [23], we injected AAV‐based constructs into this specific region (Figure 5B). This approach is further supported by studies showing that unilateral STT lesions in the ventrolateral white matter are sufficient to induce persistent thermal hyperalgesia and mechanical allodynia [24, 25, 26], establishing a direct causal link between STT integrity and pain behavior. One month post‐stereotactic injection, the mice were subjected to pain‐related tests followed by spinal cord tissue collections. First, WB confirmed significantly reduced T‐c‐Myc levels in *Pdk1* cKO mice treated with *Myc‐*shRNA (Figure 5C,D). WB also revealed restored SOX10 levels in *Myc* shRNA‐treated *Pdk1* cKO mice (Figure 5C,D). Second, behavioral data showed significant improvements in mechanical and thermal hypersensitivity in *Myc* shRNA‐treated *Pdk1* cKO mice compared with control mice, suggesting successful rescue of NPP phenotypes (Figure 5E,F). Third, IHC analysis further demonstrated that c‐Myc inhibition rescued proper nodal segment organization (Figure 5G,H), consistent with restored nodal structure. In addition, quantitative assessments showed that c‐Myc knockdown restored white matter content, as evidenced by increased myelin fibers and mature OLs density (Figure 5I–L), indicating functional remyelination. Taken together, these data demonstrate that PDK1 deficiency drives NPP progression through a c‐Myc/SOX10‐dependent mechanism that compromises OL maturation and myelination.

**FIGURE 6:**
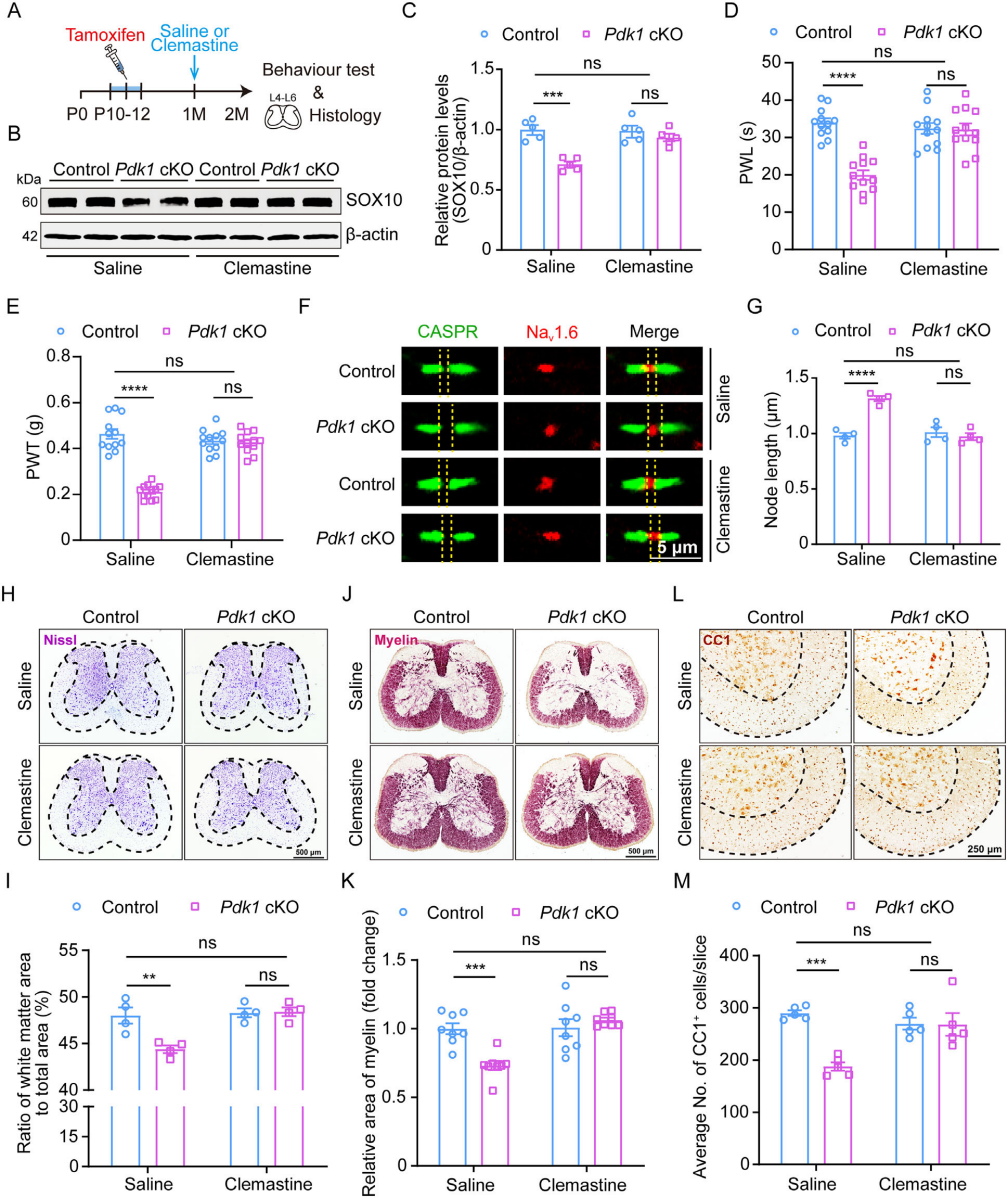
Clemastine enhanced myelination and alleviated pain hypersensitivity in *Pdk1* cKO mice via pharmacological modulation of SOX10 expression. A. Experimental timeline showing that saline or clemastine was administered via intraperitoneal injection to the control or *Pdk1* cKO mice for one month. Behavioral and histological assessments were performed post‐treatment. B. Representative WB images of SOX10. C. Quantification of SOX10 protein levels, as normalized to β‐actin. WB analysis revealed that clemastine treatment restored the expression levels of both SOX10 in the spinal cords of *Pdk1* cKO mice to levels comparable to those in control mice (two‐way ANOVA; mean ± SEM; n = 5 mice per group). D, E. PWL (D) and PWT (E) tests demonstrated that clemastine significantly improved pain thresholds in *Pdk1* cKO mice, indicating a marked attenuation of NPP (two‐way ANOVA; mean ± SEM; n ≥ 11 mice per group). F. IHC staining of CASPR (green) and Na_v_1.6 (red) in the spinal cords. Scale bar = 5 µm. G. Quantification of node length in the spinal cord white matter. IHC staining of the Nodes of Ranvier showed that clemastine treatment restored nodal segment organization in the spinal cords of *Pdk1* cKO mice (two‐way ANOVA; mean ± SEM; n = 4 mice per group). H‐M. Representative images and quantification of Nissl (H, I), Myelin (J, K), and CC1 (L, M) staining in the spinal cords. Histological analyses revealed that the proportion of white matter, myelin fibers, and OL numbers was significantly improved following clemastine treatment, reaching levels comparable to those in control mice (two‐way ANOVA; mean ± SEM; n ≥ 4 mice per group). Scale bar = 500 µm and 250 µm. *p< 0.05; **p< 0.01; ***p< 0.001; ****p< 0.0001; ns, no significant difference.

To validate the role of PDK1‐dependent myelination in NPP, clemastine, previously shown to enhance remyelination in both human multiple sclerosis (MS) patients and mouse demyelination models [27], was used to treat *Pdk1* cKO mice. We administered clemastine into mice via intraperitoneal injection for one month (Figure 6A). WB analysis of spinal cord lysates revealed that clemastine treatment effectively restored SOX10 protein levels to those of control littermates (Figure 6B,C). Behavioral assessments demonstrated a significant reversal of mechanical and thermal hypersensitivity in *Pdk1* cKO mice treated with clemastine compared with those treated with saline, indicating attenuation of NPP (Figure 6D,E). IHC analysis of Nodes of Ranvier confirmed that clemastine restored nodal segment integrity, suggesting functional repair of myelin sheath continuity (Figure 6F,G). Additionally, histological quantification revealed that the white matter area, myelin fibers, and OL density were rescued in the spinal cords of *Pdk1* cKO mice by clemastine (Figure 6H–M). Those findings suggest that clemastine restores myelin homeostasis and mitigates NPP progression in *Pdk1* cKO mice.

While clemastine demonstrated potent analgesic effects in *Pdk1* cKO mice, we sought to definitively rule out potential off‐target effects. We employed a Cre‐dependent rAAV‐DIO‐taCaspase3‐EGFP in *NG2‐CreERT2* mice to selectively ablate the OL lineage cells in the STT region (Figure S9A,B). Notably, the analgesic efficacy of clemastine (administered daily for 21 days post‐injection) was completely abolished in mice lacking functional OLs, whereas it remained effective in control littermates when tested at the 21‐day time point (Figure S9C,D). This loss‐of‐function evidence establishes that clemastine alleviates NPP specifically through an OL‐dependent mechanism.

### 2.8 Targeting c‐Myc in OLs Alleviates NPP and Promotes Remyelination in CCI Mice

**FIGURE 7:**
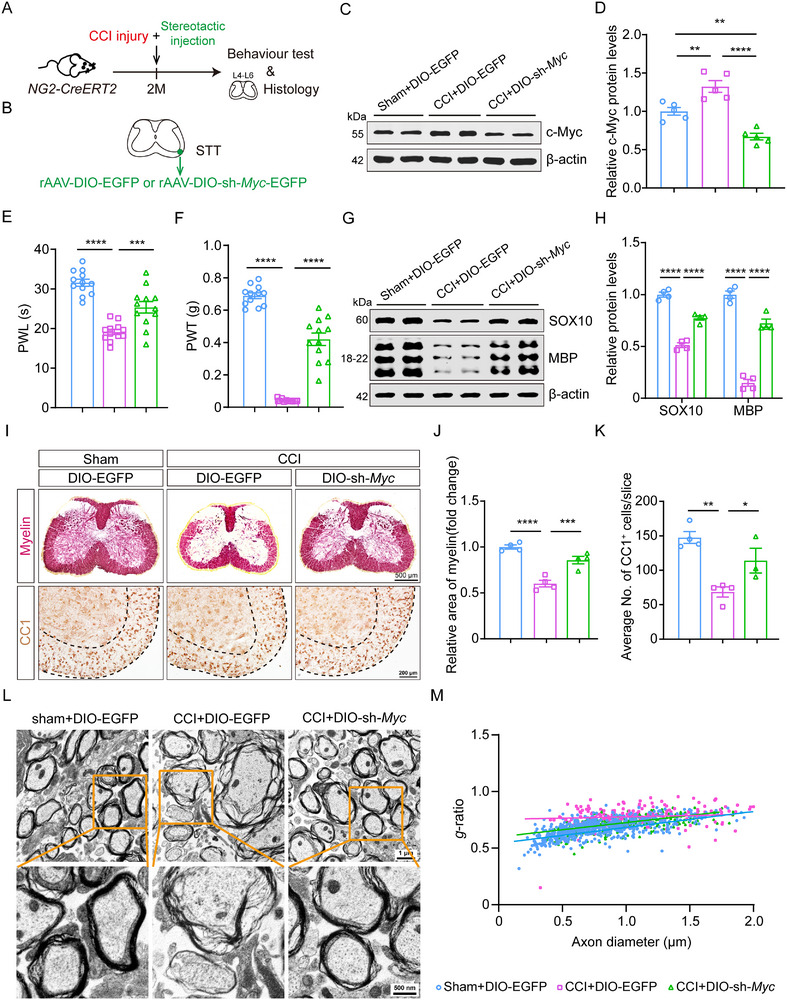
The c‐Myc/SOX10 axis modulated pain sensitivity and myelination in the spinal cords of CCI mice. A. Experimental timeline of viral manipulation and behavioral testing in sham and CCI mice. B. Stereotactic injection of viral constructs into the ventrolateral white matter of L4‐L6 mouse spinal cords. C, D. Representative WB (C) and quantitative analysis (D) of c‐Myc expression in the spinal cords. c‐Myc was markedly upregulated in CCI mice, and efficiently silenced by sh‐*Myc*‐rAAV treatment (one‐way ANOVA; mean ± SEM; n = 5 mice per group). E, F. Behavioral analyses of PWL (E) and PWT (F). Both the pain thresholds were significantly improved in sh*‐Myc*‐rAAV‐treated CCI mice compared with rAAV control‐treated CCI mice, indicating attenuated pain hypersensitivity (one‐way ANOVA; mean ± SEM; n = 10 mice per group). G. Representative WB images of SOX10 and MBP in spinal cords. H. Quantification of SOX10 and MBP levels normalized to β‐actin. WB validation revealed that the protein levels of SOX10 and MBP were significantly increased after the silencing of c‐Myc in CCI mouse spinal cords (one‐way ANOVA; mean ± SEM; n = 4 mice per group). I‐K. Representative images and corresponding quantification of Myelin (I, J), and CC1 (I, K) immunostaining in the ventral and lateral white matter of mouse spinal cords. Histological analysis revealed that knockdown of c‐Myc in CCI mice significantly increased both the area of myelinated fibers and the number of CC1^+^ OLs, restoring them to levels comparable with sham controls (one‐way ANOVA; mean ± SEM; n ≥ 3 mice per group). Scale bar = 500 µm and 200 µm. L, M. Representative TEM images (L) and corresponding *g*‐ratio analysis (M) of myelinated axons in the ventral and lateral white matter. c‐Myc knockdown alleviated myelin structural abnormalities and increased myelin thickness in CCI mice, resulting in a reduced *g*‐ratio and indicative of enhanced remyelination in CCI mice's ventral and lateral white matter (Sham+DIO‐EGFP: *g*‐ratio = 0.675146; CCI+DIO‐EGFP: *g*‐ratio = 0.7817; CCI+DIO‐sh‐*Myc*: *g*‐ratio = 710737) (one‐way ANOVA; mean ± SEM; n = 3 mice per group). Scale bar = 1 µm and 500 nm. *p< 0.05; **p< 0.01s; ***p< 0.001; ****p< 0.0001; ns, no significant difference.

To evaluate whether the PDK1/c‐Myc/SOX10 axis contributes to NPP pathogenesis in the CCI model, we next asked whether modulating c‐Myc expression could reverse NPP‐associated behaviors and myelin defects in CCI mice. We stereotactically injected rAAV‐DIO‐sh‐*Myc*‐EGFP or rAAV‐DIO‐EGFP into the ventrolateral white matter of L4‐L6 spinal cord segments of CCI and sham‐operated mice (Figure 7A,B). WB analysis confirmed that c‐Myc protein levels were significantly elevated in the spinal cords of CCI mice compared to sham controls, and this upregulation was effectively reversed by rAAV‐sh‐*Myc* treatment (Figure 7C,D). Behavioral assessments showed that CCI mice injected with the control vector exhibited pronounced mechanical allodynia and thermal hyperalgesia, as reflected by decreased PWL and PWT thresholds (Figure 7E,F). In contrast, knockdown of oligodendrocytic c‐Myc significantly ameliorated these pain hypersensitivities, restoring pain thresholds toward levels observed in the sham mice (Figure 7E,F).

At the molecular level, c‐Myc silencing markedly increased the protein expression of SOX10 and MBP in CCI mice (Figure 7G,H), supporting the hypothesis that c‐Myc acts as a transcriptional repressor of *Sox10* under NPP conditions. Consistent with these findings, histological analyses demonstrated that silencing c‐Myc robustly promoted OL differentiation and enhanced myelin integrity in the spinal white matter of CCI mice, reaching levels comparable to those in sham controls (Figure 7I–K). To further evaluate ultrastructural myelin recovery, we performed transmission electron microscopy (TEM). Compared with the sham control group, CCI mice exhibited markedly thinner myelin sheaths, an increased *g*‐ratio, and disrupted myelin architecture, indicative of myelin dysplasia (Figure 7L,M). Notably, knockdown of c‐Myc markedly enhanced myelin sheath thickness and reduced the *g*‐ratio (Figure 7L,M), thereby restoring myelin structural integrity and supporting the notion that c‐Myc inhibition promotes remyelination in CCI mice.

Collectively, these results identify c‐Myc as a pivotal regulator of NPP pathogenesis. Its selective knockdown in OL lineage cells reinstates SOX10‐driven myelination and preserves myelin integrity, ultimately mitigating behavioral hypersensitivity during the remyelination phase following CCI.

## 3 Discussion

NPP has traditionally been conceptualized as a sensory processing disorder, driven largely by neuronal hyperexcitability and astrocytic reactivity [28]. While the contributions of neurons and astrocytes to NPP are well documented, the role of OLs and their myelin sheaths remains comparatively understudied [16, 29]. Given the essential role of OLs in enabling saltatory conduction along myelinated axons and at the Nodes of Ranvier, their dysfunction can compromise neural circuit integrity, thereby precipitating or exacerbating NPP [30]. Here, we identify OL lineage‐specific PDK1 deficiency as a key pathogenic mechanism that disrupts OL differentiation, impairs myelination, and contributes to NPP pathology.

Animal models of CCI have provided valuable insight into glial contributions to NPP. CCI triggers robust activation of spinal microglia and astrocytes, leading to inflammatory cytokine release and oxidative stress that culminate in myelin pathology, including focal demyelination, myelin decompaction, and loss of OL lineage cells [17, 31]. Despite being the major neural phenotype implicated in the CNS, OLs have been predominantly studied for their molecular regulation of myelination [32], with their functional contribution to pain modulation remaining largely unexplored. Myelin sheaths not only preserve axonal integrity but are also indispensable for nodal maturation, rapid impulse propagation, and temporal synchronization of neuronal firing [33, 34, 35]. Notably, OL ablation on the dorsal horn using diphtheria toxin results in nociceptive hypersensitivity and axonal degeneration [36], emphasizing that OL dysfunction directly contributes to NPP. However, the molecular mechanisms linking OL and myelin pathology in the spinal white matter to NPP remain unclear.

In our study, we observed substantial demyelination and OL‐lineage cell loss in the ipsilateral spinal white matter after CCI injury. Interestingly, the PDK signaling pathway in spinal white matter OL lineage cells was selectively downregulated 14 days after CCI, but not at earlier stages, suggesting its primary involvement in myelin maintenance or regeneration rather than the acute injury response. This temporal pattern is consistent with previous work, which demonstrated that pain behaviors can arise prior to overt demyelination [36]. In that study, early loss of mature OLs and downregulation of myelin‐associated genes preceded the appearance of mechanical and cold hypersensitivity, whereas profound MBP loss occurred at later stages. Together, these findings support a model in which early OL dysfunction, characterized by cellular loss, transcriptional dysregulation, and compromised myelin integrity, can initiate NPP, while progressive demyelination and failed remyelination contribute to the persistence and amplification of pain states. To directly dissect the contribution of OL‐intrinsic signaling to NPP, we generated inducible OL lineage‐specific *Pdk1* cKO mice. Remarkably, these mice recapitulated key pathological and behavioral features of CCI, including OL loss, hypomyelination, and persistent mechanical and thermal hypersensitivity, in the absence of overt axonal degeneration. These results establish a direct causal link between OL‐intrinsic PDK1 deficiency and both structural and functional manifestations of NPP. Given PDK1's established role as an upstream kinase regulating prosurvival, metabolic, and cytoskeletal pathways [7, 8, 37], loss of PDK1 in OLs is likely to disrupt axon‐glia metabolic coupling, impair cytoskeletal organization, and compromise myelin stability. Such alterations may perturb axonal conduction and nodal integrity, thereby enhancing neuronal excitability and lowering sensory thresholds. Notably, the temporal overlap between progressive myelin disruption and sustained sensory hypersensitivity in *Pdk1* cKO mice supports a model in which PDK1 functions as a molecular safeguard that preserves myelin integrity and protects against the development and maintenance of NPP.

A critical question is how demyelination in the ventrolateral white matter translates into the functional manifestation of NPP. Our findings suggest that dorsal horn neuronal dysfunction is a protracted secondary consequence of ventrolateral white matter demyelination. We observed that structural disruption of the nodes of Ranvier, characterized by nodal elongation of Na_v_1.6 channels along the STT, provides a substrate for ectopic axonal firing [38]. This persistent barrage of aberrant excitatory inputs likely triggers maladaptive synaptic remodeling in the dorsal horn, as evidenced by the reduced density of Synaptophysin and Homer1 puncta. Crucially, we identified a significant reduction in VGAT‐labeled inhibitory terminals in the *Pdk1* cKO dorsal horn. The loss of inhibitory input is a fundamental driver of disinhibition, a state in which local spinal circuits lose their gating control, resulting in enhanced activation of pain‐processing neurons [39, 40, 41]. Since these structural and molecular alterations strongly support a state of heightened neuronal excitability, future investigations using spinal cord slice patch‐clamp recordings will be required to directly measure action potential firing thresholds and excitatory/inhibitory synaptic currents, thereby providing definitive functional confirmation of this proposed mechanism.

Mechanistically, our findings delineate a PDK1/c‐Myc/SOX10 signaling axis that plays a central role in OL differentiation and myelin gene expression. In this pathway, PDK1 regulates c‐Myc protein turnover, thereby influencing *Sox10*‐dependent transcriptional programs required for the expression of key myelin genes, including *Mbp* and *Plp* [42, 43]. Notably, PDK1 deficiency selectively reduced *Sox10* expression without affecting classical differentiation inhibitors (e.g., *Hes1*, *Id2*, *Lef1*), pinpointing a specific regulatory node within the OL differentiation network. Although c‐Myc is classically associated with OPC proliferation, our data reveal a biphasic, stage‐specific role: PDK1 loss alters c‐Myc turnover, converting it from a pro‐proliferative factor into a transcriptional repressor of *Sox10*, thereby stalling terminal differentiation and myelin formation [44]. Remarkably, rAAV‐mediated knockdown of c‐Myc in *Pdk1* cKO and CCI mice restored SOX10 expression, rescued myelin integrity, and partially alleviated pain hypersensitivity, validating this mechanistic axis.

Interestingly, despite a marked reduction in mature OLs, the population size and proliferative activity of OPCs remained largely unchanged in *Pdk1* cKO mice. This finding is notable in light of established differentiation‐dependent feedback mechanisms, whereby loss of mature OLs or myelin typically elicits compensatory OPC recruitment to promote repair [45]. The absence of such a compensatory response in our model suggests a functional uncoupling between OL loss and progenitor amplification. One plausible interpretation is that PDK1‐dependent signaling constitutes an intrinsic regulatory component required for OPCs to effectively engage differentiation‐linked feedback programs. In the absence of PDK1/c‐Myc signaling axis, OPCs may retain basal self‐renewal capacity but fail to appropriately escalate proliferation or complete maturation in response to reduced OL output. This interpretation is consistent with the “set‐point” model of OPC homeostasis [45], in which progenitor density is actively maintained even under pathological conditions, rather than undergoing unchecked expansion. Moreover, recent studies have demonstrated that differentiation arrest does not invariably lead to net OPC accumulation, particularly in chronic disease states where lineage progression, migration, and age‐related cell cycle slowing dynamically shape progenitor behavior [46, 47]. Under such conditions, modest basal OPC proliferation may be counterbalanced by failed maturation attempts and subtle lineage attrition, resulting in a numerically stable yet functionally compromised progenitor pool. Together, these considerations support the notion that impaired differentiation, rather than depletion or hyperproliferation of OPCs, represents the dominant lineage defect downstream of PDK1 loss.

Nevertheless, several important questions remain to be addressed. It is yet to be determined whether PDK1 signaling interacts with other pain‐modulatory pathways, such as ERK/MAPK or NF‐κB signaling, within OL lineage cells, or whether adult‐onset PDK1 deletion recapitulates the phenotypes observed in juvenile models. In addition, determining whether similar PDK1‐dependent mechanisms operate across distinct NPP models, including spinal nerve ligation, chemotherapy‐induced neuropathy, or diabetic neuropathy, will be essential to assess the generalizability of our findings. Another limitation of the present study is the lack of direct validation of the proposed PDK1/c‐Myc/SOX10 signaling axis in human NPP conditions or patient‐derived samples. Our mechanistic conclusions are primarily derived from preclinical rodent models, which, while invaluable for elucidating fundamental molecular and cellular mechanisms, may not fully capture the complexity and heterogeneity of human NPP. Consequently, it remains uncertain whether dysregulation of this signaling axis occurs in human disorders such as diabetic neuropathy, post‐herpetic neuralgia, or chemotherapy‐induced peripheral neuropathy. Future studies incorporating well‐characterized post‐mortem human spinal cord tissues, patient‐derived cellular models, or translational datasets will therefore be critical to validate the pathological relevance and therapeutic potential of the PDK1/c‐Myc/SOX10 axis in human NPP.

In summary, our findings identify PDK1‐dependent OL dysfunction as a key driver of NPP, mediated by disruption of the c‐Myc/SOX10 regulatory axis. By integrating structural (myelin maintenance) and functional (nociceptive sensation) aspects, PDK1 emerges as a pivotal regulator of chronic pain. This work broadens the mechanistic framework of central sensitization and positions OL‐targeted interventions as viable therapeutic avenues for chronic pain and potentially for demyelinating disorders.

## 4 Methods Section

### 4.1 Animals

In the present study, male and female mice of the C57BL/6 genetic background were utilized. Animals were group‐housed in a density of 4–5 per cage within the animal housing facility of the Model Animal Research Center (MARC) at Nanjing University. The mice were maintained under standardized conditions, including a 12‐h light‐dark cycle, ad libitum access to food and water, and a controlled ambient temperature of 25 ± 1°C. All experimental procedures, including breeding operations and surgical interventions, were conducted in strict accordance with the guidelines for the Care and Use of Laboratory Animals established by MARC, Nanjing University, and were formally approved by the Institutional Animal Care and Use Committee (IACUC) of the center. This ensured ethical compliance throughout the research process. Animal Experimentation License Number: 220203203.

The inducible *Pdk1* cKO mice (*Pdk1^fl/fl^;NG2‐CreERT2*) employed in this study were generated through a systematic multi‐step breeding strategy. Initially, mice carrying the floxed *Pdk1* allele (*Pdk1^fl/fl^*) [48] were interbred with *NG2‐CreERT2* floxed mice [14, 19] to produce *Pdk1^fl/fl^;NG2‐CreERT2* mice. Subsequently, these mice were crossed with *mTmG* floxed mice [49] to establish the triple‐transgenic *Pdk1^fl/fl^;NG2‐CreERT2;mTmG* lineage. The mTmG strain facilitates Cre‐mediated recombination monitoring, as it expresses membrane‐targeted tdTomato (mT) prior to Cre activation and switches to membrane‐targeted EGFP (mG) post‐recombination [49], thereby enabling straightforward assessment of Cre excision efficiency. To induce conditional gene deletion, neonatal C57BL/6J mice (postnatal days 10–12) were administered intraperitoneal injections of tamoxifen at 50 mg/kg daily for three consecutive days. The pups were maintained under maternal care throughout the experimental period.

In addition, *Pdk1^fl/fl^;Olig1‐Cre* mice were generated by crossing *Pdk1^fl/fl^* mice with *Olig1‐Cre* transgenic mice [14]. The detailed procedures were performed as described above.

### 4.2 CCI Model

The CCI model [50] was employed to induce NPP in experimental mice. For surgical induction, 2‐month‐old mice were anesthetized using 2% isoflurane (Harvard Apparatus) in oxygen. Subsequently, the left sciatic nerve was exposed in the mid‐thigh region via blunt dissection of the surrounding musculature, followed by ligation with four loose silk sutures (5‐0 chromic gut) arranged at 1 mm intervals. The incision site was then closed with sterile sutures. In the sham‐operated control group, mice underwent identical surgical procedures, including skin incision and nerve exposure, but without application of the ligatures. This standardized approach ensured consistent experimental conditions while isolating the effects of nerve injury from surgical manipulation.

### 4.3 Behavioral Testing

#### 4.3.1 NPP behaviors, including mechanical allodynia and thermal hyperalgesia, were assessed post‐surgically on day 14 using established protocols [51]

For mechanical sensitivity evaluation, the PWT was determined via sequential application of calibrated Von Frey filament to the plantar surface of the left hind limb. Testing followed the modified Dixon's “Up‐Down” method [52], with mice acclimatized in wire‐mesh enclosures for 30 min before each session. A three‐day pre‐testing acclimation period was conducted before initiating formal behavioral measurements. Each filament was applied at least three times with inter‐stimulus intervals exceeding 5 min to ensure reliable threshold determination.

Thermal hyperalgesia was quantified using PWL measured on an automated hot plate analgesia meter. Mice underwent identical 30‐min acclimation procedures in transparent glass chambers before testing. The device was preheated to either 47°C or 55°C, and mice were removed automatically upon hind paw withdrawal or after a 60‐second timeout to prevent tissue damage. Three replicate measurements were taken per session with standardized inter‐test intervals, and the 30‐min waiting period between trials was strictly maintained to avoid sensitization effects. This dual assessment approach provided a comprehensive characterization of NPP phenotypes while minimizing procedural variability through rigorous standardization.

#### 4.3.2 Spontaneous scratching test

Spontaneous scratching behavior was evaluated as previously described [53, 54]. Mice were individually placed in transparent acrylic observation chambers for a 30‐min habituation period, followed by a 30‐min video‐recorded session under controlled lighting and temperature conditions. A scratching bout was defined as continuous hindpaw movements directed toward a specific body region and ending with the paw being placed back on the floor or mouth. Recordings were analyzed offline by an experimenter blinded to genotype and treatment. The total number of scratching bouts per 30 min was quantified and used as an index of spontaneous itch‐like behavior.

#### 4.3.3 OFT

Locomotor activity was evaluated using OFT [55]. Each mouse was placed in the center of a square arena with a white floor under uniform illumination. Movement was recorded for 5 min using a video‐tracking system. Total distance traveled and average speeds were automatically calculated. Between sessions, the arena was cleaned with 70% ethanol to eliminate odor cues.

#### 4.3.4 BBW

Fine motor coordination and balance were further assessed using the BBW test [46]. Mice were trained to traverse a 1‐meter‐long horizontal wooden beam (1.5 centimeters in diameter) elevated 1 meter above the floor with a dark goal box at one end. The task consisted of two training days and one testing day. During each training day, mice were trained to cross the beam and were required to complete three successful trials. Trials were separated by 30‐min intervals, with a maximum of five trials per day. On the testing day, each mouse performed three consecutive trials. The latency to traverse the beam and the number of foot slips were recorded by an experimenter blinded to group allocation. To minimize olfactory interference, the beam and goal box were cleaned with 70% ethanol between trials.

#### 4.3.5 RT

Motor coordination and balance were also assessed using an accelerating rotarod apparatus as previously described [46]. Mice were pre‐trained for three consecutive days to acclimate to the apparatus. During training, mice initially walked at a constant speed of 10 rpm for up to 5 min, followed by an accelerating protocol that started at 4 rpm with an increment of 1 rpm every 10 seconds, reaching a maximum of 40 rpm. Three trials were conducted per day with 30‐min intervals between trials. On the fourth day, formal testing was performed under the same accelerating conditions. The latency to fall was recorded for three consecutive trials, and the mean value was used for statistical analysis. The rod was disinfected with 70% ethanol between trials.

### 4.4 WB

NPP‐related spinal cord tissues were harvested and processed for WB analysis following standardized protocols similar to RNA sample collection procedures. Spinal cords were rapidly dissected under sterile conditions and snap‐frozen in liquid nitrogen to preserve protein integrity. Protein extraction was performed by homogenizing tissues in radioimmunoprecipitation assay (RIPA) lysis buffer (20 mM Tris‐HCl, pH 7.4; 150 mM NaCl; 1 mM EDTA; 1% NP‐40; 0.5% sodium deoxycholate; 0.1% SDS) supplemented with 1% protease inhibitor cocktail and 1% phosphatase inhibitor (A and B solutions). Samples were incubated on ice for 30 min before centrifugation at 4°C to isolate the soluble protein fraction. Protein concentrations were determined using a Bradford assay with BSA standards, and 40 µg of total protein was loaded onto SDS‐PAGE gels for electrophoretic separation. Proteins were transferred onto nitrocellulose (NC) membranes via semi‐dry electroblotting, followed by blocking with 5% non‐fat milk in TBST at room temperature for 60 min. Membranes were incubated overnight at 4°C with primary antibodies (detailed specifications in Table S1), then washed three times with TBST and incubated with fluorescently‐labeled secondary antibodies (1:5000 dilution) for 60 min at ambient temperature. Post‐incubation, membranes were extensively washed and imaged using a Li‐Cor Odyssey CLx infrared imaging system to quantify protein expression levels with high sensitivity and precision. This rigorous workflow ensured optimal signal detection while minimizing experimental variability through strict adherence to standardized protocols.

### 4.5 IHC

Spinal cord segments (L4‐L6) were fixed in 4% paraformaldehyde (PFA) solution at 4°C overnight to preserve tissue architecture. Following fixation, tissues underwent sequential dehydration either through immersion in sucrose gradient solutions or via paraffin embedding procedures. For cryosectioning, PFA‐fixed tissues were sucrose gradient‐dehydrated and embedded in optimal cutting temperature (OCT) compound, snap‐frozen in liquid nitrogen, and stored at ‐80°C until sectioning (12 µm thickness per slice). Paraffin‐processed tissues were dehydrated through a graded ethanol series, embedded in paraffin blocks, and sectioned at 10 µm thickness. All sections were subjected to antigen retrieval by incubation in 0.01 M citrate buffer (pH 6.0) using microwave heating, followed by PBS rinses and blocking with 5% bovine serum albumin (BSA) in PBS for 60 min at room temperature. Primary antibodies (detailed in Table S1) were applied to the sections and incubated overnight at 4°C (16–20 h). After thorough PBS washing (three times, 5 min each), fluorescent secondary antibodies (1:1000 dilution in blocking solution) were applied for 1 h at room temperature in the dark to prevent photobleaching. Nuclear counterstaining was performed using 4',6‐diamidino‐2‐phenylindole (DAPI), and all staining procedures strictly adhered to standardized protocols to ensure reproducibility.

IHC imaging was acquired using either Olympus BX53 bright‐field microscopy, SS‐MCS Microscopic Confocal Scanning System, Leica TCS SP5 laser confocal microscopy, or ZEISS LSM880 High‐Precision Laser Confocal Microscopy System for high‐resolution fluorescent analysis. The spinal regions (ventral and lateral white matter) of experimental and control mice were imaged under identical settings to maintain consistency. All primary and secondary antibodies employed in these analyses are fully characterized in Table S1, with validated specificity and optimal dilutions confirmed through preliminary titration experiments.

### 4.6 qRT‐PCR

Spinal cord tissues (L4‐L6 segments) were harvested under sterile conditions following anesthesia with 2% isoflurane in oxygen. Immediately after dissection, samples were either processed for RNA extraction or snap‐frozen in liquid nitrogen and stored at ‐80°C for future use. Total RNA was isolated using RNAiso Plus reagent (Takara) according to the manufacturer's protocol, with RNA quality and concentration verified via NanoDrop spectrophotometry. Approximately 1 µg of high‐quality RNA was reverse‐transcribed into cDNA using the HiScript II Q RT SuperMix for qPCR (+gDNA wiper) (Vazyme), with all reactions including RNase‐free DNase treatment to eliminate genomic DNA contamination. The resulting cDNA was subsequently utilized as a template for qRT‐PCR analysis, employing glyceraldehyde‐3‐phosphate dehydrogenase (GAPDH) as the housekeeping gene for normalization.

qRT‐PCR experiments were performed on a Roche LightCycler 96 instrument using ChamQ SYBR qPCR Master Mix (Vazyme), with cycling parameters optimized for maximal amplification efficiency. Relative gene expression levels were calculated using the 2^−ΔΔCt^ method (Livak method) to quantify mRNA fold changes compared to control conditions. All primers were designed using Primer5 software with stringent criteria (amplicon length 100–150 bp, GC content 50–60%, melting temperature 58–60°C), and their sequences are listed in Table S2. Primers were custom‐synthesized by Qingke Biology (Nanjing, China) and Bioligo Biotechnology (Shanghai, China). Experimental conditions included two or three technical replicates per sample to ensure assay specificity. This standardized workflow incorporated rigorous quality controls at each stage to ensure reliable quantification of target gene expression in NPP models.

### 4.7 TrueGold Myelin Staining

Frozen spinal cord sections were equilibrated to room temperature before staining. Sections were air‐dried at 37°C for 30 min to remove residual moisture, then immersed in TrueGold myelin staining solution (Oasis) and incubated at 45°C for 20–30 min in the dark to achieve optimal myelin staining. This protocol enabled the distinct visualization of slender myelinated fibers under light microscopy. Following staining, the sections underwent three sequential washes with distilled water (three times, each for 1 min). The staining reaction was terminated by applying dye termination solution (Oasis), which was allowed to act for 2–3 min at 45°C. Sections were then rinsed three additional times with distilled water (three times, 1 min each), mounted with aqueous mounting medium, and imaged using a bright‐field microscope with standardized magnification and exposure settings. All procedural parameters were strictly controlled to ensure consistent staining quality and reproducibility of morphological assessments.

### 4.8 Nissl Staining

The sections were deparaffinized and rehydrated, stained with 1% cresyl‐violet for 1 min, and rinsed with ddH_2_O twice, 1 min each time. After the sections were dried, sealed the sections with neutral resin and captured images.

Deparaffinized spinal cord sections were subjected to a standardized cresyl violet staining protocol for analysis. Following xylene‐based dewaxing and rehydration through a graded ethanol series (100%, 95%, 70%), sections were immersed in cresyl‐ciolet Acetate staining solution (1% w/v in distilled water, Sigma–Aldrich) for 1 min at room temperature. After staining, tissues were rinsed twice with distilled water (three times, 1 min each) to remove excess dye, then air‐dried at room temperature for 20 min to ensure complete solvent evaporation. Sections were mounted with neutral balsam (MeilunBio), then imaged using a bright‐field microscope with standardized magnification and exposure settings. All staining parameters, including dye concentration and incubation duration, were optimized through preliminary titration experiments to ensure consistent nuclear staining intensity and cytoplasmic contrast.

### 4.9 TUNEL Staining

Deparaffinized spinal cord sections underwent a standardized terminal deoxynucleotidyl transferase dUTP nick end labeling (TUNEL) assay for apoptosis detection using the BrightGreen Apoptosis Detection Kit (Vazyme). Following xylene dewaxing and rehydration through a graded ethanol series (100% to distilled water), tissue sections were processed according to the manufacturer's protocol. The TUNEL reaction mixture was applied to the sections and incubated at 37°C in the dark for 60 min to label DNA fragmentation in apoptotic cells. Nuclei were subsequently counterstained with DAPI for 10 min at room temperature to visualize cellular morphology. Apoptotic cells were identified as those exhibiting co‐localized TUNEL^+^ green fluorescence and DAPI‐stained nuclei, representing double‐labeled apoptotic cells. Used confocal microscopy to capture the images. This rigorous workflow incorporated validated reagents, strict adherence to dark incubation conditions, and standardized imaging parameters to ensure reliable quantification of apoptosis in mice.

### 4.10 TEM

Mice were deeply anesthetized with 2% isoflurane, and the spinal cords (L4‐L6) were rapidly dissected. The tissues were immediately fixed in 2.5% glutaraldehyde in 0.1 M phosphate buffer (pH 7.4) at 4 °C overnight. After fixation, the samples were transferred to Lilai Biological Technology Co., Ltd. (Chengdu, China) for subsequent processing, including post‐fixation with 1% osmium tetroxide, dehydration through a graded ethanol series, resin embedding, and ultrathin sectioning. Ultrathin sections were stained with uranyl acetate and lead citrate and examined under a transmission electron microscope. Representative images were captured for morphological evaluation, and axon diameter, myelin thickness, and g‐ratio were quantified using ImageJ software.

### 4.11 BrdU Label Assay

BrdU labeling was employed in two distinct experimental paradigms to assess OL lineage dynamics.

For differentiation analysis, mouse pups received three consecutive intraperitoneal BrdU injections (100 mg/kg body weight) at P12‐14. Following perfusion fixation, spinal cord sections were processed using standardized immunofluorescence protocols with overnight incubation at 4°C in CC1 and BrdU antibodies. Co‐localization of CC1^+^BrdU^+^ signals was quantified via confocal microscopy, indicating successful differentiation of OLs from OPCs during this critical developmental window.

In the proliferation assessment, a single BrdU injection (100 mg/kg, MCE) was administered at postnatal day 10, with spinal cord tissues harvested 1 h later to capture ongoing S‐phase events. Sections were incubated with Olig2 (OPC marker) and BrdU antibodies using a 2‐h room temperature protocol, followed by fluorescent secondary antibodies. Quantification of Olig2^+^BrdU^+^ double‐labeled cells revealed the proliferative activity of OPCs at this early developmental stage. These temporally distinct BrdU labeling strategies allowed precise dissection of OPC differentiation and proliferation dynamics in the developing spinal cords under NPP conditions.

### 4.12 Tamoxifen Administration

Tamoxifen (10 mg/mL stock solution, MCE) was prepared in corn oil to prevent photodegradation, with all handling steps conducted in a light‐shielded environment. Neonatal mice (P10‐P12) received intraperitoneal injections of tamoxifen (50 mg/kg body weight) for conditional gene deletion in OL lineage cells. The tamoxifen solution was freshly prepared before each injection and stored in amber glass vials between uses. Dosing calculations were based on individual body weight measurements taken daily, with injections administered at the same time of day to maintain temporal consistency. This light‐protected protocol ensured optimal compound stability while enabling precise spatial control of Cre‐mediated recombination in OPCs during early postnatal development. All procedures adhered to MARC's animal welfare guidelines to minimize stress during neonatal handling.

### 4.13 Cell Culture, Transfection, and Lentivirus Infection

Primary OPCs were isolated from the spinal cords of C57BL/6J mice and cultured using a modified version of previously validated protocols [14]. Cells were maintained in serum‐free neurosphere medium supplemented with 2% B27, 1% N2, 5 µM HEPES, 0.01% Heparin, 1% penicillin‐streptomycin, 20 ng/mL epidermal growth factor (EGF), 20 ng/mL basic fibroblast growth factor (bFGF), and 8 ng/mL insulin (all from Sigma–Aldrich). Neurosphere formation was observed within 2–3 days, after which cells were dissociated into single‐cell suspensions and passaged into fresh neurosphere medium. This expansion protocol was repeated twice to achieve a population exceeding 95% OPC purity. Differentiation was induced by transferring OPCs to neurosphere medium supplemented with 10% fetal bovine serum (FBS, Gibco) and 15 nM triiodothyronine (T3, Sigma–Aldrich). Cultures were maintained for 4–6 days under standard incubation conditions (37°C, 5% CO2), after which cells displayed characteristic mature OLs morphology, including MBP expression and extensive processes as assessed by IHC.

Oli‐neu cells were maintained in a defined culture medium consisting of DMEM/F‐12 supplemented with 5% FBS, 1% horse serum, 2% B27, 1% N2, 7.2 mM glucose (from Gibco or Sigma–Aldrich), and 1% penicillin‐streptomycin (Gibco), all maintained at 37°C in a humidified 5% CO2 incubator. Time‐dependent administration of pharmacological inhibitors (MCE), validated siRNA sequences (Sangon Biotech), and plasmid constructs (described below) were employed to dissect the molecular mechanisms governing OL differentiation and myelination processes. All reagents were pre‐optimized through dose‐response and time‐course experiments to ensure maximal efficacy without cytotoxicity.

Plasmid transfections were performed using Lipofectamine 3000 (Invitrogen) according to the manufacturer's optimized protocol. Full‐length cDNA sequences encoding PDK1, c‐Myc, and Ubiquitin were amplified from mouse spinal cord cDNA libraries via PCR, then subcloned into pLVX‐based expression vectors (pLVX‐HA, pLVX‐Flag, or pLVX‐GFP) for epitope tagging and functional overexpression studies. Constructs were sequence‐verified prior to use to ensure genetic fidelity. Gene knockdown experiments utilized siRNAs targeting *c‐Myc* (sequence: 5ʹ‐GCUUCGAAACUCUGGUGC‐3ʹ) and a scrambled non‐targeting control (Ribobio), delivered via Lipofectamine 3000 following manufacturer‐optimized transfection conditions. siRNA efficacy was confirmed through qRT‐PCR analysis of target gene expression 48 h post‐transfection.

Lentiviral transduction employed LV‐CRE‐IRES‐mCherry (BrainVTA) to achieve efficient Cre‐mediated recombination in OPCs, with parallel cultures infected with LV‐IRES‐mCherry serving as fluorescent controls. Viral stocks were diluted to a multiplicity of infection (MOI) of 20 and incubated for 48 h to enhance transduction efficiency. Fluorescent labeling was monitored 48 h post‐infection using flow cytometry to confirm >90% transduction efficiency prior to experimental use. All procedures adhere to MARC's biosafety guidelines to ensure reproducible results.

### 4.14 Stereotactic Injection

Stereotaxic delivery of rAAV virus (BrainVTA) into the ventrolateral region of the lumbar spinal cord (L4‐L6 segments) was performed on C57BL/6J mice at 1 month using a modified protocol based on previously published methods [56, 57]. All procedures were conducted under aseptic conditions with animals anesthetized via 2% isoflurane. To specifically modulate c‐Myc expression, control and *Pdk1* cKO mice were assigned to receive injections of either rAAV‐DIO‐EGFP (control virus) or rAAV‐DIO‐sh‐*Myc*‐EGFP (c‐Myc knockdown virus) through a stereotaxic frame specifically adapted for spinal cord surgeries. For cell‐type‐specific ablation studies, *NG2‐CreERT2* mice received injections of either rAAV‐DIO‐EGFP (control virus) or rAAV‐DIO‐taCaspase3‐EGFP (ablation virus) into the STT. Successful viral transduction and Cre‐mediated activation of taCaspase3 or sh‐*Myc* were induced by tamoxifen administration (i.p., 100 mg/kg, once daily for 3 consecutive days). Behavioral and histological assessments were performed four weeks after the completion of tamoxifen induction to allow for sufficient cell ablation or protein knockdown.

Before injection, the caudal thoracic region was shaved and disinfected with 70% ethanol. A midline skin incision was made to expose the vertebral column, followed by a precise laminectomy at L4‐L6 levels using microsurgical forceps under dissecting microscopy. Preoperative MRI imaging was used to confirm anatomical landmarks, ensuring targeted delivery into the spinal cord white matter. A glass micropipette (1 mm outer diameter) connected to a microinjection pump (World Precision Instruments) was inserted bilaterally 0.6 mm lateral to the midline and advanced to a depth of 1.1 mm from the dorsal surface. A total of 1 µL viral suspension was delivered at 0.25 µL/min over eight min using pressure‐controlled injection to minimize tissue displacement. Following injection, the needle was retracted over 5 min to allow viral solution diffusion, and the surgical site was closed with 5‐0 sterile nylon sutures (Ethicon) in a layered fashion. Animals were transferred to a 37°C warming platform for postoperative recovery. This standardized protocol ensured precise spatial targeting and controlled delivery kinetics while maintaining rigorous ethical standards in accordance with MARC guidelines for rodent surgeries.

### 4.15 Luciferase Assay

As recently reported [58], Oli‐neu cells were seeded in 96‐well plates at a density of 2×10^4^ cells/well and transfected using Lipofectamine 3000 (Invitrogen) following a lipid‐mediated transfection protocol. Cotransfection experiments employed plasmid combinations including c‐Myc expression constructs (NM_001177352.1) and *Sox10* promoter‐reporter plasmids (NM_011437.1), with all constructs sequence‐verified prior to use. The Dual‐Luciferase Reporter Assay System (Promega) was implemented to evaluate transcriptional regulation mechanisms, incorporating firefly luciferase as the experimental reporter and Renilla luciferase as an internal normalization control for transfection efficiency. Following 48‐h incubation under standard culture conditions (37°C, 5% CO2), cells were lysed using Passive Lysis Buffer. Firefly luciferase activity was first quantified using 1× Luciferase Assay Reagent II, followed by Renilla measurement with 1× Stop & Glo Reagent. The relative promoter activity was calculated as the Firefly/Renilla luminescence ratio to account for variations in transfection efficiency. All transfection experiments included empty vector controls and underwent triplicate technical replicates to ensure statistical robustness.

### 4.16 RNA‐seq

Transcriptome‐wide RNA‐seq analysis was performed on total RNA extracted from lumbar spinal cords of 2‐month‐old *Pdk1* cKO and control mice, as previously described [47]. RNA‐seq was carried out to profile global gene expression changes by SHBIO (Shanghai, China) on the Illumina platform. cDNA libraries were constructed using the VAHTS Universal V6 RNA‐seq Library Prep Kit following Illumina's standardized protocol, with library quality evaluated via Agilent 4200 Bioanalyzer analysis. Sequencing was conducted on an Illumina NovaSeq 6000 platform (paired‐end 150 bp reads) to generate ∼50 million reads per sample, ensuring sufficient coverage for reliable transcriptome profiling. Raw FASTQ files underwent a rigorous bioinformatics workflow: initial quality trimming was performed with Trimmomatic (v0.39) to remove adapter sequences and low‐quality bases (Q<20). Cleaned reads were mapped to the GRCm38.p4 (mm10) mouse reference genome using HISAT2 (v2.0.4) with default parameters, achieving >90% mapping efficiency. Transcript quantification was conducted via StringTie (v1.3.3b), followed by normalization using the trimmed mean of M‐values (TMM) method in edgeR (v3.32.1). Differentially expressed genes were identified through negative binomial modeling with strict criteria: false discovery rate (FDR) <0.05 and absolute log_2_ fold change ≥2.0 between experimental groups. This multi‐step filtering process incorporated both statistical significance and biological relevance thresholds to ensure robust identification of regulated genes. All computational analyses were performed in a blinded manner using standardized pipelines, with technical replicates (n = 3 per group) confirming reproducibility of expression profiles.

### 4.17 CO‐IP

IP and WB analyses were performed on Oli‐neu cells transfected with HA‐, Flag‐, or GFP‐tagged plasmids (sequence‐verified constructs). Cells were lysed in ice‐cold RIPA lysis buffer supplemented with 1% protease inhibitor cocktail to preserve protein integrity. For antibody incubation, protein extracts were rotated overnight at 4°C with validated primary antibodies: anti‐HA, anti‐Flag, or anti‐GFP. Immune complexes were captured using 30 µL Protein A/G Magnetic Beads (MCE) with 6‐h incubation at 4°C under constant rotation on an orbital shaker. Beads were washed three times with IP wash buffer to remove non‐specifically bound proteins. Target proteins were eluted by boiling in reducing SDS‐PAGE sample buffer at 95°C for 5 min to disrupt protein interactions while maintaining antigenicity. The eluted proteins were then subjected to WB analysis to detect the target proteins. All procedures incorporated blinded sample processing and technical replicates (n = 3) to ensure objective quantification of protein interactions.

### 4.18 Statistical Analysis

Statistical analyses were performed using GraphPad Prism 8.0 (GraphPad Software, Inc., La Jolla, CA, USA) following a rigorous statistical framework. Prior to statistical testing, data distributions were assessed for normality using the Shapiro‐Wilk test and for homogeneity of variance using Levene's test. No data transformation was required, and potential outliers were identified and excluded only if justified by Grubbs' test (*α* = 0.05). Quantitative data are presented as mean ± standard error of the mean (SEM) from at least three independent biological replicates per experimental condition. Unpaired t‐tests were applied for comparisons between two groups, while one‐way ANOVA and two‐way ANOVA were used for multiple group comparisons depending on experimental design complexity. Survival curves were analyzed using the Kaplan‐Meier method and the log‐rank (Mantel‐Cox) test. Statistical significance was denoted as *p< 0.05, **p< 0.01, ***p< 0.001, ****p< 0.0001, with “ns” indicating no significant difference (p ≥ 0.05).

## Author Contributions

Y.H., G.C., and J.Y. contributed to the conception of the study. P.Q., L.G., G.Y., C.H., and H.W. performed the experiments and analysed the results. Y.H., G.C., and J.Y. provided reagents. P.Q. and G.C. wrote the manuscript with inputs from all authors.

## Funding

This work was supported by the grant of Key Medical Projects from Jiangsu Provincial Health Commission (K2025040 to Y.H.) and grants from the National Natural Science Foundation of China (32270871 to G.C., U23A20421 to J.Y., and 82201330 to H.W.).

## Conflicts of Interest

The authors in this article declare no competing financial interests.

## Supporting information




**Supporting File 1**: advs75281‐sup‐0001‐SuppMat.docx.

**Supporting File 2**: advs75281‐sup‐0002‐Data.zip.

## Data Availability

The original RNA‐seq datasets generated in this study have been deposited in the National Center for Biotechnology Information (NCBI) Sequence Read Archive (SRA) under accession number PRJNA1309284. Source data for all figures and supplementary figures are provided with this paper. Any further requests for resources, reagents, or raw data should be directed to the lead contact, Prof. Guiquan Chen (chenguiquan@nju.edu.cn).

## References

[1] T. S. Jensen , R. Baron , M. Haanp , et al., “A New Definition of Neuropathic Pain,” Pain 152 (2011): 2204–2205, 10.1016/j.pain.2011.06.017. 21764514

[2] X. Moisset , “Neuropathic Pain: Evidence Based Recommendations,” Presse Médicale 53 (2024): 104232. 38641202 10.1016/j.lpm.2024.104232

[3] L. A. Osso and E. G. Hughes , “Dynamics of Mature Myelin,” Nature Neuroscience 27 (2024): 1449–1461, 10.1038/s41593-024-01642-2. 38773349 PMC11515933

[4] I. L. Arancibia‐Cárcamo , M. C. Ford , L. Cossell , K. Ishida , K. Tohyama , and D. Attwell , “Node of Ranvier Length as a Potential Regulator of Myelinated Axon Conduction Speed,” Elife 6 (2017): 23329, 10.7554/eLife.23329. PMC5313058 28130923

[5] W. Kim and M. C. Angulo , “Unraveling the Role of Oligodendrocytes and Myelin in Pain,” Journal of Neurochemistry 169 (2024): 16206. 10.1111/jnc.16206 PMC11657919 39162089

[6] X. Q. Wang , C. M. Lo , L. Chen , E. S.‐W. Ngan , A. Xu , and R. Y. Poon , “CDK1‐PDK1‐PI3K/Akt Signaling Pathway Regulates Embryonic and Induced Pluripotency,” Cell Death & Differentiation 24 (2016): 38–48, 10.1038/cdd.2016.84. 27636107 PMC5260505

[7] Y. Wei , X. Han , and C. Zhao , “PDK1 Regulates the Survival of the Developing Cortical Interneurons,” Molecular Brain 13 (2020): 65, 10.1186/s13041-020-00604-6. 32366272 PMC7197138

[8] X. Han , Y. Wei , R. Ba , L. Sun , and C. Zhao , “PDK1 Regulates the Lengthening of G1 Phase to Balance RGC Proliferation and Differentiation During Cortical Neurogenesis,” Cerebral Cortex 32 (2022): 3488–3500, 10.1093/cercor/bhab428. 34918060

[9] R. Vaz , J. Wincent , N. Elfissi , et al., “A Missense Variant in PDK1 Associated With Severe Neurodevelopmental Delay and Epilepsy,” Biomedicines 10 (2022): 3171, 10.3390/biomedicines10123171. 36551928 PMC9775741

[10] C. Xu , L. Yu , J. Hou , et al., “Conditional Deletion of PDK1 in the Forebrain Causes Neuron Loss and Increased Apoptosis During Cortical Development,” Frontiers in Cellular Neuroscience 11 (2017): 330, 10.3389/fncel.2017.00330. 29104535 PMC5655024

[11] R. Liu , M. Xu , X.‐Y. Zhang , et al., “PDK1 Regulates the Maintenance of Cell Body and the Development of Dendrites of Purkinje Cells by pS6 and PKCγ,” The Journal of Neuroscience 40 (2020): 5531–5548, 10.1523/JNEUROSCI.2496-19.2020. 32487697 PMC7363466

[12] L. Cordón‐Barris , S. Pascual‐Guiral , S. Yang , et al., “Mutation of the 3‐Phosphoinositide‐Dependent Protein Kinase 1 (PDK1) Substrate‐Docking Site in the Developing Brain Causes Microcephaly With Abnormal Brain Morphogenesis Independently of Akt, Leading to Impaired Cognition and Disruptive Behaviors,” Molecular and Cellular Biology 36 (2023): 2967–2982, 10.1128/MCB.00230-16. PMC5108884 27644329

[13] G. J. Bennett and Y. K. Xie , “A Peripheral Mononeuropathy in Rat that Produces Disorders of Pain Sensation Like Those Seen in Man,” Pain 33 (1988): 87–107, 10.1016/0304-3959(88)90209-6. 2837713

[14] J. Hou , H. Bi , Z. Ye , et al., “Pen‐2 Negatively Regulates the Differentiation of Oligodendrocyte Precursor Cells Into Astrocytes in the Central Nervous System,” The Journal of Neuroscience 41 (2021): 4976–4990, 10.1523/JNEUROSCI.2455-19.2021. 33972402 PMC8197633

[15] J. Chunxia , W. Qiu , Y. Yang , et al., “ADAMTS4 Enhances Oligodendrocyte Differentiation and Remyelination by Cleaving NG_2_ Proteoglycan and Attenuating PDGFRα Signaling,” Journal of Neuroscience 43 (2023): 4405–4417. 37188512 10.1523/JNEUROSCI.2146-22.2023 PMC10278682

[16] I. Malta , T. Moraes , G. Rodrigues , P. Franco , and G. Galdino , “The Role of Oligodendrocytes in Chronic Pain: Cellular and Molecular Mechanisms,” Journal of Physiology and Pharmacology 70 (2019): 299–309. 10.26402/jpp.2019.5.02 31889038

[17] S. Nishimoto , K. Okada , H. Tanaka , et al., “Neurotropin Attenuates Local Inflammatory Response and Inhibits Demyelination Induced by Chronic Constriction Injury of the Mouse Sciatic Nerve,” Biologicals 44 (2016): 206–211, 10.1016/j.biologicals.2016.03.005. 27233579

[18] H. Wang , M. Liu , G. Zou , et al., “Deletion of PDK1 in Oligodendrocyte Lineage Cells Causes White Matter Abnormality and Myelination Defect in the Central Nervous System,” Neurobiology of Disease 148 (2021): 105212, 10.1016/j.nbd.2020.105212. 33276084

[19] W. Huang , N. Zhao , X. Bai , et al., “Novel NG2‐CreERT2 Knock‐in Mice Demonstrate Heterogeneous Differentiation Potential of NG2 glia During Development,” Glia 62 (2014): 896–913, 10.1002/glia.22648. 24578301

[20] V. Llombart and M. R. Mansour , “Therapeutic Targeting of “"Undruggable”" MYC,” EBioMedicine 75 (2022): 103756. 34942444 10.1016/j.ebiom.2021.103756 PMC8713111

[21] L. Magri , M. Gacias , M. Wu , V. A. Swiss , W. G. Janssen , and P. Casaccia , “c‐Myc‐Dependent Transcriptional Regulation of Cell Cycle and Nucleosomal Histones During Oligodendrocyte Differentiation,” Neuroscience 276 (2014): 72–86, 10.1016/j.neuroscience.2014.01.051. 24502923 PMC4294794

[22] Y. Ding , Q. Xiang , P. Zhu , et al., “Qihuang Zhuyu Formula Alleviates Coronary Microthrombosis by Inhibiting PI3K/Akt/αIIbβ3‐Mediated Platelet Activation,” Phytomedicine 125 (2024): 155276, 10.1016/j.phymed.2023.155276. 38295661

[23] G. Cruccu , M. J. Aminoff , G. Curio , et al., “Recommendations for the Clinical Use of Somatosensory‐Evoked Potentials,” Clinical Neurophysiology 119 (2008): 1705–1719, 10.1016/j.clinph.2008.03.016. 18486546

[24] S. Ghorbanpoor , L. M. Garcia‐Segura , A. Haeri‐Rohani , F. Khodagholi , and M. Jorjani , “Aromatase Inhibition Exacerbates Pain and Reactive Gliosis in the Dorsal Horn of the Spinal Cord of Female Rats Caused by Spinothalamic Tract Injury,” Endocrinology 155 (2014): 4341–4355, 10.1210/en.2014-1158. 25105782

[25] H. H. Jung , C. S. Koh , M. Park , et al., “Microglial Deactivation by Adeno‐Associated Virus Expressing Small‐Hairpin GCH1 has Protective Effects Against Neuropathic Pain Development in a Spinothalamic Tract‐Lesion Model,” CNS neuroscience & therapeutics 28 (2022): 36–45, 10.1111/cns.13751. 34845843 PMC8673712

[26] G. Wang and S. M. Thompson , “Maladaptive Homeostatic Plasticity in a Rodent Model of Central Pain Syndrome: Thalamic Hyperexcitability After Spinothalamic Tract Lesions,” The Journal of Neuroscience 28 (2008): 11959–11969, 10.1523/JNEUROSCI.3296-08.2008. 19005061 PMC2627563

[27] F. Mei , S. P. J. Fancy , Y.‐A. A. Shen , et al., “Micropillar Arrays as a High‐Throughput Screening Platform for Therapeutics in Multiple Sclerosis,” Nature Medicine 20 (2014): 954–960, 10.1038/nm.3618. PMC4830134 24997607

[28] R. R. Ji , A. Nackley , Y. Huh , N. Terrando , and W. Maixner , “Neuroinflammation and Central Sensitization in Chronic and Widespread Pain,” Anesthesiology 129 (2018): 343–366, 10.1097/ALN.0000000000002130. 29462012 PMC6051899

[29] C. R. Donnelly , A. S. Andriessen , G. Chen , et al., “Central Nervous System Targets: Glial Cell Mechanisms in Chronic Pain,” Neurotherapeutics 17 (2020): 846–860, 10.1007/s13311-020-00905-7. 32820378 PMC7609632

[30] E. López‐Muguruza , C. Peiró‐Moreno , F. Pérez‐Cerdá , C. Matute , and A. Ruiz , “Del Río Hortega's Insights Into Oligodendrocytes: Recent Advances in Subtype Characterization and Functional Roles in Axonal Support and Disease,” Frontiers in Neuroanatomy 19 (2025): 1557214, 10.3389/fnana.2025.1557214. 40145026 PMC11936973

[31] D. Li , K. Yang , J. Li , et al., “Single‐Cell Sequencing Reveals Glial Cell Involvement in Development of Neuropathic Pain Via Myelin Sheath Lesion Formation in the Spinal Cord,” Journal of Neuroinflammation 21 (2024): 213, 10.1186/s12974-024-03207-3. 39217340 PMC11365210

[32] V. Tepavcevic and C. Lubetzki , “Oligodendrocyte Progenitor Cell Recruitment and Remyelination in Multiple Sclerosis: The More, the Merrier?,” Brain 145 (2022): 4178–4192, 10.1093/brain/awac307. 36093726

[33] S. A. Freeman , A. Desmazieres , J. Simonnet , et al., “Acceleration of Conduction Velocity Linked to Clustering of nodal components precedes myelination,” Proceedings of the National Academy of Sciences of the United States of America 112 (2015): E321–E328. 25561543 10.1073/pnas.1419099112 PMC4311839

[34] R. E. Pepper , K. A. Pitman , C. L. Cullen , and K. M. Young , “How Do Cells of the Oligodendrocyte Lineage Affect Neuronal Circuits to Influence Motor Function, Memory and Mood?,” Frontiers in Cellular Neuroscience 12 (2018): 399, 10.3389/fncel.2018.00399. 30524235 PMC6262292

[35] B. Brites , D. Fernandes , and A. Fernandes , “Oligodendrocyte Development and Myelination in Neurodevelopment: Molecular Mechanisms in Health and Disease,” Current pharmaceutical design 22 (2016): 656–679. 26635271 10.2174/1381612822666151204000636

[36] S. Gritsch , J. Lu , S. Thilemann , et al., “Oligodendrocyte Ablation Triggers Central Pain Independently of Innate or Adaptive Immune Responses in Mice,” Nature communications 5 (2014): 5472, 10.1038/ncomms6472. PMC4268702 25434649

[37] C. Zhao , Z. Yang , J. Gao , et al., “PDK1 Regulates Transition Period of Apical Progenitors to Basal Progenitors by Controlling Asymmetric Cell Division,” Cerebral cortex 30 (2020): 406–420. 31504280 10.1093/cercor/bhz146

[38] S. G. Waxman , “Axonal Conduction and Injury in Multiple Sclerosis: The Role of Sodium Channels,” Nature Reviews Neuroscience 7 (2006): 932–941, 10.1038/nrn2023. 17115075

[39] L.‐E. Lorenzo , A. G. Godin , F. Ferrini , et al., “Enhancing Neuronal Chloride Extrusion Rescues α2/α3 GABAA‐Mediated Analgesia in Neuropathic Pain,” Nature Communications 11 (2020): 869, 10.1038/s41467-019-14154-6. PMC7018745 32054836

[40] N. Yousefpour , S. N. Tansley , S. Locke , et al., “Targeting C1q Prevents Microglia‐Mediated Synaptic Removal in Neuropathic Pain,” Nature Communications 16 (2025): 4590, 10.1038/s41467-025-59849-1. PMC12085617 40382320

[41] R. A. G. Khammissa , R. Ballyram , J. Fourie , M. Bouckaert , J. Lemmer , and L. Feller , “Selected Pathobiological Features and Principles of Pharmacological Pain Management,” Journal of International Medical Research 48 (2020): 300060520903653, 10.1177/0300060520903653. 32408839 PMC7232056

[42] M. Cantone , M. Küspert , S. Reiprich , et al., “A Gene Regulatory Architecture that Controls Region‐Independent Dynamics of Oligodendrocyte Differentiation,” Glia 67 (2019): 825–843, 10.1002/glia.23569. 30730593

[43] B. A. Barres , J. Hornig , F. Fröb , et al., “The Transcription Factors Sox10 and Myrf Define an Essential Regulatory Network Module in Differentiating Oligodendrocytes,” PLoS Genetics 9 (2013): 1003907. 10.1371/journal.pgen.1003907 PMC3814293 24204311

[44] N. Li , M. Yao , J. Liu , et al., “Vitamin D Promotes Remyelination by Suppressing c‐Myc and Inducing Oligodendrocyte Precursor Cell Differentiation After Traumatic Spinal Cord Injury,” International Journal of Biological Sciences 18 (2022): 5391–5404, 10.7150/ijbs.73673. 36147469 PMC9461656

[45] E. G. Hughes , S. H. Kang , M. Fukaya , and D. E. Bergles , “Oligodendrocyte Progenitors Balance Growth With Self‐Repulsion to Achieve Homeostasis in the Adult Brain,” Nature Neuroscience 16 (2013): 668–676, 10.1038/nn.3390. 23624515 PMC3807738

[46] M. Liu , Y. Zhang , X. Y. Teng , et al., “Phosphatase PP2A is Required for CNS Myelination Via Proteasome‐Dependent Regulation of Sox10 Expression,” Glia 74 (2025): 70082. 10.1002/glia.70082 40931903

[47] X.‐Y. Teng , P. Hu , C.‐M. Zhang , et al., “OPALIN is an LGI1 Receptor Promoting Oligodendrocyte Differentiation,” Proceedings of the National Academy of Sciences of the United States of America 121 (2024): 2403652121, 10.1073/pnas.2403652121. PMC11317624 39083419

[48] R. Feng , H. Wang , J. Wang , D. Shrom , X. Zeng , and J. Z. Tsien , “Forebrain Degeneration and Ventricle Enlargement Caused by Double Knockout of Alzheimer's Presenilin‐1 and Presenilin‐2,” Proceedings of the National Academy of Science of the United States of America 101 (2004): 8162–8167, 10.1073/pnas.0402733101. PMC419574 15148382

[49] M. D. Muzumdar , B. Tasic , K. Miyamichi , L. Li , and L. Luo , “A Global Double‐Fluorescent Cre Reporter Mouse,” Genesis 45 (2007): 593–605. 17868096 10.1002/dvg.20335

[50] M. Ou , Y. Chen , J. Liu , et al., “Spinal Astrocytic MeCP2 Regulates Kir4.1 for the Maintenance of Chronic Hyperalgesia in Neuropathic Pain,” Progress in Neurobiology 224 (2023): 102436, 10.1016/j.pneurobio.2023.102436. 36931588 PMC10372923

[51] L.‐W. Chu , K.‐I. Cheng , J.‐Y. Chen , et al., “Loganin Prevents Chronic Constriction Injury‐Provoked Neuropathic Pain by Reducing TNF‐α/IL‐1β‐Mediated NF‐κB Activation and Schwann Cell Demyelination,” Phytomedicine 67 (2020): 153166, 10.1016/j.phymed.2019.153166. 31955133

[52] W. J. Dixon , “Efficient Analysis of Experimental Observations,” Annual Review of Pharmacology and Toxicology 20 (1980): 441–462. 10.1146/annurev.pa.20.040180.002301 7387124

[53] T. Akiyama , T. Nguyen , E. Curtis , et al., “A Central Role for Spinal Dorsal Horn Neurons that Express Neurokinin‐1 Receptors in Chronic Itch,” Pain 156 (2015): 1240–1246, 10.1097/j.pain.0000000000000172. 25830923 PMC4474752

[54] R. Z. Hill , M. C. Loud , A. E. Dubin , B. Peet , and A. Patapoutian , “PIEZO1 Transduces Mechanical Itch in Mice,” Nature 607 (2022): 104–110, 10.1038/s41586-022-04860-5. 35732741 PMC9259491

[55] X.‐J. Kuang , C.‐Y. Zhang , B.‐Y. Yan , et al., “P2X2 Receptors in Pyramidal Neurons are Critical for Regulating Vulnerability to Chronic Stress,” Theranostics 12 (2022): 3703–3718, 10.7150/thno.72144. 35664080 PMC9131261

[56] P. Inquimbert , M. Moll , T. Kohno , and J. Scholz , “Stereotaxic Injection of a Viral Vector for Conditional Gene Manipulation in the Mouse Spinal Cord,” Journal of Visualized Experiments: JoVE 18 (2013): 50313. 10.3791/50313 PMC3639548 23542888

[57] Z. Gu , F. Li , Y. P. Zhang , et al., “Apolipoprotein E Mimetic Promotes Functional and Histological Recovery in Lysolecithin‐Induced Spinal Cord Demyelination in Mice,” Journal of Neurology & Neurophysiology Suppl 12 (2014): 10. 10.4172/2155-9562.S12-010 PMC4309015 25642353

[58] Y. Chai , H. Xiang , Y. Ma , et al., “S1PR1 Suppresses Lung Adenocarcinoma Progression Through p‐STAT1/miR‐30c‐5 p/FOXA1 Pathway,” Journal of Experimental & Clinical Cancer Research 43 (2024): 304, 10.1186/s13046-024-03230-5. 39551792 PMC11571582

