# Supplementary material for: Targeting the PDK1/c‐Myc/SOX10 Signaling in Oligodendrocytes Alleviates Neuropathic Pain
Source: Adv Sci (Weinh). 2026 Apr 16;13(39):e16426. doi: 10.1002/advs.202516426 (PMC13334879; doi:10.1002/advs.202516426)
Supplement: Supplementary file 1 — Supporting File 1: advs75281‐sup‐0001‐SuppMat.docx. [file ADVS-13-e16426-s001.docx]

**Supporting information**

**Title: Targeting the PDK1/c-Myc/SOX10 Signaling in Oligodendrocytes Alleviates Neuropathic Pain**

**Pingping Qiao^1,2^, Lifang Guo^3^, Guochao Yang^1^, Chaoli Huang^4^, He Wang^5^, Jianjun Yang^5,*^, Guiquan Chen^1,2,*^,** **Yimin Hu^6,*^**

* Corresponding authors.

E-mail addresses:

guyueym@pumcderm.cams.cn (Y.H.), chenguiquan@nju.edu.cn (G.C.), or yjyangjj@126.com (J.Y.).

**List of Supporting Information:**

**Supplementary Figure S1 to S9**

**Table S1 to S4**


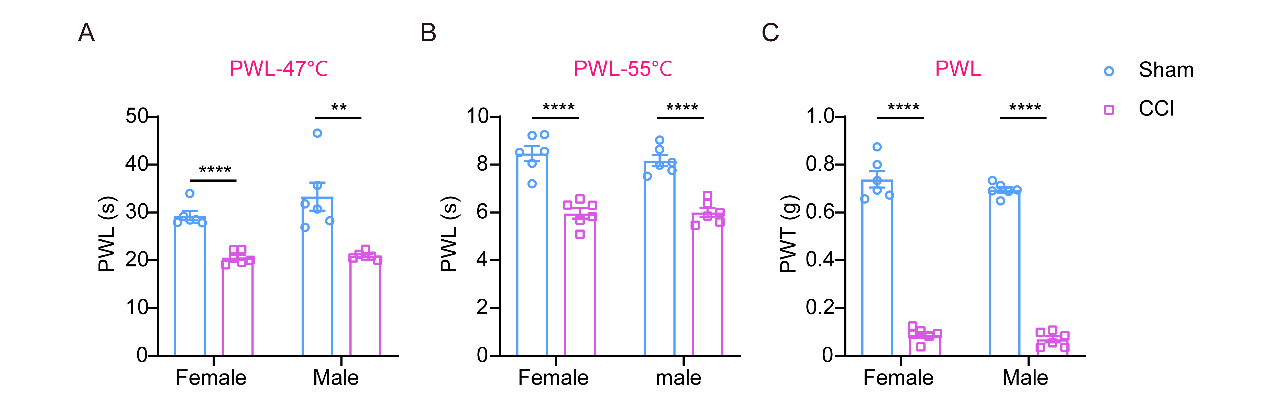


**Figure S1. Male and female mice exhibited similar nociceptive responses following CCI. A-C**. Comparison of CCI-induced pain sensitivity between male and female mice. No significant differences were observed between male and female mice in either mechanical (**A**, **B**) or thermal (**C**) pain sensitivity at day 14 post-CCI injury (unpaired t test; mean ± SEM; n = 6 mice per group). **p*< 0.05; ***p*< 0.01; ****p*< 0.001; *****p*< 0.0001; ns, no significant difference.


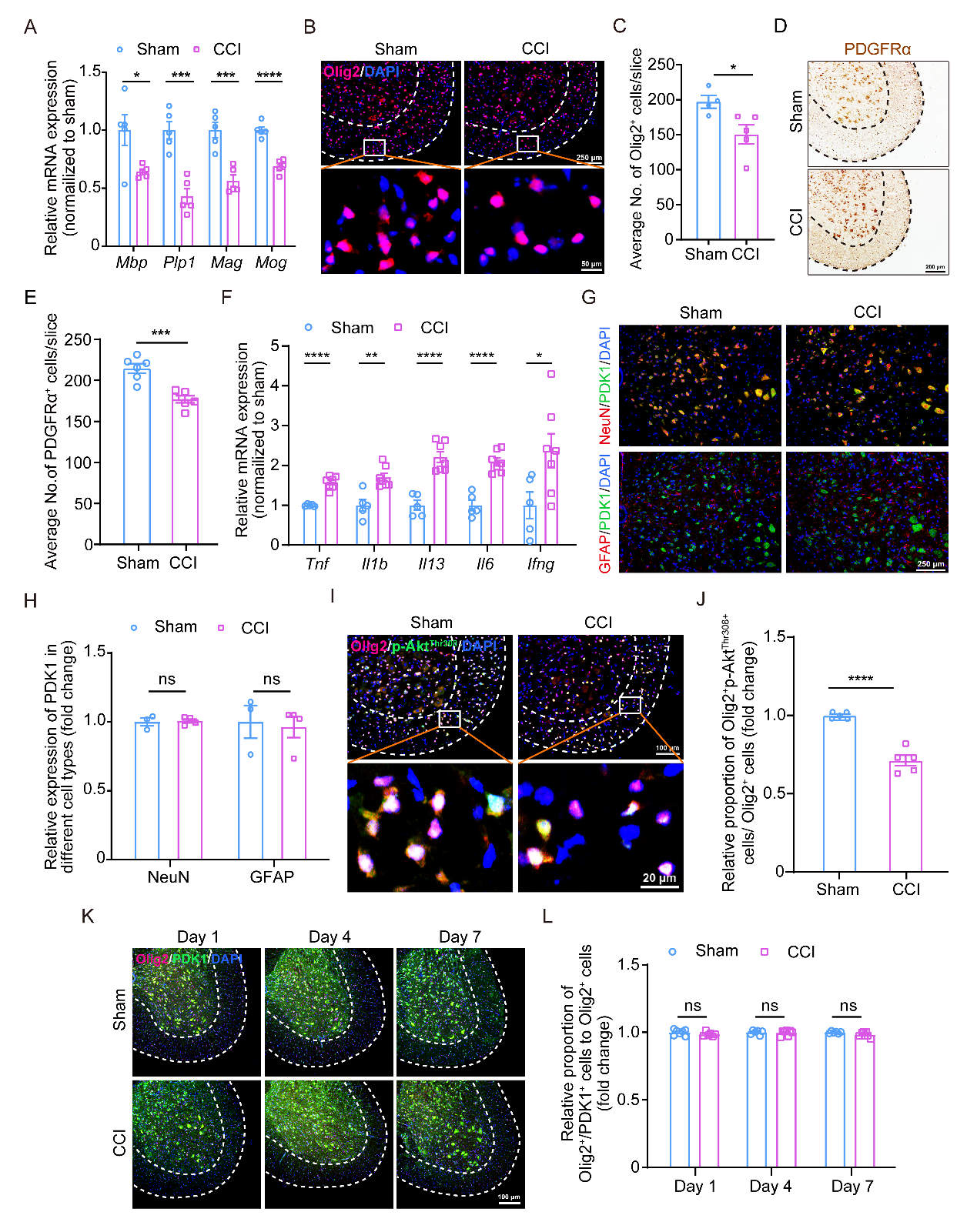


**Figure S2. CCI suppressed myelin gene expression and OL lineage cell numbers without altering PDK1 levels in spinal cord neurons and astrocytes. A**. qRT-PCR analysis of *Mbp*, *Plp1*, *Mag*, and *Mog* in the ipsilateral spinal cords. Quantification of qRT-PCR demonstrated reduced mRNA levels of key myelin-related genes, such as *Mbp*, *Plp1*, *Mag*, and *Mog*, in the ipsilateral spinal cords post-CCI injury (unpaired t test; mean ± SEM; n ≥ 4 mice per group). **B**. Representative IHC images of Olig2 (red) in the ipsilateral spinal cords. Scale bar = 250 and 50 μm. **C**. Quantification of Olig2^+^ cells. IHC staining of Olig2 showed a significantly reduced number of OL lineage cells in the ipsilateral spinal cord's ventral and lateral white matter versus sham controls (unpaired t test; mean ± SEM; n ≥ 4 mice per group). **D**. Representative IHC images showing PDGFRα⁺ cells of sham and CCI mice. Scale bar = 250 μm. **E**. Quantification of PDGFRα⁺ cell densities in the white matter of spinal cords. The population of PDGFRα⁺ cells was significantly reduced in the ipsilateral CCI mice. (unpaired t test; mean ± SEM; n = 6 mice per group). **F**. qRT-PCR analysis of pro-inflammatory cytokine in the spinal cords. qRT-PCR quantification revealed significantly increased mRNA levels of *Tnf*, *Il1b*, *Il13*, *Il6*, and *Ifng* in the ipsilateral spinal cords following CCI injury (unpaired t test; mean ± SEM; n ≥ 5 mice per group). **G**. Representative images for the co-staining of NeuN (red) and PDK1 (green) or GFAP (red) and PDK1 (green). Scale bar = 250 μm. **H**. Quantification of the ratio of NeuN^+^PDK1^+^/ NeuN^+^ cell numbers or the ratio of GFAP^+^PDK1^+^/ GFAP^+^ cell numbers. IHC staining showed no significant differences in PDK1 expression levels in these two cell types between sham and CCI-injured mouse spinal cords (unpaired t test; mean ± SEM; n ≥ 3 mice per group). **I.** Representative IHC images of Olig2 (red) and p-Akt^Thr308^ (green) in the ipsilateral spinal cord's ventral and lateral white matter. Scale bar = 100 and 20 μm. **J**. Quantification of Olig2^+^p-Akt^Thr308+^/ Olig2^+^ numbers normalized to sham groups in the white matter. The percentage of Olig2^+^p-Akt^Thr308+^/ Olig2^+^ cell numbers was significantly decreased in the ventral and lateral white matter of the spinal cords after CCI injury (unpaired t test; mean ± SEM; n = 5 mice per group). **K**, **L**. Representative IHC images (**K**) and quantification (**L**) showing PDK1 (green) expression in Olig2⁺ (red) OL-lineage cells in the spinal cords of sham and CCI mice at day 1, day 4, and day 7 post-surgery. PDK1 levels remained unchanged in ventral and lateral white matter Olig2⁺ cells during the early stages following CCI (unpaired t test; mean ± SEM; n ≥ 6 mice per group). Scale bars = 100 μm. **p*< 0.05; ***p*< 0.01; ****p*< 0.001; *****p*< 0.0001; ns, no significant difference.


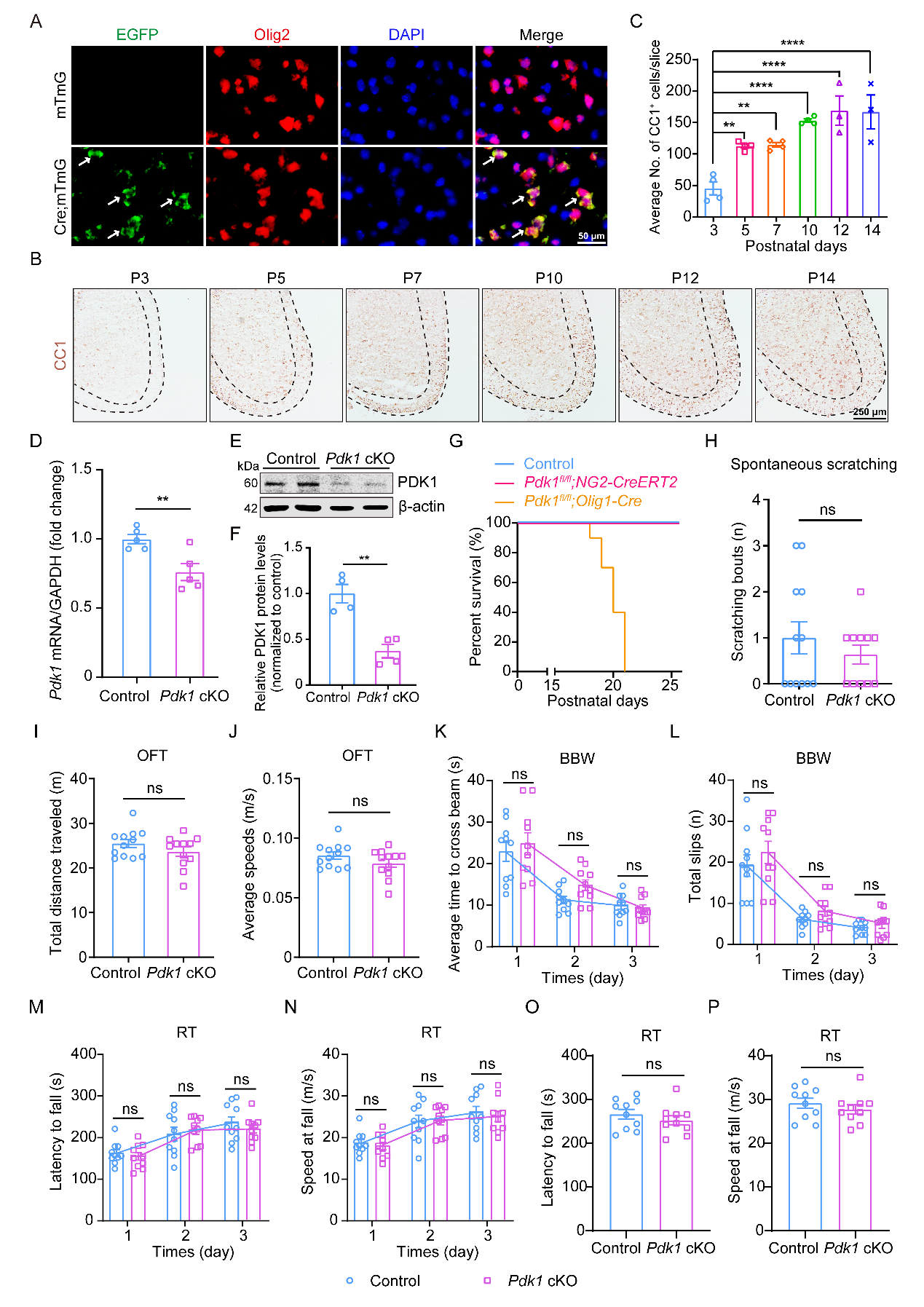


**Figure S3. *NG2-CreERT2*-mediated *Pdk1* deletion in OL lineage cells did not induce itch behaviors or motor deficits in mice. A**. *NG2-CreERT2*-mediated EGFP expression. Only in the presence of Cre, EGFP (green) was expressed upon tamoxifen induction and colocalized with Olig2 (red). Scale bar = 50 μm. **B**. Representative IHC images showing CC1 staining in mouse spinal cords. Scale bar = 250 μm. **C**. Quantification of CC1⁺ matured OLs at different postnatal time points. The results indicate that P3-5 constitute a critical period for OL differentiation and maturation, and the number of CC1⁺ cells reaches a stable level by P10 (one-way ANOVA; mean ± SEM; n ≥ 3 mice per group). **D**. qRT-PCR analysis of *Pdk1* showed a significantly decreased expression in *Pdk1* cKO spinal cords (unpaired t test; mean ± SEM; n = 5 mice per group). **E**. Represent WB images of PDK1 and β-actin. **F**. Quantification of PDK1 protein levels normalized to β-actin. WB analysis showed that the protein levels of PDK1 were remarkably decreased in the spinal cords of *Pdk1* cKO mice (unpaired t test; mean ± SEM; n = 4 mice per group). **G**. Percent survival rate of mice. All *Olig1-Cre*-mediated *Pdk1* deletion mice died before P21 (n = 10 mice per group). **H**. Measurement of spontaneous scratching in control and *Pdk1* cKO mice. Both genotypes showed minimal spontaneous scratching during the 30-minute observation period (unpaired t test; mean ± SEM; n ≥ 11 mice per group). **I, J**. OFT analysis of control and *Pdk1* cKO mice. There were no significant differences in the total distance traveled (**I**) or the average speeds (**J**) between the two groups (unpaired t test; mean ± SEM; n = 10 mice per group). **K, L**. Statistical analysis of  *Pdk1* cKO and control mice in the BBW task. The average time to cross the beam (**K**) and the number of total slips (**L**) were comparable between control and *Pdk1* cKO mice across the three training days (unpaired t test; mean ± SEM; n = 10 mice per group). **M-P**. RT performance of control and *Pdk1* cKO mice. Animals were trained for three consecutive days (**M**, **N**) and tested on the fourth day (**O**, **P**). No significant differences were observed in the latency to fall (**M**, **O**) or the speeds at fall (**N**, **P**) between the two groups during either the training or testing phases (unpaired t test; mean ± SEM; n = 10 mice per group). **p*< 0.05; ***p*< 0.01; ****p*< 0.001; *****p*< 0.0001; ns, no significant difference.


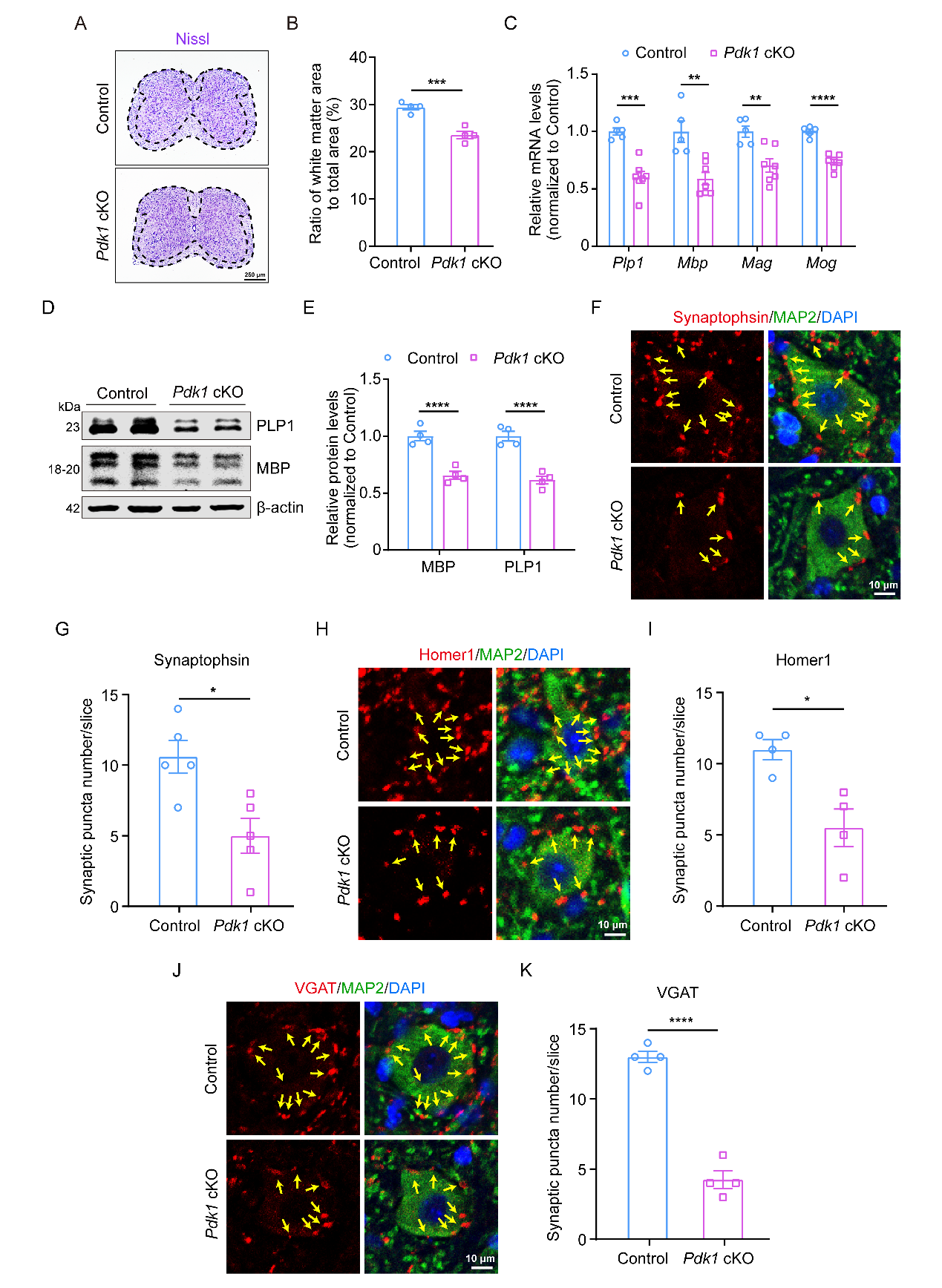


**Figure S4. Oligodendrocytic *Pdk1* deficiency impaired white matter structural integrity and myelination. A**. Represent images of Nissl staining. Scale bar = 250 μm. **B**. Quantification of the ratio of white matter/intact spinal cord. Nissl staining showed a significant decrease in the proportion of *Pdk1* cKO spinal cord white matter area compared to that in the control (unpaired t test; mean ± SEM; n ≥ 4 mice per group). **C**. Relative mRNA levels of *Plp1*, *Mbp*, *Mag*, and *Mog* were remarkably decreased in the *Pdk1* cKO spinal cords (unpaired t test; mean ± SEM; n ≥ 5 mice per group). **D, E**. Representative WB images (**D**) and quantification (**E**) of MBP and PLP1 in the spinal cords of control and *Pdk1* cKO mice. WB analysis of MBP and PLP1 both showed an obvious decrease in protein expression in the *Pdk1* cKO spinal cords (unpaired t test; mean ± SEM; n = 4 mice per group). **F-I**. Representative IHC images of Synaptophysin (red, **F**) and Homer1 (red, **H**) co-stained with MAP2 (green) and DAPI (blue) in the spinal cords of control and *Pdk1* cKO mice. Quantification revealed a significant reduction in the number of Synaptophysin^+^ (**G**) and Homer1^+^ (**I**) synaptic puncta in *Pdk1* cKO mice compared with controls (unpaired t test; mean ± SEM; n = 4 mice per group). Scale bar = 10 μm. **J**. Representative IHC images of VGAT (red) co-stained with MAP2 (green) and DAPI (blue) in the spinal cords of control and *Pdk1* cKO mice. Yellow arrows indicate VGAT^+^ inhibitory synaptic puncta. Scale bar = 10 μm. **K**. Quantification revealed a significant reduction in the number of VGAT^+^ synaptic puncta in *Pdk1* cKO mice compared with controls (unpaired t test; mean ± SEM; n = 4 mice per group). **p*< 0.05; ***p*< 0.01; ****p*< 0.001; *****p*< 0.0001; ns, no significant difference.


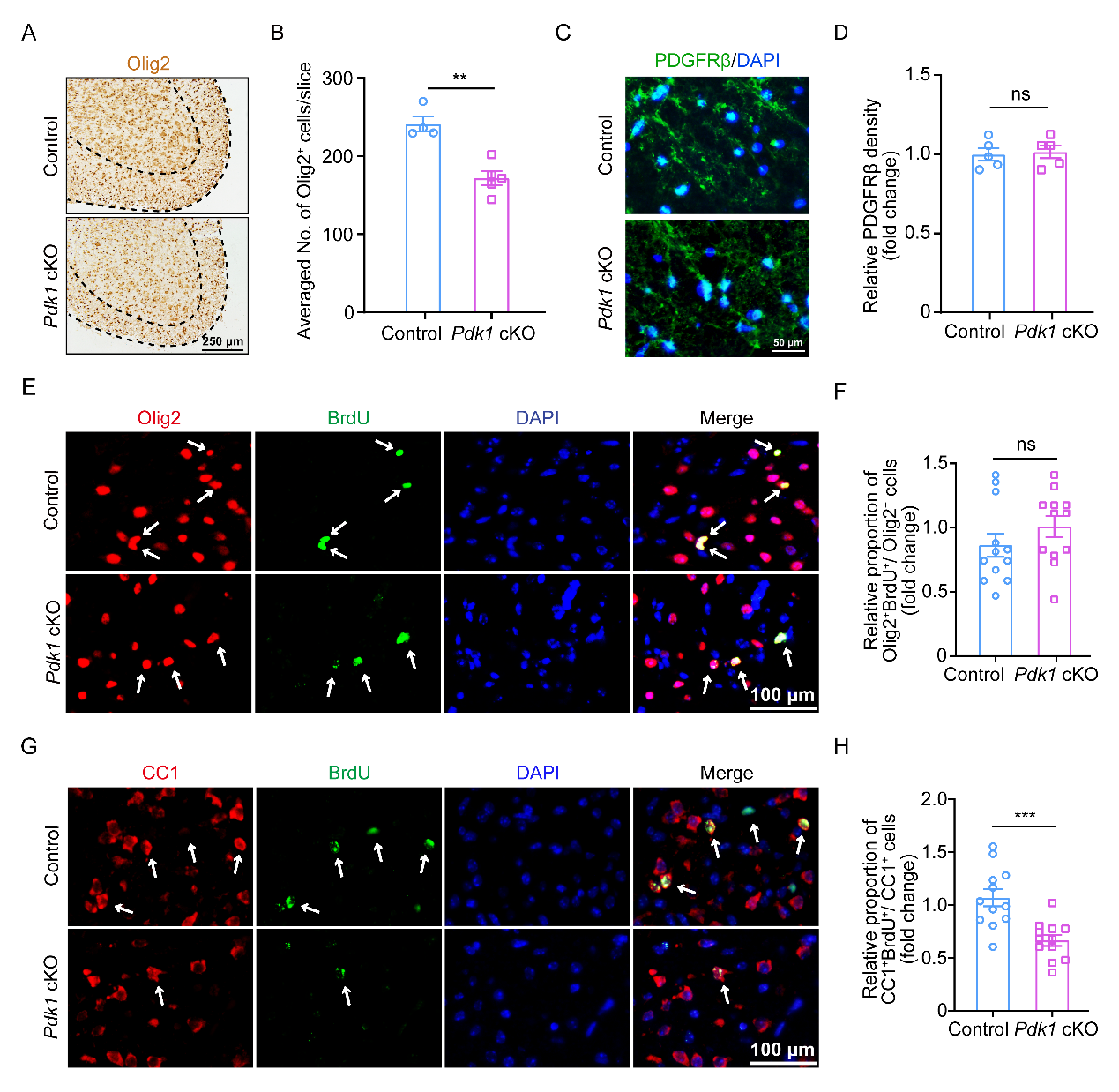


**Figure S5. Loss of PDK1 in OL lineage cells impaired OL differentiation. A, B**. Representative IHC images (**A**) and quantification (**B**) of averaged Olig2^+^ cells in the spinal cords of control and *Pdk1* cKO mice. IHC results showed a significant loss of Olig2^+^ cells in *Pdk1* cKO spinal cords (unpaired t test; mean ± SEM; n ≥ 4 mice per group). Scale bar = 250 μm. **C**. Representative immunofluorescence images of PDGFRβ staining in the spinal cords of control and *Pdk1* cKO mice. Scale bar = 50 μm. **D**. Quantification of relative PDGFRβ signal density in spinal white matter. There was no significant difference between groups (unpaired t test; mean ± SEM; n = 5 mice per group). **E**. Representative IHC images of Olig2 (red) and BrdU (green). Scale bar = 100 μm. **F**. Quantification of the ratio of Olig2^+^BrdU^+^/ Olig2^+^ cell numbers in the spinal cord's white matter. There was no statistical difference in the ratio of Olig2^+^BrdU^+^/ Olig2^+^ cells between the control and *Pdk1* cKO mice (unpaired t test; mean ± SEM; n ≥ 6 mice per group). **G**. Representative IHC images of CC1 (red) and BrdU (green) labeling in the spinal cords. Scale bar = 100 μm. **H**. Quantification of the proportion of CC1^+^Brdu^+^/ CC1^+^ cell numbers in the white matter of the spinal cords. The percentage of CC1^+^BrdU^+^ cells relative to CC1^+^ cells was significantly lower in the *Pdk1* cKO mice (unpaired t test; mean ± SEM; n ≥ 6 mice per group). **p*< 0.05; ***p*< 0.01; ****p*< 0.001; *****p*< 0.0001; ns, no significant difference.


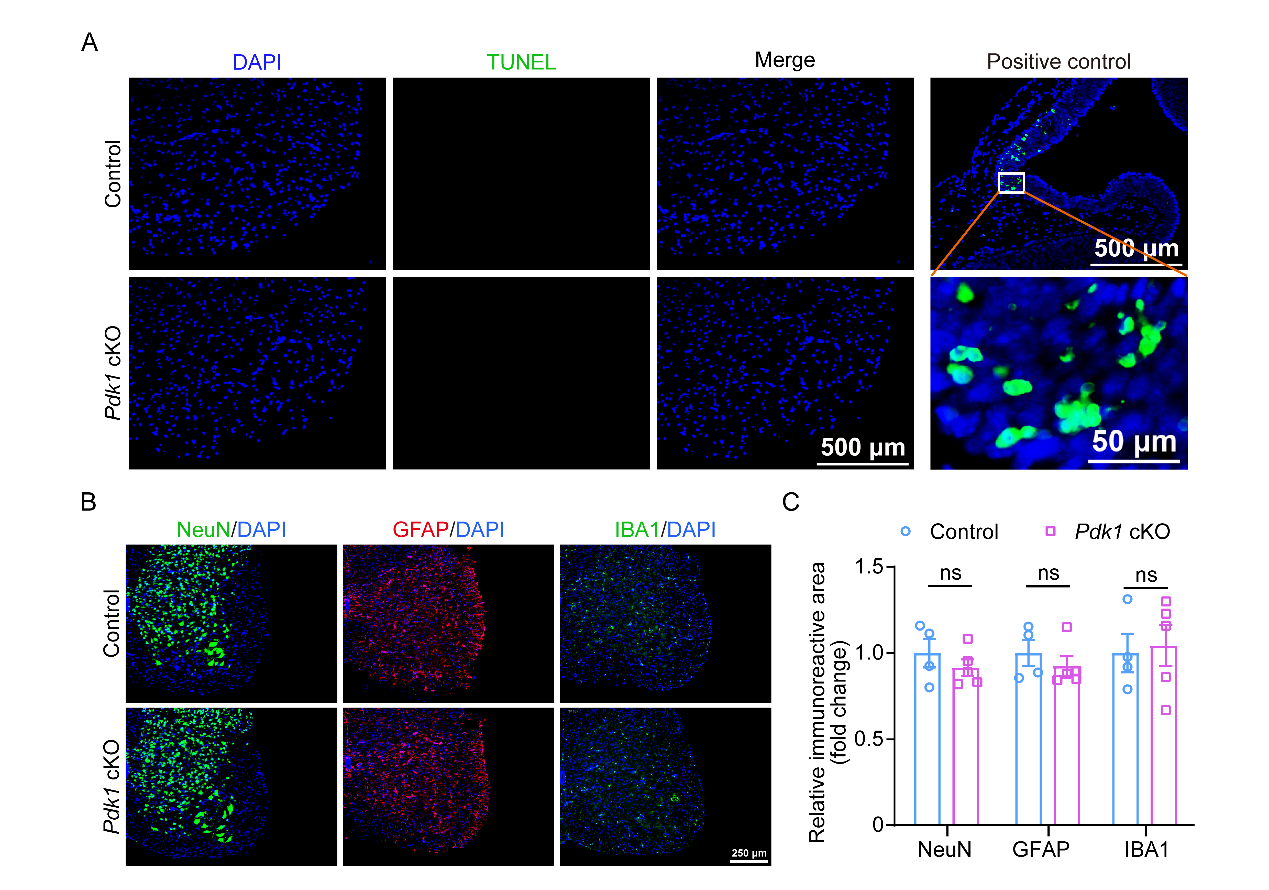


**Figure S6. Deletion of PDK1 did not affect cell survival or alter neuronal, astrocytic, or microglial populations in the spinal cords. A**. Representative images of the TUNEL (green) in the spinal cords. TUNEL staining was performed to assess apoptotic cell death in the spinal cords of control and *Pdk1* cKO mice. No significant differences in the number of TUNEL^+^ cells were observed between the two groups (n ≥ 4 mice per group). Scale bar = 500 μm and 50 μm. **B**. IHC staining of NeuN (left, green), GFAP (center, red), and IBA1 (right, green). Nuclei were counterstained with DAPI (blue). Scale bar = 250 μm. **C**. Quantification of the relative area occupied by NeuN^+^, GFAP^+^, and IBA1^+^ signals. IHC staining for NeuN/GFAP/IBA1 revealed no significant differences in the expression of those cells between control and *Pdk1* cKO mouse spinal cords (unpaired t test; mean ± SEM; n ≥ 4 mice per group). **p*< 0.05; ***p*< 0.01; ****p*< 0.001; *****p*< 0.0001; ns, no significant difference.4


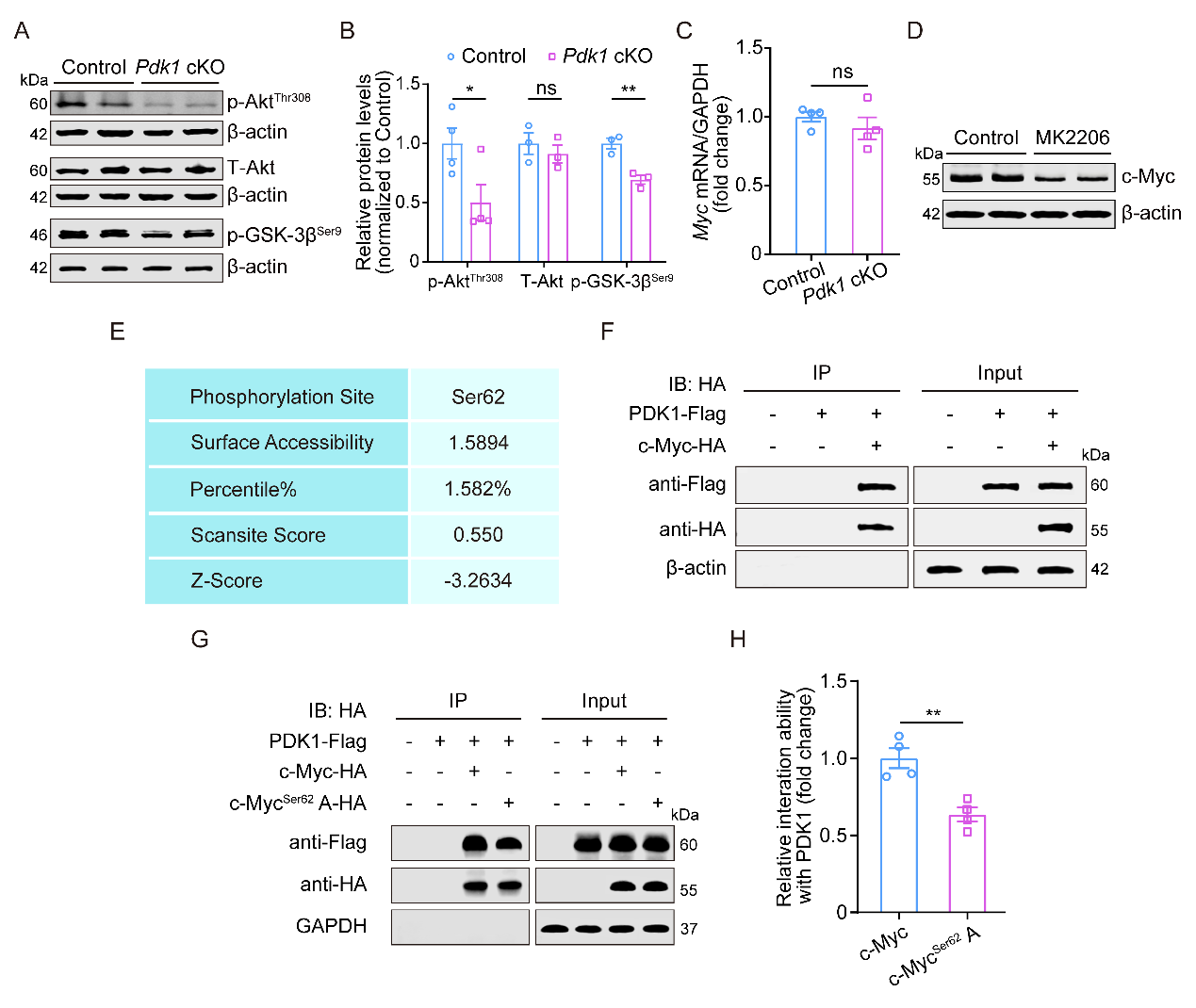


**Figure S7. Identification and validation of the PDK1-c-Myc interaction. A, B**. WB validation (**A**) and quantification (**B**) of p-Akt^Thr308^, T-Akt, and p-GSK-3β^Ser9^ in control and *Pdk1* cKO mouse spinal cords. The ratio of p-Akt^Thr308^/ T-Akt and p-GSK-3β^Ser9^ levels was significantly decreased in *Pdk1* cKO mice (unpaired t test; mean ± SEM; n ≥ 3 mice per group). **C**. qRT-PCR analysis of *Myc* showed an unchanged expression between the Control and *Pdk1* cKO spinal cords (unpaired t test; mean ± SEM; n = 4 mice per group). **D**. Representative WB images of c-Myc protein in primary OPCs treated with the Akt inhibitor MK2206. MK2206 treatment did not lead to an increase in c-Myc protein levels compared with control conditions (unpaired t test; mean ± SEM; n = 6 independent cultures). **E**. Table indicates Scansite 4.0 prediction of PDK1 recognition motifs within the c-Myc protein sequence. **F**. CO-IP assay showed a direct interaction between PDK1 and c-Myc. Lysates from Oli-neu cells co-transfected with PDK1-Flag and c-Myc-HA plasmids were immunoprecipitated using anti-HA beads, followed by WB with β-actin, anti-HA, and anti-Flag antibodies. A specific band corresponding to c-Myc was detected in the precipitated samples, indicating a direct interaction between PDK1 and c-Myc (n = 3 biological replicates for each experiment). **G**. Representative CO-IP images of PDK1, c-Myc, and c-Myc^Ser62^A. **H**. Quantification of the interaction of PDK1 with the Ser62 site of c-Myc. Lysates from Oli-neu cells co-transfected with PDK1-Flag and c-Myc-HA or c-Myc^Ser62^A-HA plasmids were immunoprecipitated using anti-HA beads, followed by WB with GAPDH, anti-HA, and anti-Flag antibodies. These results revealed that PDK1 recognized c-Myc at Ser62 (unpaired t test; mean ± SEM; n = 4 biological replicates for each experiment). **p*< 0.05; ***p*< 0.01; ****p*< 0.001; *****p*< 0.0001; ns, no significant difference.


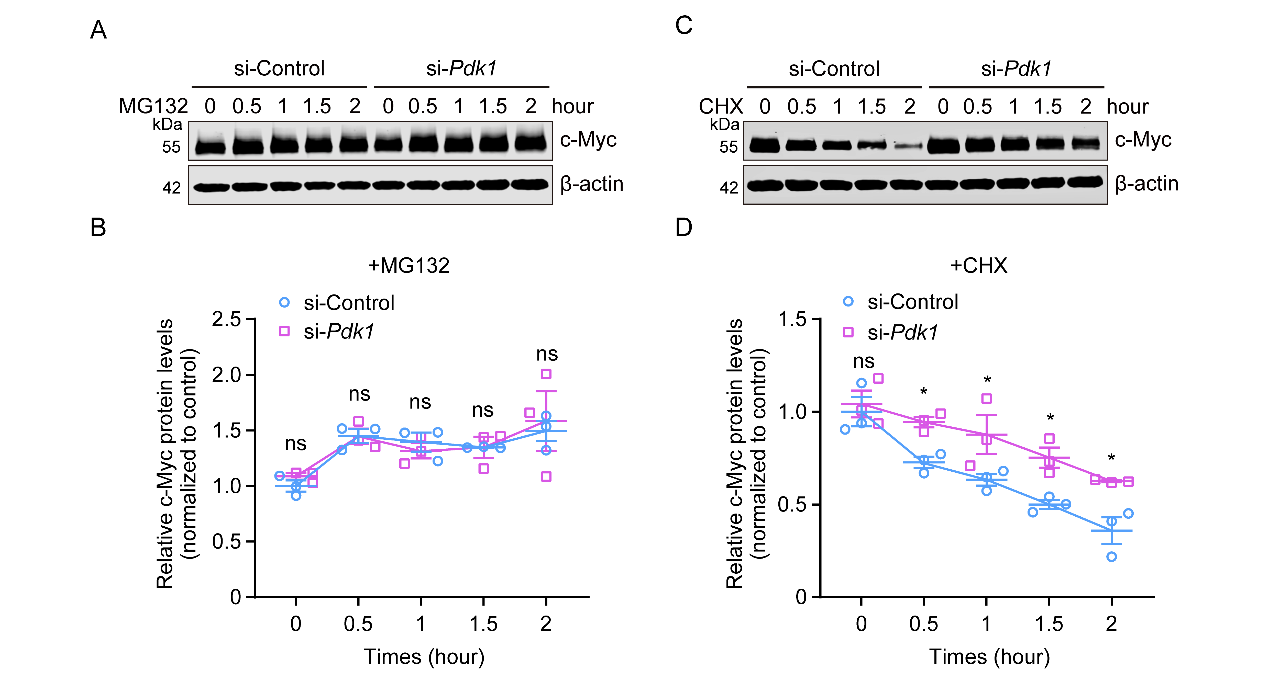


**Figure S8. PDK1 deletion inhibited c-Myc protein degradation without affecting its synthesis. A, C**. Representative WB images of c-Myc and β-actin. **B, D**. Quantification of the c-Myc protein levels in Oli-neu cells under the treatment with MG132 (**B**) and CHX (**D**) in the presence or absence of PDK1. PDK1 depletion significantly blocked the degradation of c-Myc (CHX-treated groups) but did not affect the synthesis of c-Myc (MG132-treated groups) (two-way ANOVA; mean ± SEM; n = 3 biological replicates for each experiment). **p*< 0.05; ***p*< 0.01; ****p*< 0.001; *****p*< 0.0001; ns, no significant difference.


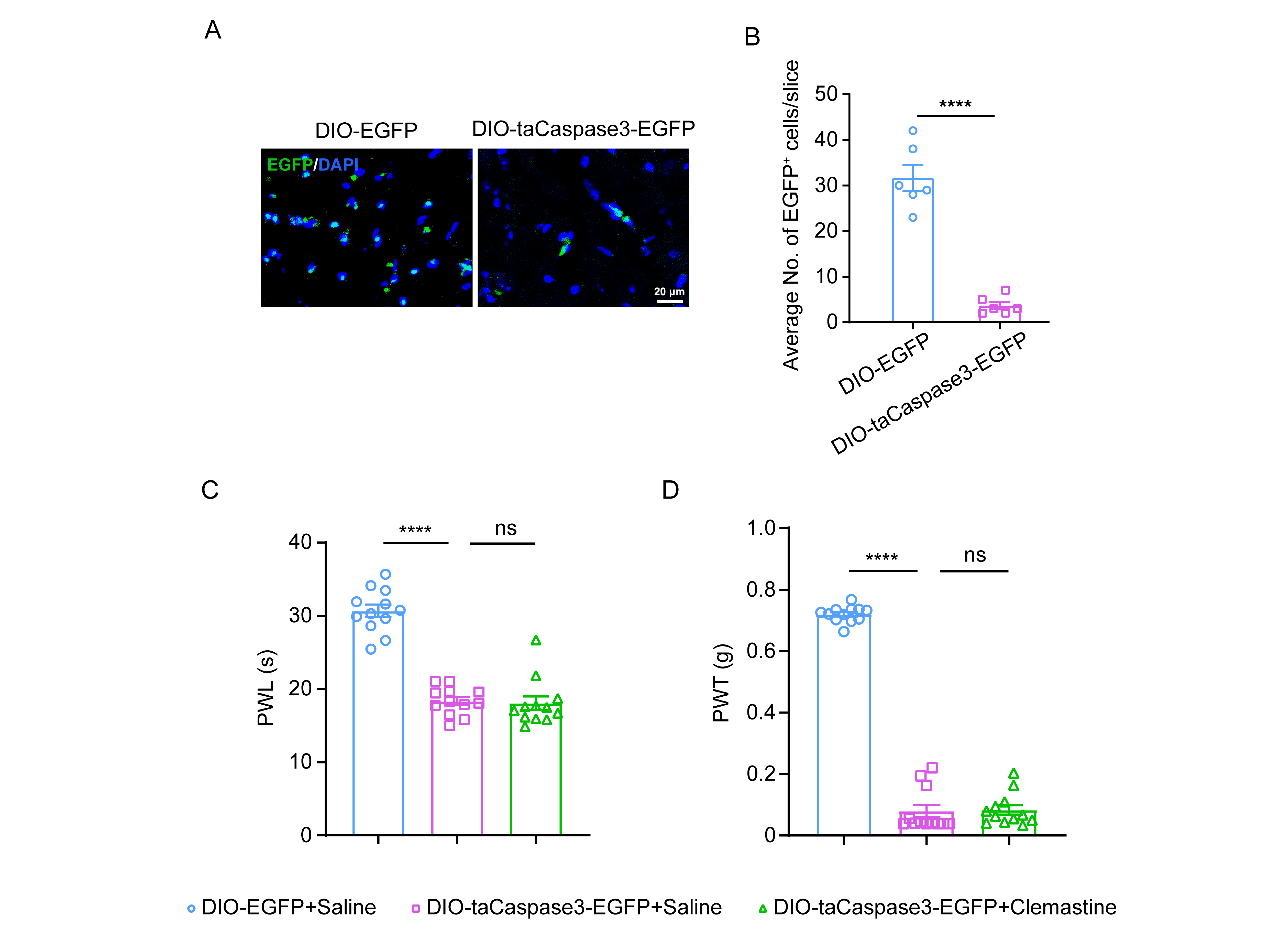


**Figure S9. The analgesic efficacy of clemastine was specifically dependent on the OL lineage cells.** **A**. Representative immunofluorescence images showing EGFP-labeled OLs (green) and DAPI-stained nuclei (blue) in the spinal cord white matter (STT region) of *NG2-CreERT2* mice obtained 21 days after injection with either rAAV-DIO-EGFP or rAAV-DIO-taCaspase3-EGFP. Scale bar = 20 μm. **B**. Quantitative analysis of the average number of EGFP^+^ OLs in STT at 21 days post-injection. The significant reduction in the DIO-taCaspase3-EGFP group confirms the successful ablation of the OLs in the targeted region (unpaired t test; mean ± SEM; n = 6 mice per group). **C, D**. Behavioral assessments of thermal and mechanical hypersensitivity using the PWL test (**C**) and PWT test (**D**) were conducted 21 days after viral injection. Ablation of OLs (DIO-taCaspase3-EGFP+Saline) significantly reduced the threshold for both PWL and PWT test compared to control mice (DIO-EGFP + Saline). Crucially, the daily administration of clemastine for 21 consecutive days in OL-ablated mice (DIO-taCaspase3-EGFP+Clemastine) failed to alleviate pain hypersensitivity, showing no significant difference compared to the saline-treated ablated group (One-way ANOVA; mean ± SEM; n = 12 mice per group). **p*< 0.05; ***p*< 0.01; ****p*< 0.001; *****p*< 0.0001; ns, no significant difference.

**Table S1.**

List of antibodies.

| Antibodies | Source | Identifier |
| --- | --- | --- |
| Rabbit monoclonal anti-PDK1 | Abcam | Cat# ab52893, RRID: AB_881962 |
| Mouse monoclonal anti-CC1 | Calbiochem | Cat# op-80, RRID: AB_2057371 |
| Mouse monoclonal anti-Olig2 | Millipore | Cat# MABN50, RRID: AB_10807410 |
| Rabbit monoclonal anti-SOX10 | Abcam | Cat# ab155279, RRID: AB_2650603 |
| Mouse monoclonal anti-SOX10 | Santa Cruz | Cat# A-2, RRID: sc36569-c |
| Rat monoclonal anti-BrdU | Abcam | Cat# ab6326, RRID: AB_305426 |
| Rabbit monoclonal anti-PDGFRα | Cell Signaling Technology | Cat# 3174, RRID: AB_2162345 |
| Rat polyclonal anti-PLP1 | Oasis | Cat# OB-PRT040, RRID: AB_2938822 |
| Rabbit polyclonal anti-PLP1 | Abclonal | Cat# A20009, RRID: [AB_2862916](http://antibodyregistry.org/AB_2862916) |
| Rat monoclonal anti-MBP | Millipore | Cat# MAB386, RRID: AB_94975 |
| Mouse monoclonal anti-CASPR/Neurexin IV | NeuroMab | Cat# 75-001-FL594, RRID: AB_2939066 |
| Rabbit polyclonal anti-Na_v_1.6 | Alomone labs | Cat# ASC-009, RRID: AB_2040202 |
| Rabbit monoclonal anti- phospho-Akt (Thr308) | Cell Signaling Technology | Cat# 13038, RRID: AB_2629447 |
| Rabbit polyclonal anti-GSK-3β (Ser9) | Cell Signaling Technology | Cat# 5558, RRID: D85E12 |
| Rabbit monoclonal anti-phospho-c-Myc (Ser62) | Abclonal | Cat# AP0989, RRID: AB_2863883 |
| Rabbit monoclonal anti-phospho-c-Myc (Thr58) | Abclonal | Cat# AP0990, RRID: AB_2863884 |
| Mouse monoclonal anti-c-Myc | Proteintech | Cat# 67447-1-Ig, RRID:  AB_2882681 |
| Rabbit polyclonal anti-NeuN | Millipore | Cat# ABN78, RRID: AB _10807945 |
| Mouse monoclonal anti-GFAP | Santa Cruz | Cat# sc-65,343, RRID: AB_783553 |
| Rabbit polyclonal anti-IBA1 | WAKO | Cat# 019-19741, RRID: AB_839504 |
| Rabbit polyclonal anti-β-actin | GenTex | Cat# CTX124212, RRID: N/A |
| Mouse monoclonal anti-GAPDH | CW Biotech | Cat# cw0100, RRID: N/A |
| Mouse polyclonal anti-HA | Cell Signaling Technology | Cat# 2367, RRID: AB_10691311 |
| Rabbit monoclonal anti-FLAG | Cell Signaling Technology | Cat# 2368, RRID: AB_2217020 |
| Rabbit polyclonal anti-EGFP | Proteintech | Cat# 50430-2-AP, RRID: AB_11042881 |
| Rabbit polyclonal anti-PDGFRβ | Abclonal | Cat# A1195, RRID: AB_2758888 |
| Mouse monoclonal anti-MAP2 | Millipore | Cat# MAB3418, RRID: AB_94856 |
| Mouse monoclonal anti-MAP2 | Millipore | Cat# MAB3418, RRID: AB_94856 |
| Rabbit polyclonal anti-Synaptophysin | Abcam | Cat# ab14692, RRID: AB_301417 |
| Rabbit polyclonal anti-Homer1 | Synaptic Systems | Cat# 160003, RRID: AB_887730 |
| Rabbit polyclonal anti-VGAT | Synaptic Systems | Cat# 131 003, RRID: AB_887869 |
| Alexa Fluor® 488 AffiniPure® Goat Anti-Rabbit IgG (H+L) | Jackson ImmunoResearch Labs | Cat# 111–545-003, RRID: AB_2338046 |
| Alexa Fluor® 594 AffiniPure® Goat Anti-Mouse IgG (H+L) | Jackson ImmunoResearch Labs | Cat# 115-585-003, RRID: AB_2338871 |
| Alexa Fluor® 488 AffiniPure® Donkey Anti-Rat IgG (H+L) | Jackson ImmunoResearch Labs | Cat# 712-545-150, RRID: AB_2340683 |
| Biotin-AffiniPure Goat Anti-Mouse IgG (H+L) | Jackson ImmunoResearch Labs | Cat# 111–545-003, RRID: AB_2338046 |
| Biotin-AffiniPure Goat Anti-Rabbit IgG (H+L) | Jackson ImmunoResearch Labs | Cat# 111–065-003, RRID: AB_2337959 |
| Goat Anti-Rabbit IgG, IRDye® 800CW Conjugated antibody | LI-COR Biosciences | Cat# 926–32211, RRID: AB_621843 |
| Goat Anti-Mouse IgG, IRDye® 800CW Conjugated antibody | LI-COR Biosciences | Cat# 926–32210, RRID: AB_621842 |
| Goat Anti-Rat IgG, IRDye® 800CW Conjugated antibody | LI-COR Biosciences | Cat# 926-32219, RRID: AB_621851 |

**Table S2.**

List of primers for qRT-PCR.

| Name | Primer Sequence 5’-3’ | Identifier |
| --- | --- | --- |
| *Pdk1*-F | CCTTCAGGAGTTGCTTGATTTT | N/A |
| *Pdk1-R* | ACATTTTGGCTGGTGACAGG | N/A |
| *Olig2*-F | ACCACGTGTCGGCTATGG | N/A |
| *Olig2*-R | CCATAATCCCCTAGGCCCAG | N/A |
| *Sox10*-F | CAGTACCCTCACCTCCACAA | N/A |
| *Sox10*-R | CGCCGAGGTTGGTACTTGTA | N/A |
| *Myrf*-F | TCTAACCCCAAGCACTCAGG | N/A |
| *Myrf*-R | GTTCTTGGTCTTGCTCTGCC | N/A |
| *Mbp*-F | CACACACGAGAACTACCCA | N/A |
| *Mbp*-R | GGTGTTCGAGGTGTCACAA | N/A |
| *Plp1*-F | TCCCTAGCAAGACCTCTGC | N/A |
| *Plp1*-R | CATGAGTTTAAGGACGGCG | N/A |
| *Mag*-F | CAGATCCTAGCCACGGTCAT | N/A |
| *Mag*-R | CACACATAGACACTGCACGG | N/A |
| *Mog*-F | AAGTGCGATGAGAGTCAGC | N/A |
| *Mog*-R | AAGTGCGATGAGAGTCAGC | N/A |
| *Hes1*-F | GCACAGAAAGTCATCAAAGCC | N/A |
| *Hes1*-R | TTCCAGAATGTCTGCCTTCTC | N/A |
| *Hes5*-F | AACACAGCAAAGCCTTCGCC | N/A |
| *Hes5*-R | AAGCAGCTTCATCTGCGTGTC | N/A |
| *Id2*-F | GCATCCCACTATCGTCAG | N/A |
| *Id2*-R | TTCAGATGCCTGCAAGGAC | N/A |
| *Id4*-F | AGACTCACCCTGCTTTGCTGAGAC | N/A |
| *Id4*-R | ATGCTGTCACCCTGCTTGTTCAC | N/A |
| *Lef1*-F | CCGACATCAAGTCATCTTTGG | N/A |
| *Lef1*-R | CTGGATGCTTTCCTTCATCAG | N/A |
| *Notum*-F | GGACAGCTTTATGGCGCAAG | N/A |
| *Notum*-R | TCACCGACGTGTTCAGCAG | N/A |
| *Tcf4*-F | GACCACACGAACAACAGCTT | N/A |
| *Tcf4*-R | TCTTCGATTCGGCTTTGCAG | N/A |
| *Axin2*-F | AACCTATGCCCGTTTCCTCTA | N/A |
| *Axin2*-R | GAGTGTAAAGACTTGGTCCACC | N/A |
| *Myc*-F | CGCGCCCAGTGAGGATATC | N/A |
| *Myc*-R | CCACATACAGTCCTGGATGAT | N/A |
| *Tnf-F* | CTGAACTTCGGGGTGATCGG | N/A |
| *Tnf-R* | GGCTTGTCACTCGAATTTTGAGA | N/A |
| *Il1b-F* | CAACCAACAAGTGATATTCTCCATG | N/A |
| *Il1b-R* | GATCCACACTCTCCAGCTGCA | N/A |
| *Il13-F* | ATGAGTCTGCAGTATCCCG | N/A |
| *Il13-R* | CCGTGGCAGACAGGAGTGTT | N/A |
| *Il6-F* | AAGTCGGAGGCTTAATTACACATGT | N/A |
| *Il6-R* | CCATTGCACAACTCTTTTCTCATTC | N/A |
| *Ifng-F* | AGCCCTATTACAGCACAG | N/A |
| *Ifng-R* | TTCTAACAACAAGTATCCC | N/A |
| *Gapdh*-F | CCTTCATTGACCTCAACTACATG | N/A |
| *Gapdh*-R | CTTCTCCATGGTGGTGAAGAC | N/A |

**Table S3.**

Chemicals and Recombinant Proteins

| Name | Source | Identifier |  |
| --- | --- | --- | --- |
| TrueGold myelin kit | Oasis | Cat# BK-AC001 | |
| RNAiso Plus | Takara | Cat# 9109 | |
| HiScript II Q RT SuperMix for qPCR (+gDNA wiper) | Vazyme | Cat# R223-01 | |
| ChamQ SYBR qPCR Master Mix | Vazyme | Cat# Q311-02 | |
| Tamoxifen | Sigma-Aldrich | Cat# H6278 | |
| Clemastine | Selleck | Cat# S1847 | |
| BrdU | Sigma-Aldrich | Cat# B5002 | |
| DAPI | Sigma-Aldrich | Cat# D9542 | |
| CHX | MedChemExpress | Cat# HY-12320 | |
| MG-132 | MedChemExpress | Cat# HY-13259 | |
| MK2206 | MedChemExpress | Cat# HY-108232 | |

**Table S4.**

Software and Algorithms

| Name | Source | Identifier | |
| --- | --- | --- | --- |
| Leica confocal software | http://softadvice.informer.com/Leica_Confocal_Software.html | N/A |  |
| Olympus camera software | https://www.olympuslifescience.com/en/support/downloads/ | N/A |  |
| SS-MCS Microscopic Confocal Scanning System | http://www.ss-raman.com/pd.jsp?id=50 | N/A |  |
| LI-COR Image Studio | http://www.dxy.cn/bbs/topic/34994594 | N/A |  |
| GraphPad Prism 8 | https://www.graphpad.com/scientificsoftware/prism/ | N/A |  |
| Adobe Photoshop CS6 | https://creative.adobe.com/products/dow | N/A |  |
| Adobe Illustrator | https://creative.adobe.com/products/dow | N/A |  |
| Image J | https://imagej.nih.gov/ij/download.html | N/A |  |
