# Supplementary material for: Targeting the PDK1/c‐Myc/SOX10 Signaling in Oligodendrocytes Alleviates Neuropathic Pain
Source: Adv Sci (Weinh). 2026 Apr 16;13(39):e16426. doi: 10.1002/advs.202516426 (PMC13334879; doi:10.1002/advs.202516426)

Supporting Information II

All Original Western Blot Images

Original Western Blot Images for Figure 1E

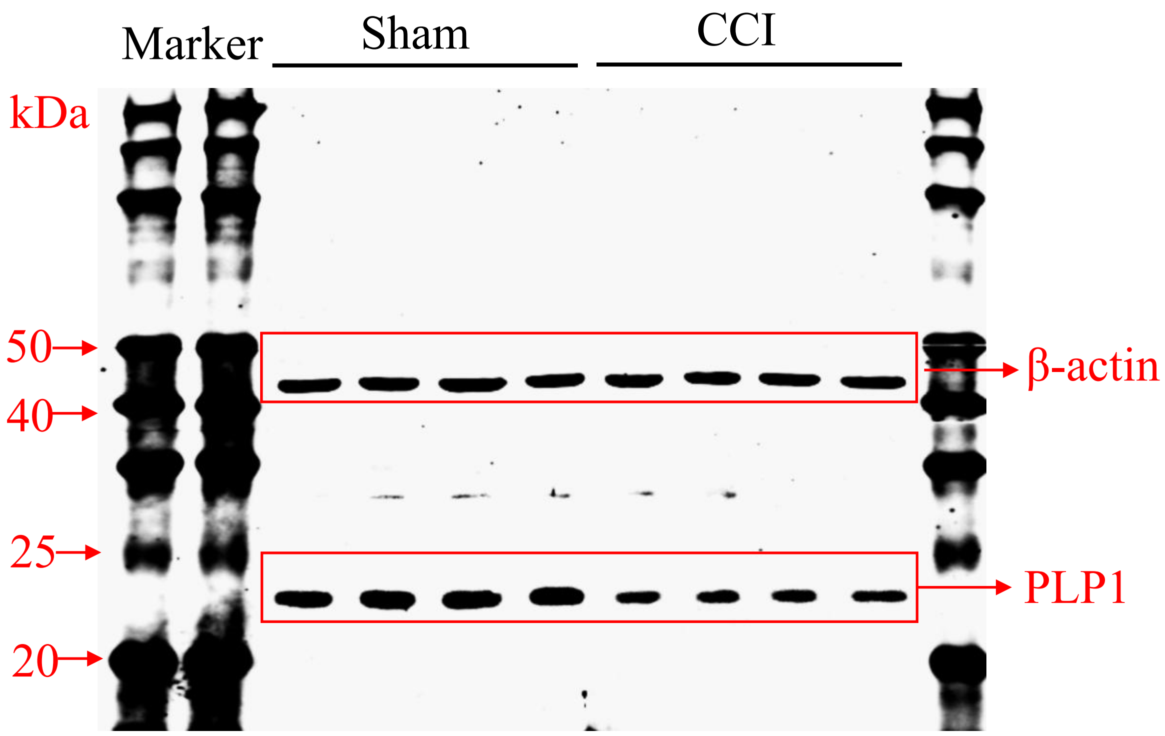

Original Western Blot Images for Figure 1Q

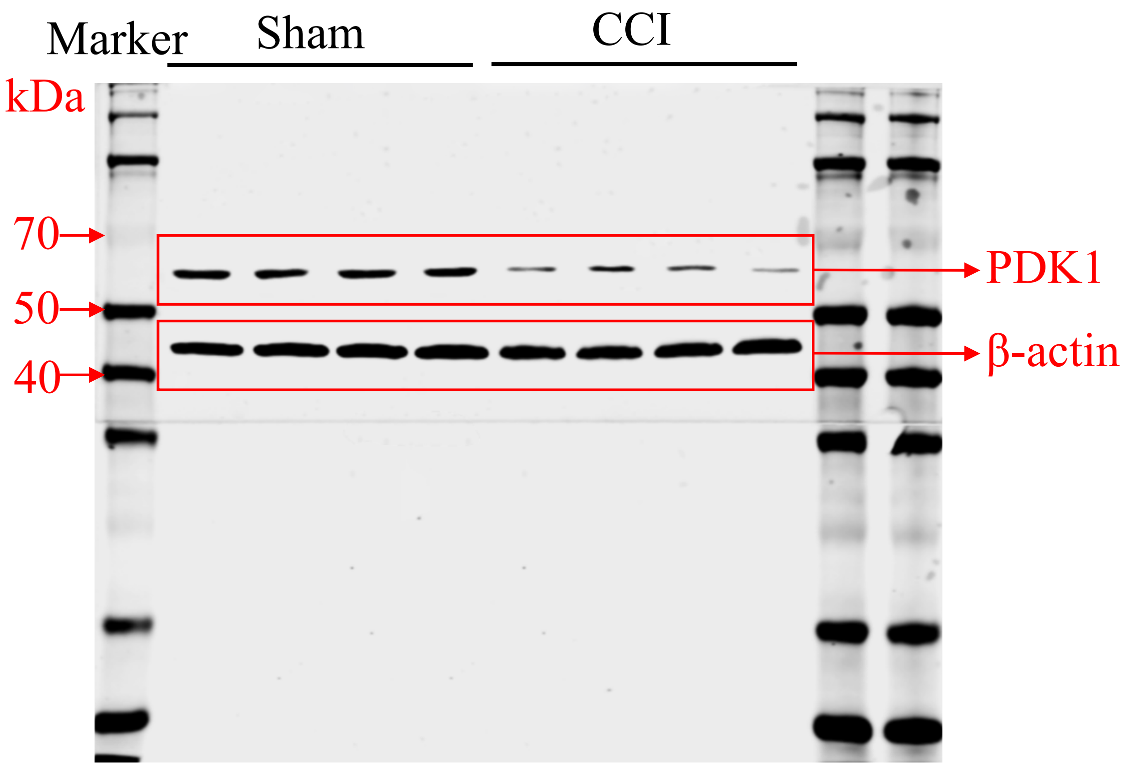

Original Western Blot Images for Figure 3F

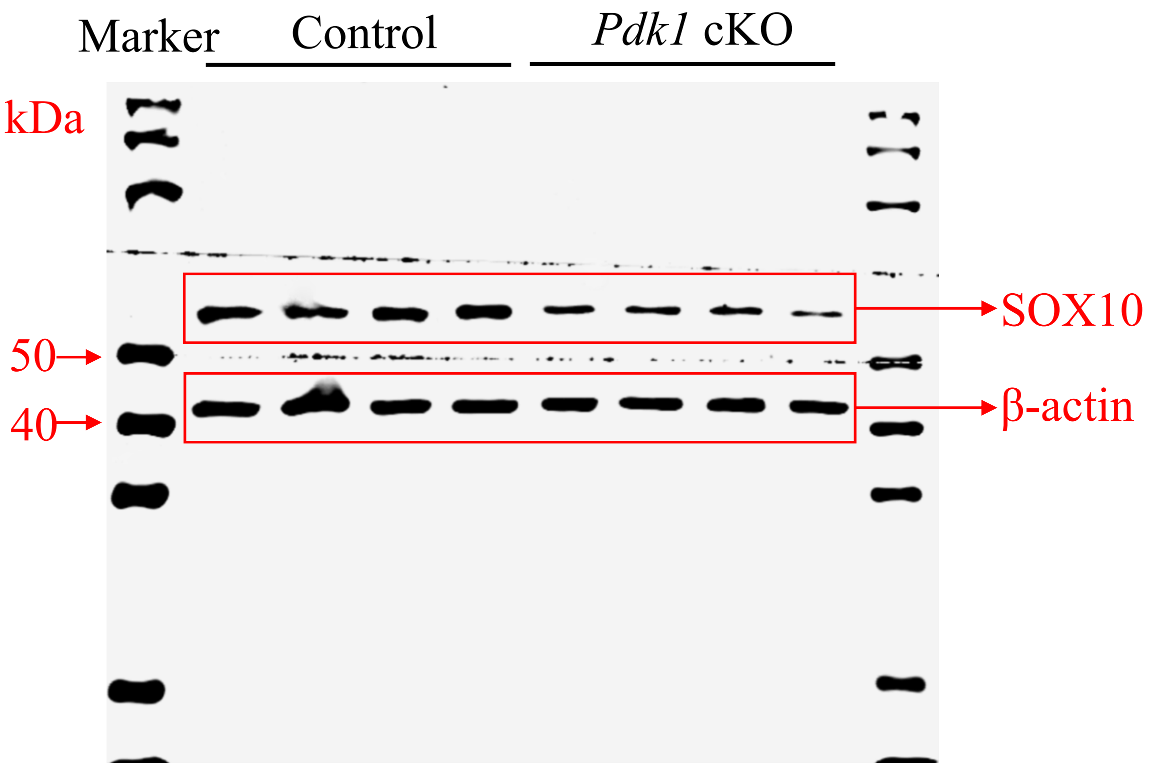

Original Western Blot Images for Figure 4A

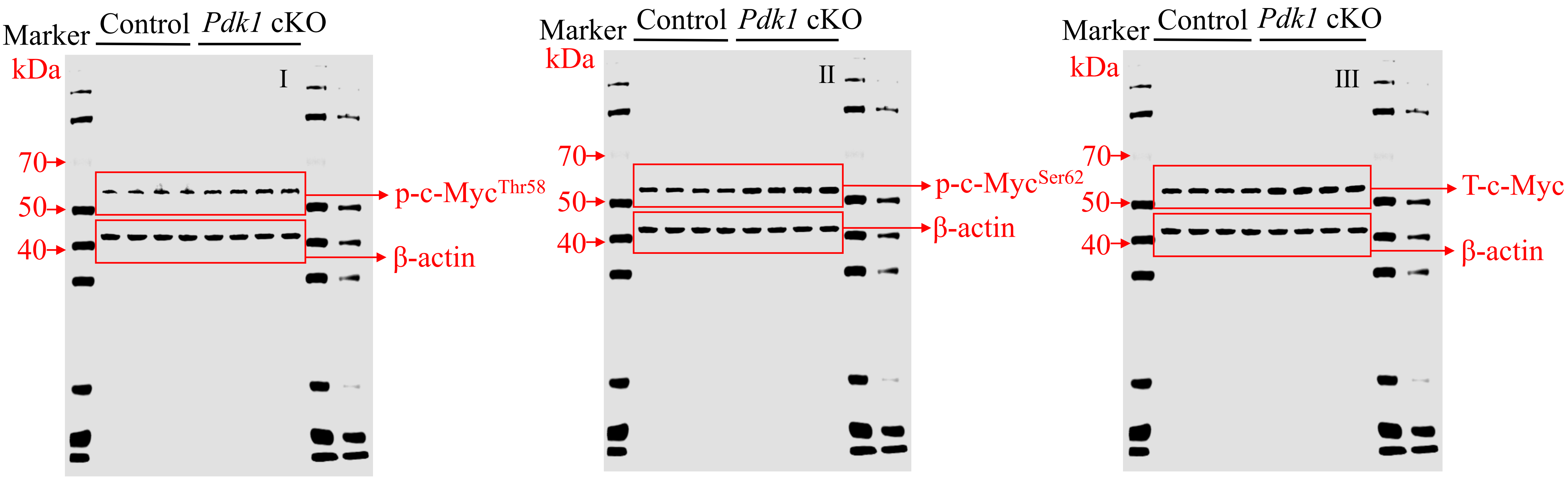

We would like to clarify that the p-c-Myc ^{Thr58} (image I), p-c-Myc ^{Ser62} (image II), and T-c-Myc (image III) signals were derived from the same membrane. The membrane was incubated with the corresponding primary antibodies and then detected using different secondary antibodies. Images were acquired separately in different channels using Image Studio.

Therefore, different target bands could be visualized on the same membrane through separate detection channels.

Original Western Blot Images for Figure 4C

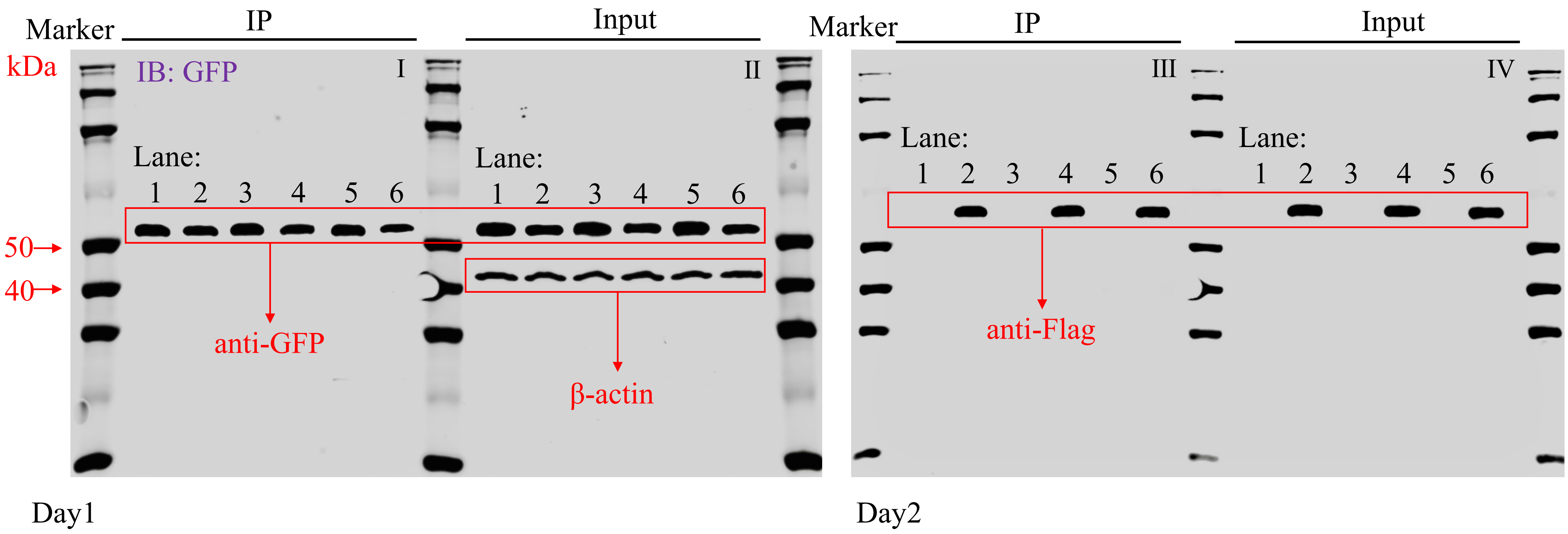

Original Western Blot Images for Figure 4C

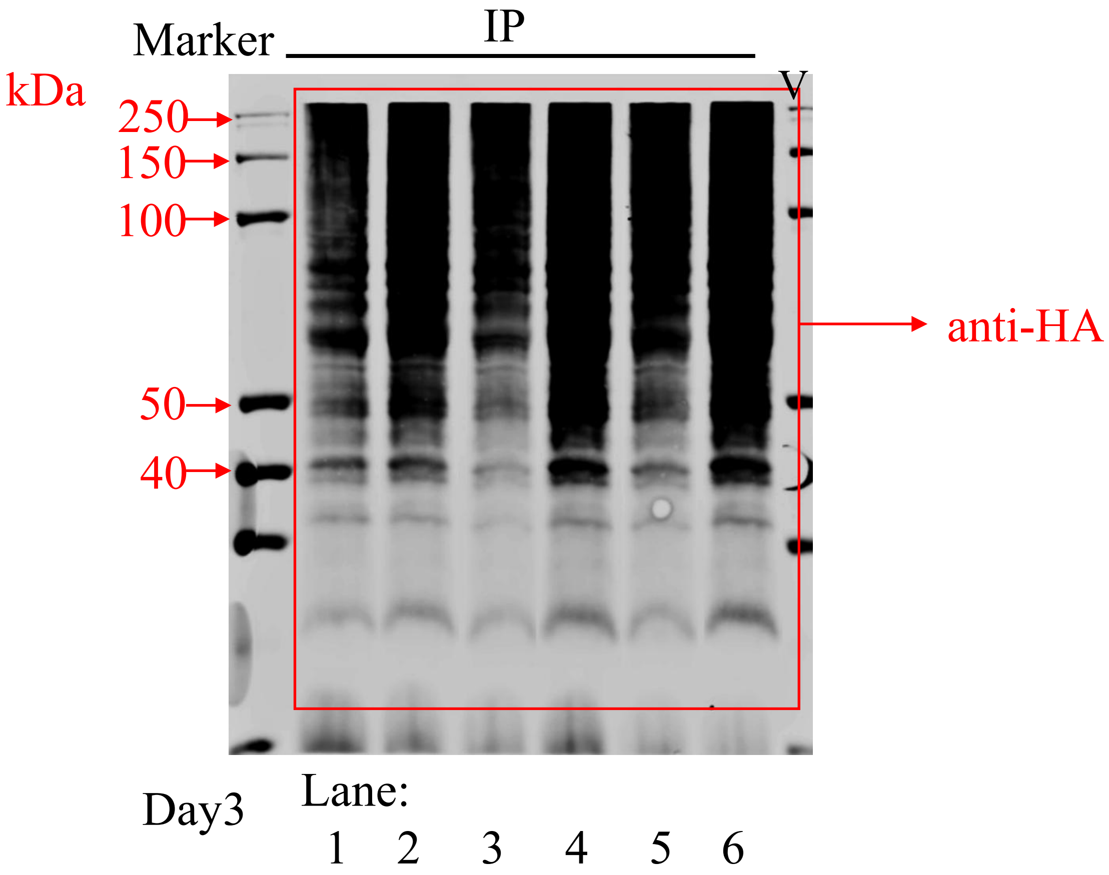

Lane:	1	2	3	4	5	6
PDK1-Flag:	-	+	-	+	-	+
c-Myc-GFP:	+	+	+	+	+	+
Ubiquitin-HA:	+	+	+	+	+	+

I, III, and V: derived from the same membrane as in Figure 4C.
II and IV: derived from the same membrane as in Figure 4C.

Original Western Blot Images for Figure 4E

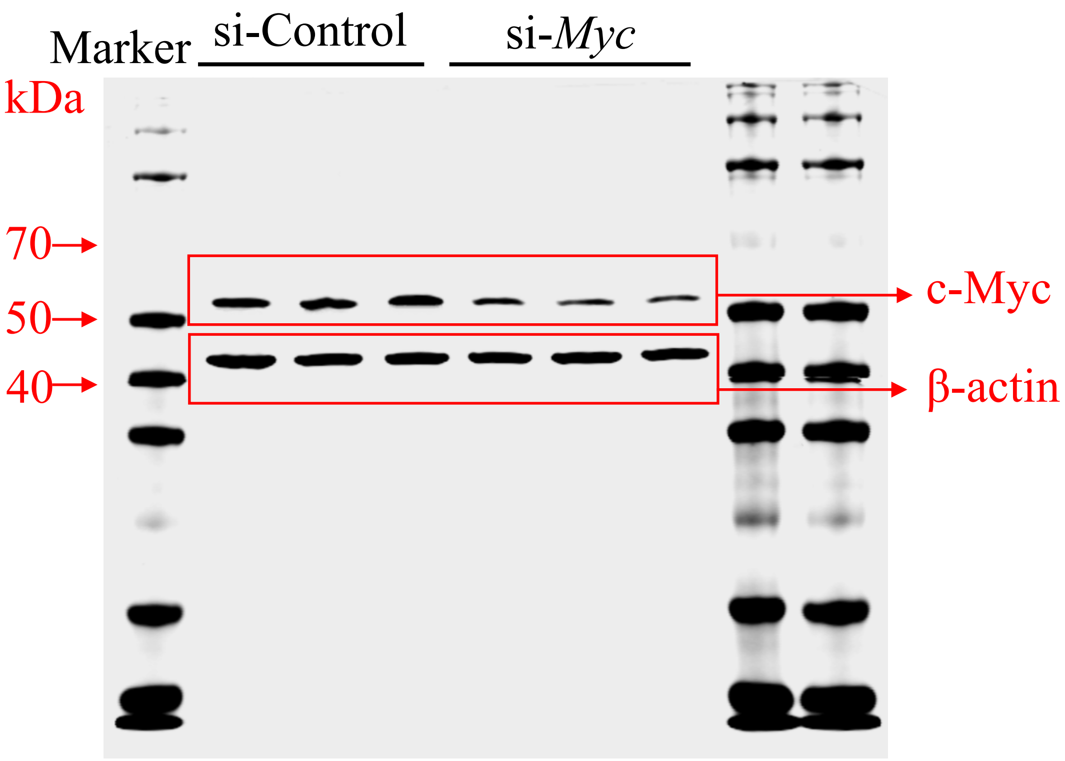

Original Western Blot Images for Figure 4H&L

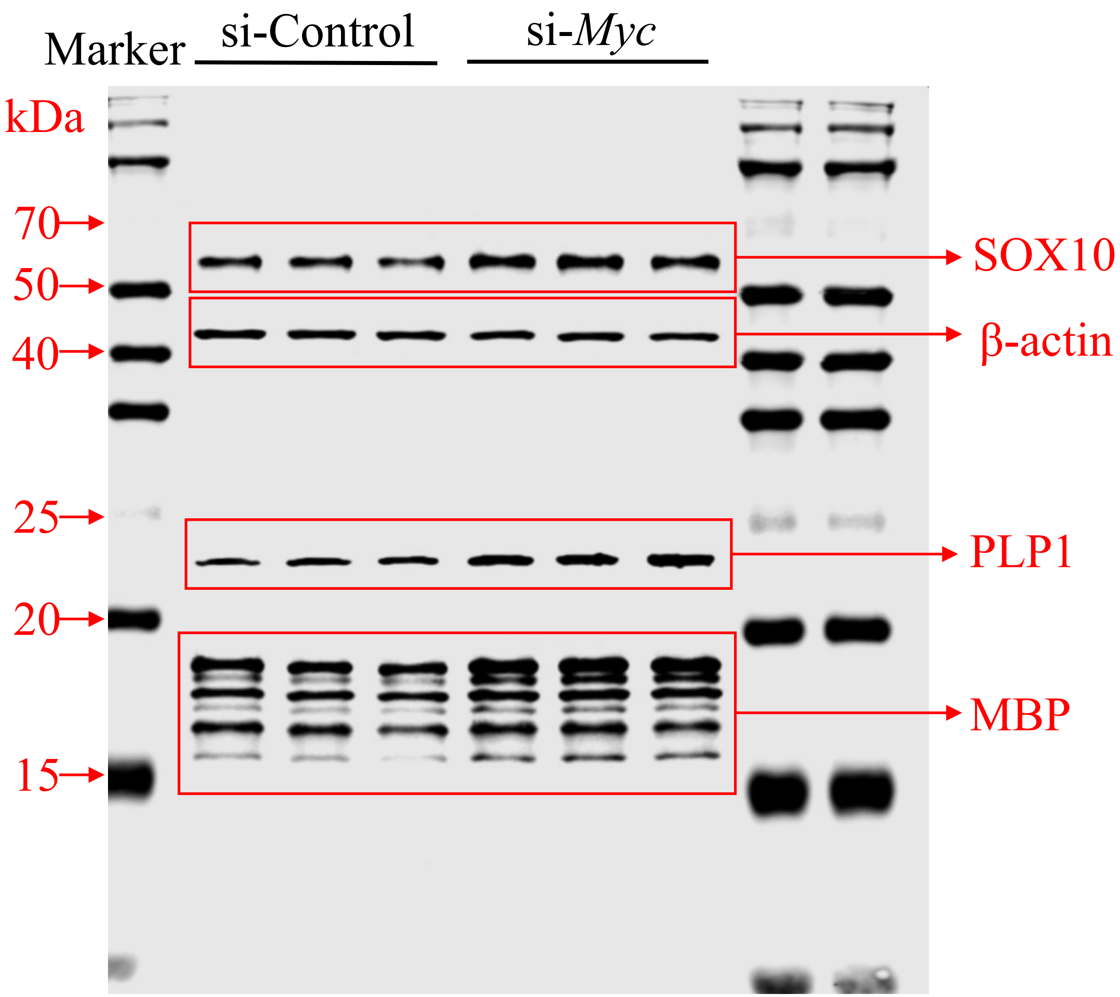

Original Western Blot Images for Figure 4O

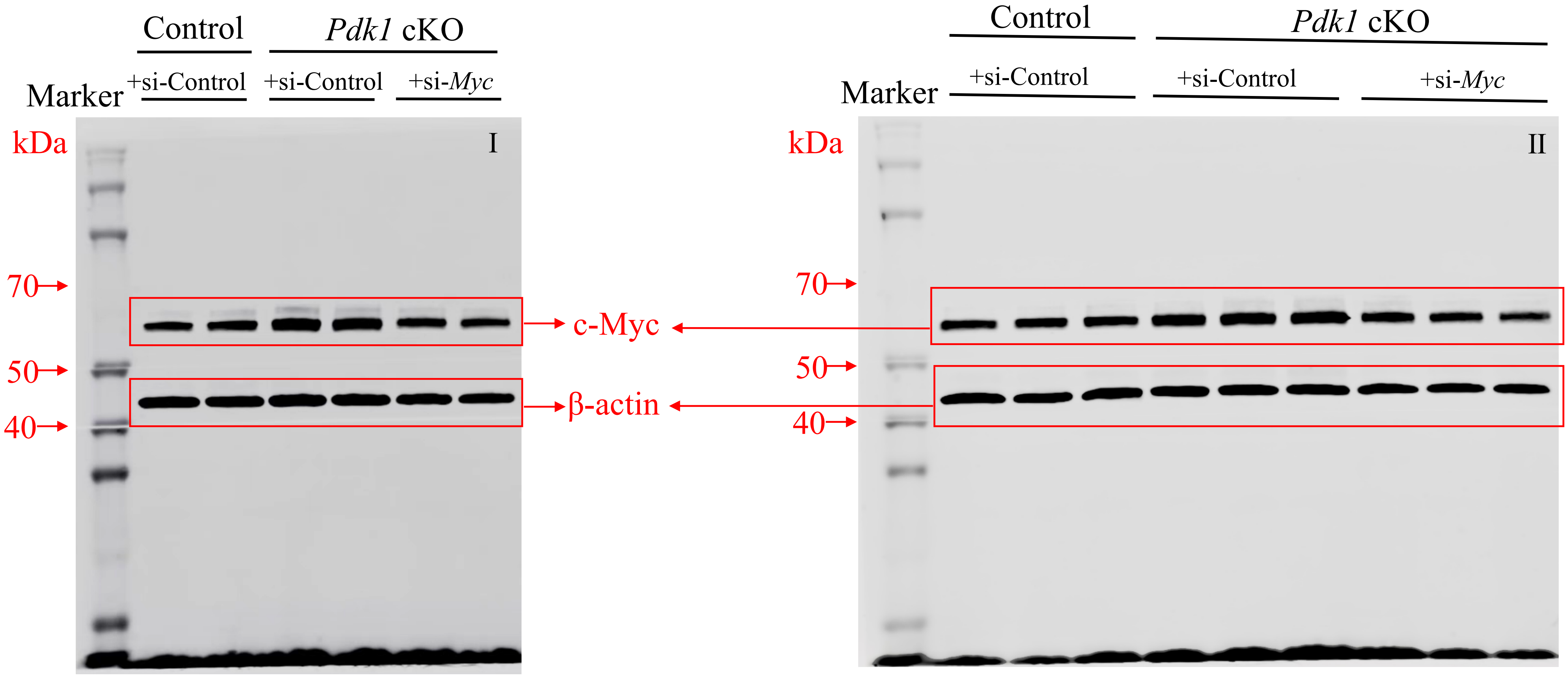

Original Western Blot Images for Figure 4O

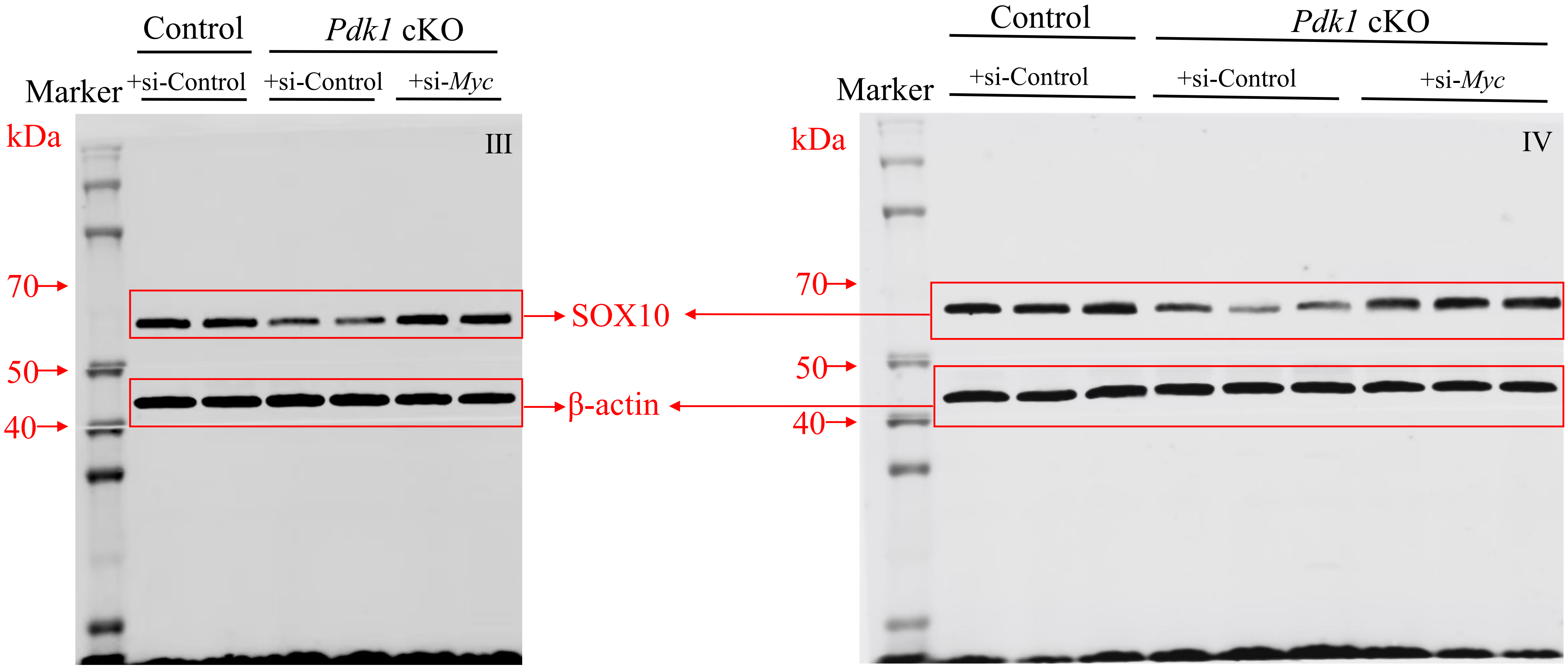

I and III: obtained from the same membrane as in Figure 4O.
II and IV: obtained from the same membrane as in Figure 4O.

Original Western Blot Images for Figure 5C

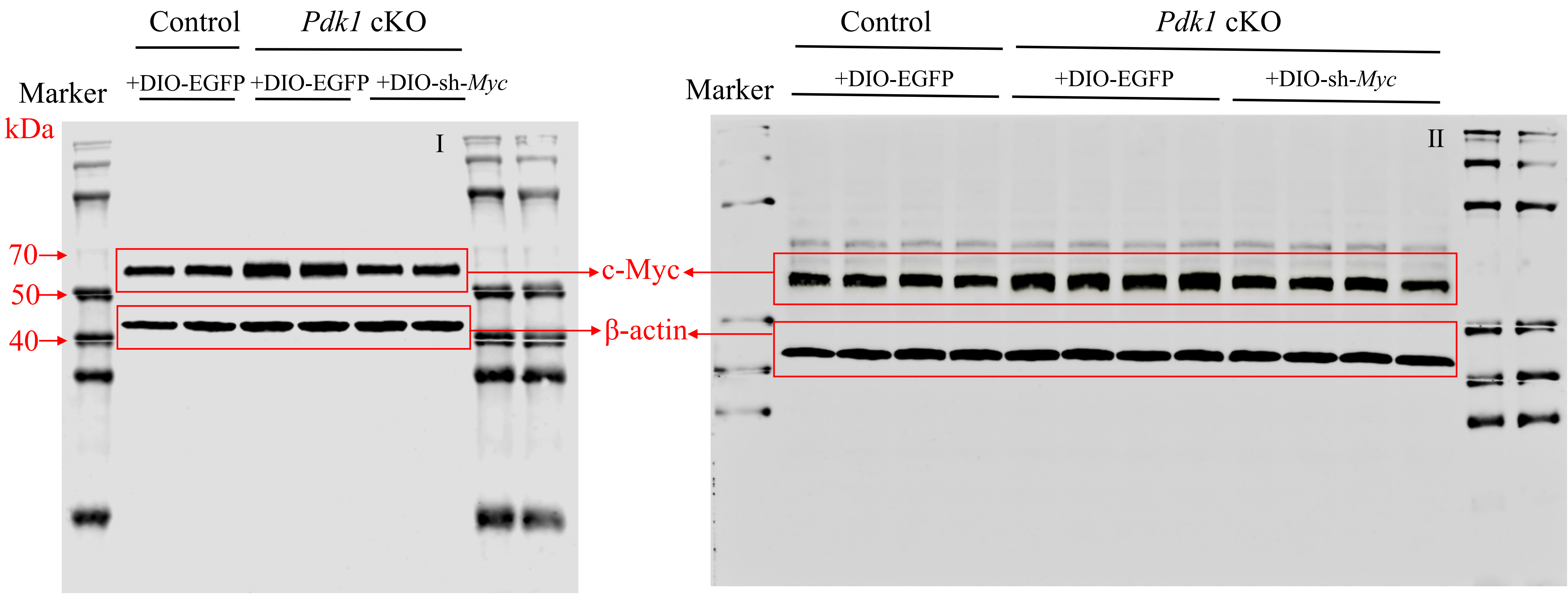

Original Western Blot Images for Figure 5C

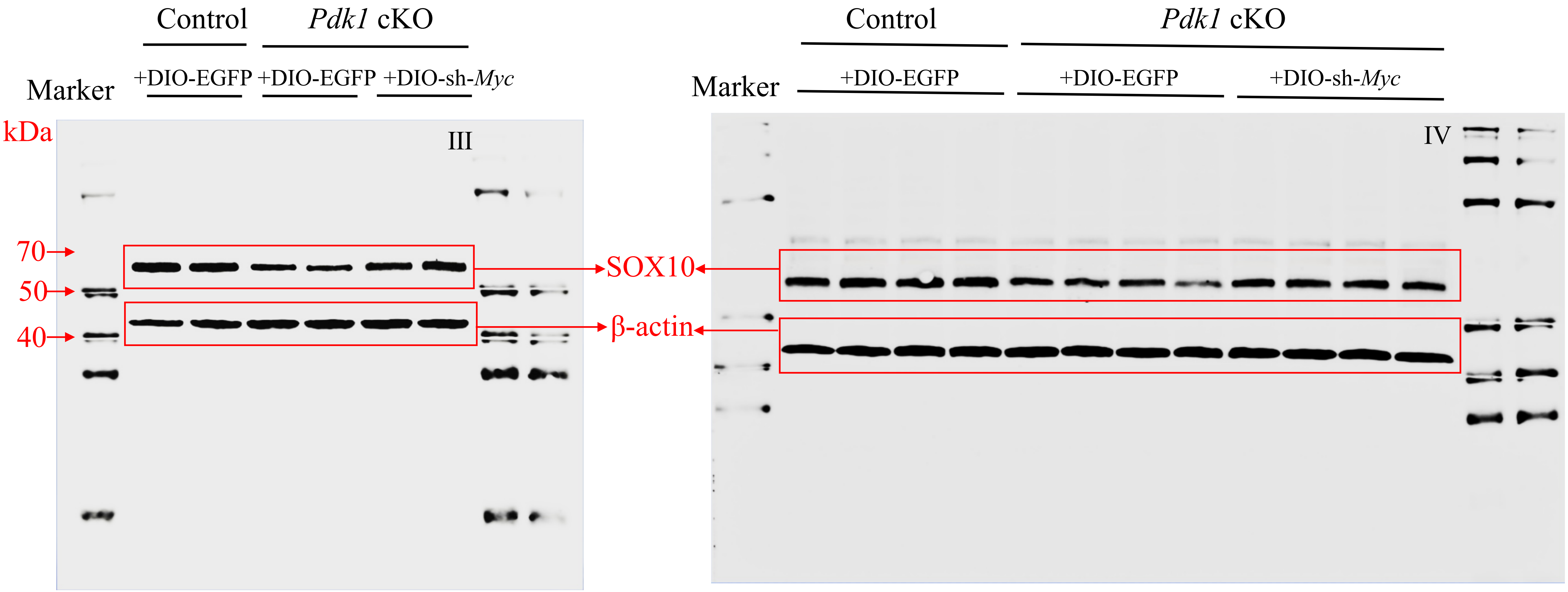

I and III: obtained from the same membrane as in Figure 5C.
II and IV: obtained from the same membrane as in Figure 5C.

Original Western Blot Images for Figure 6B

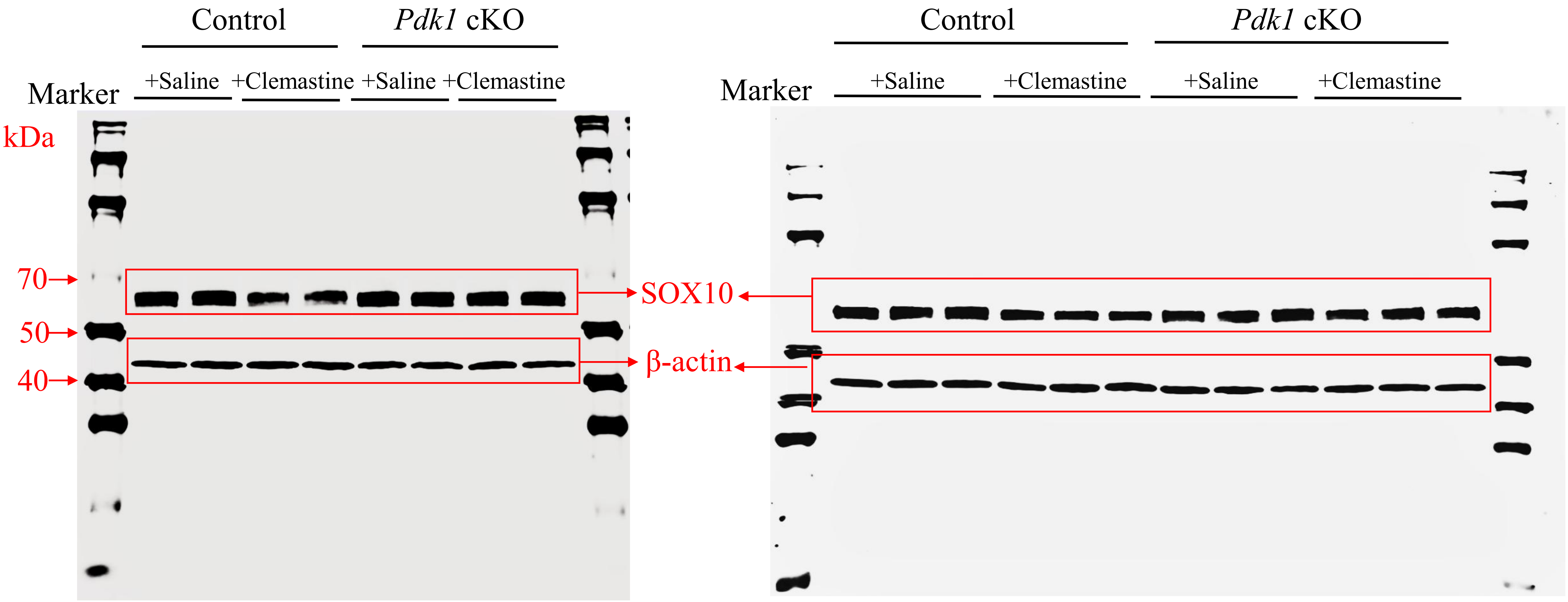

Original Western Blot Images for Figure 7C

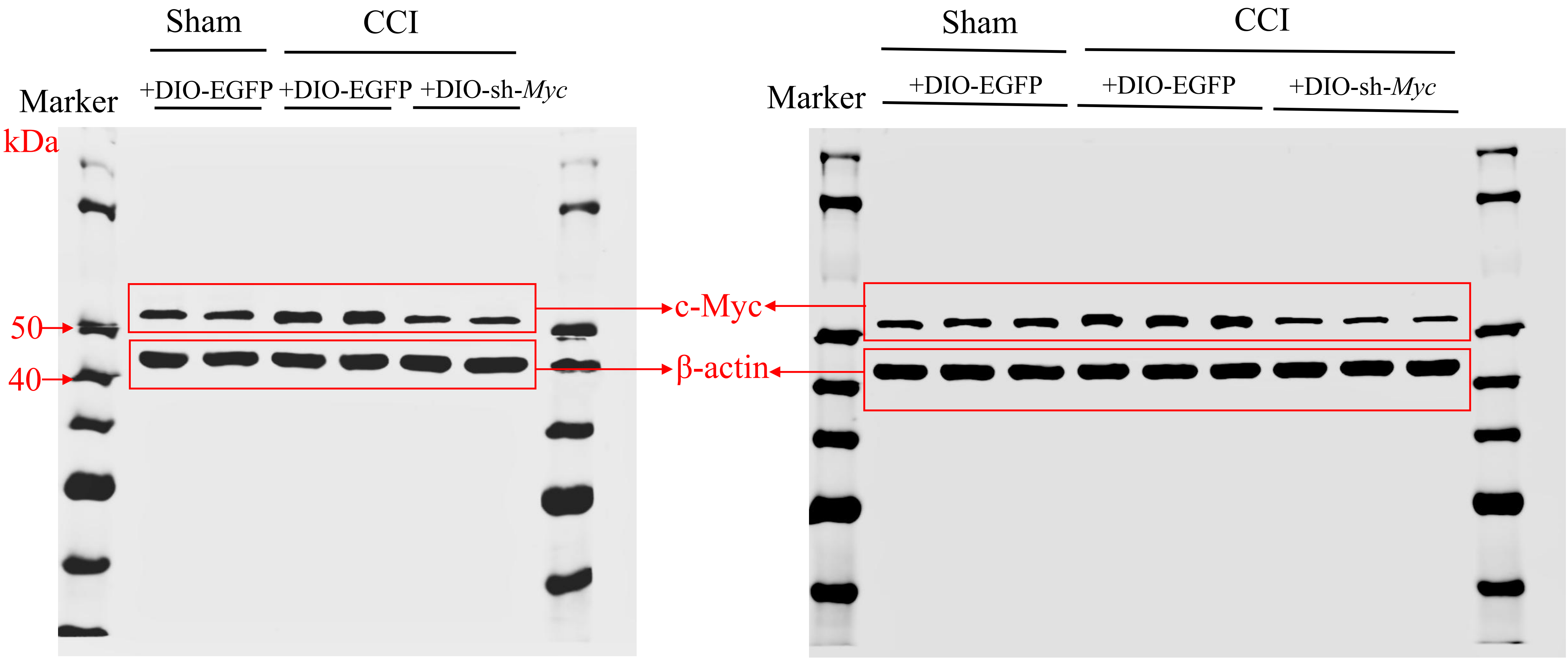

Original Western Blot Images for Figure 7G

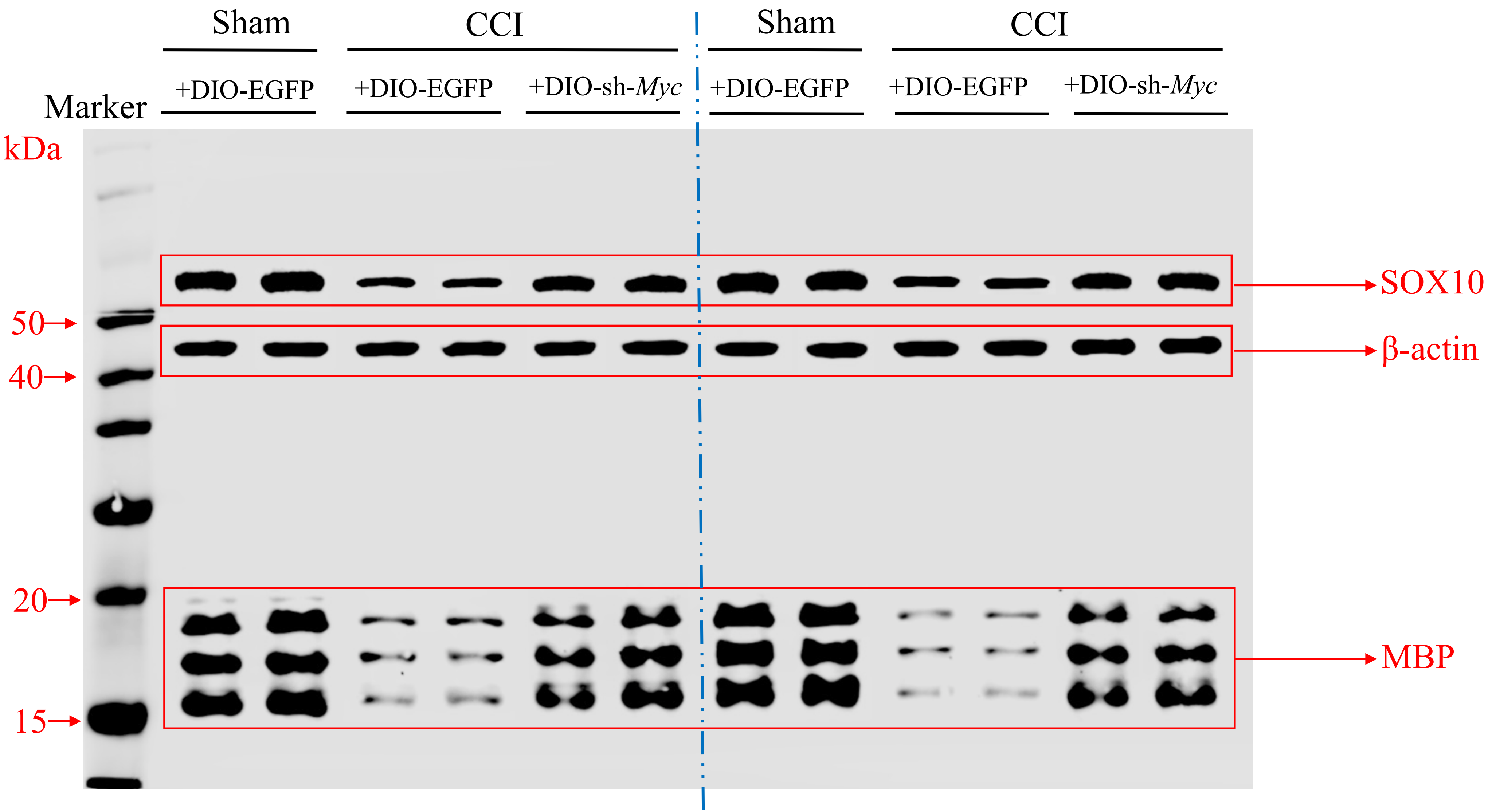

Original Western Blot Images for Figure S3E

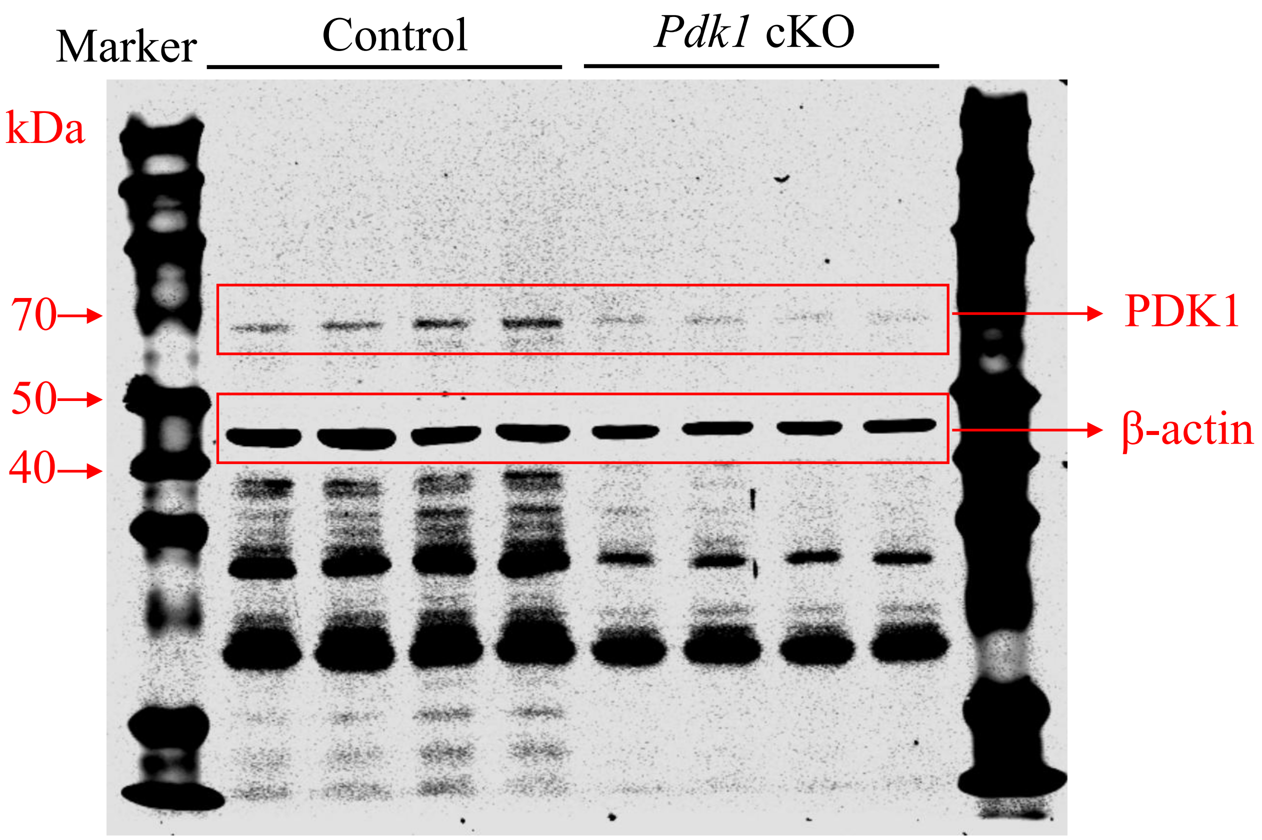

Original Western Blot Images for Figure S4D&S7A

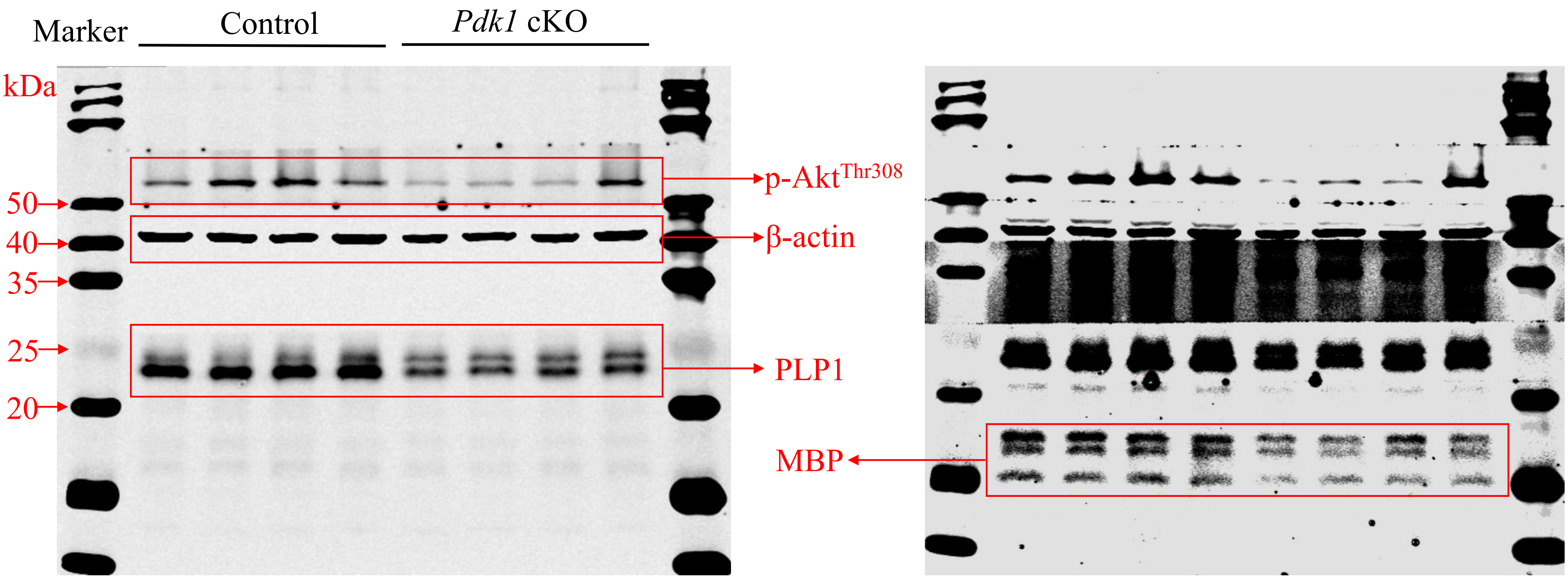

Day1

Day2

Original Western Blot Images for Figure S7A

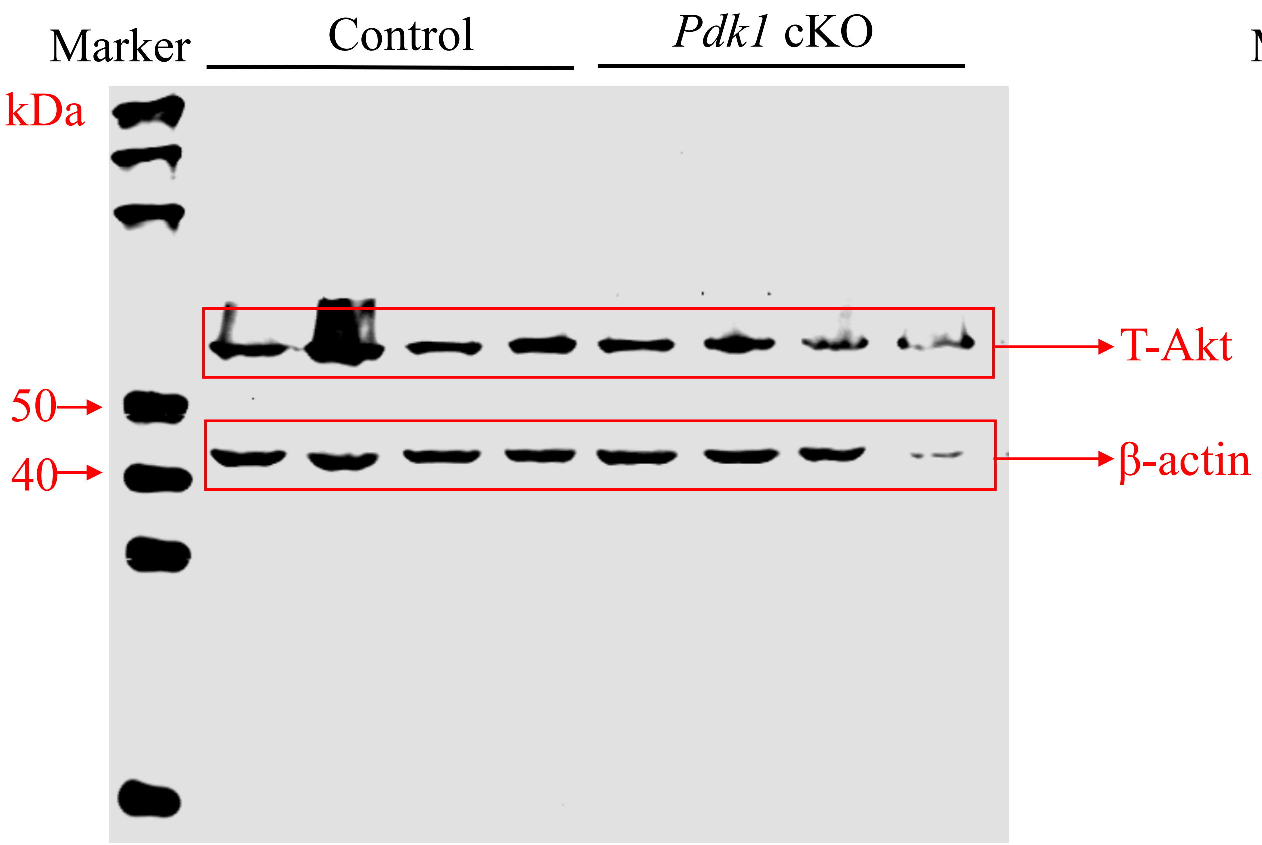

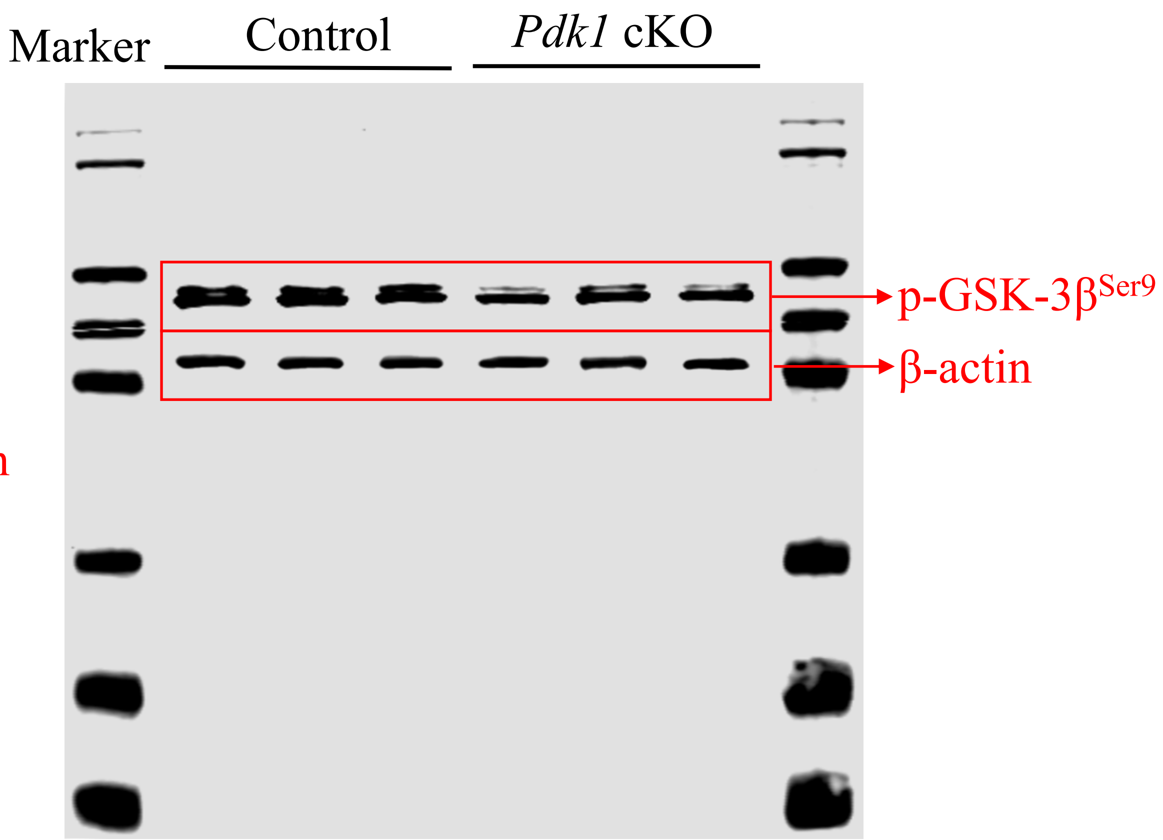

Original Western Blot Images for Figure S7D

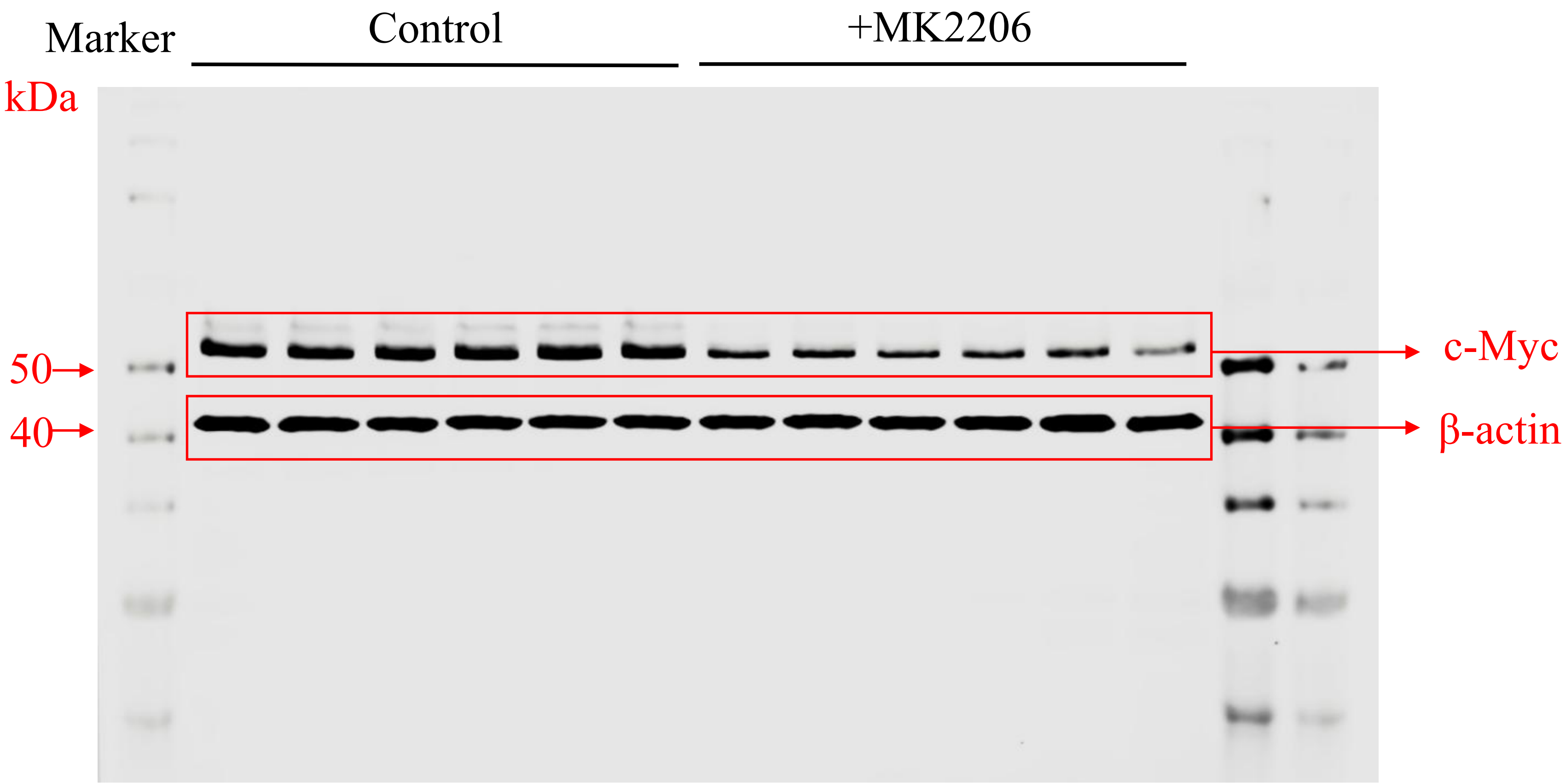

Original Western Blot Images for Figure S7F

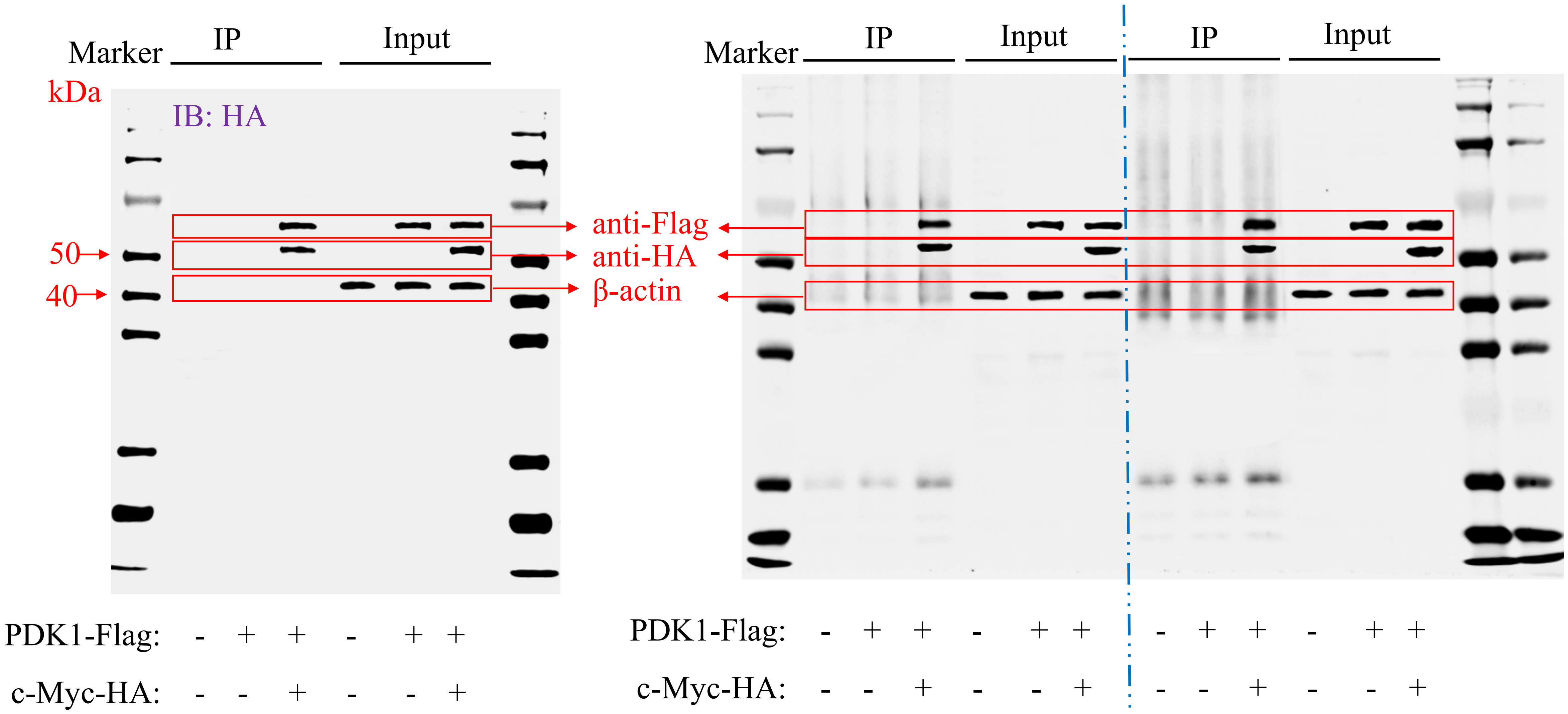

Original Western Blot Images for Figure S7G

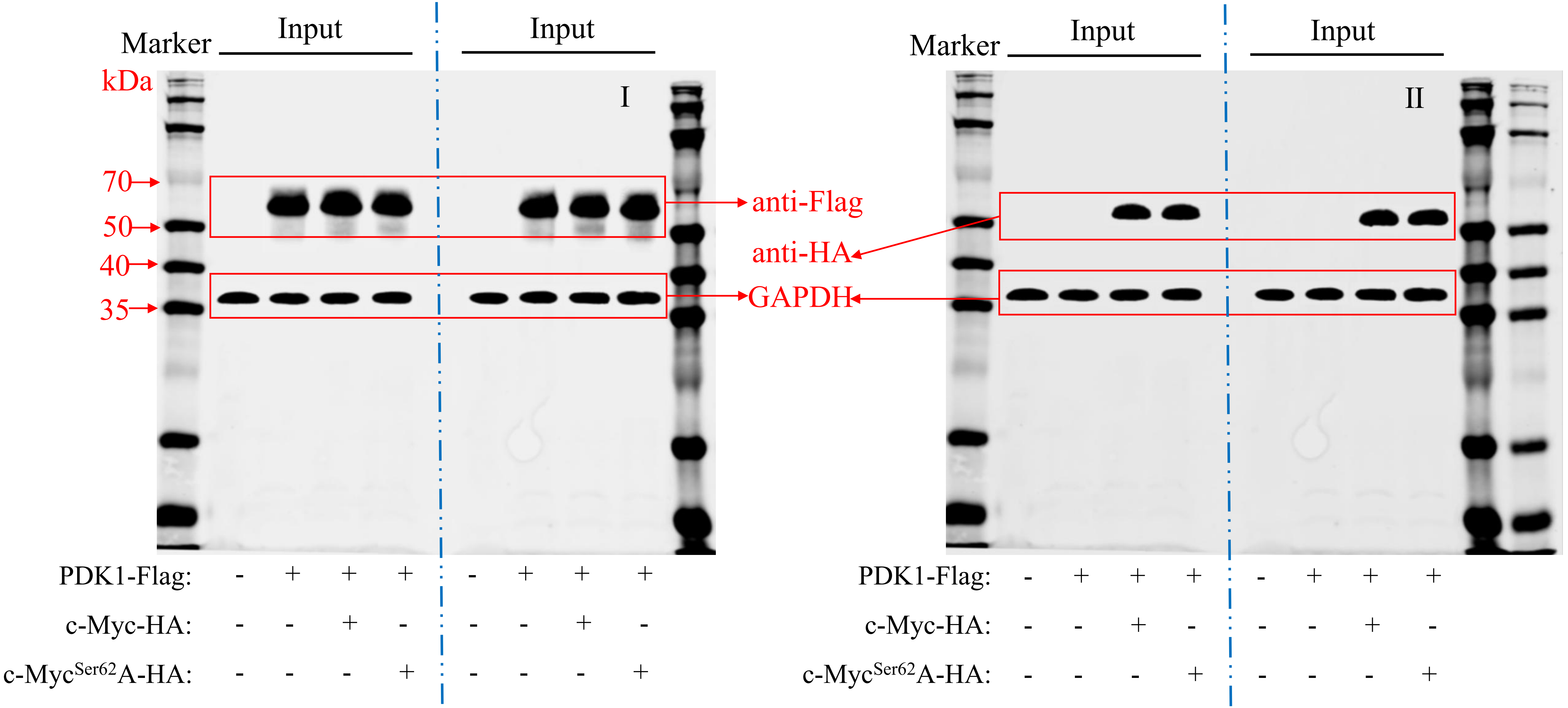

I and II : obtained from the same membrane as in Figure S7G.

Original Western Blot Images for Figure S7G

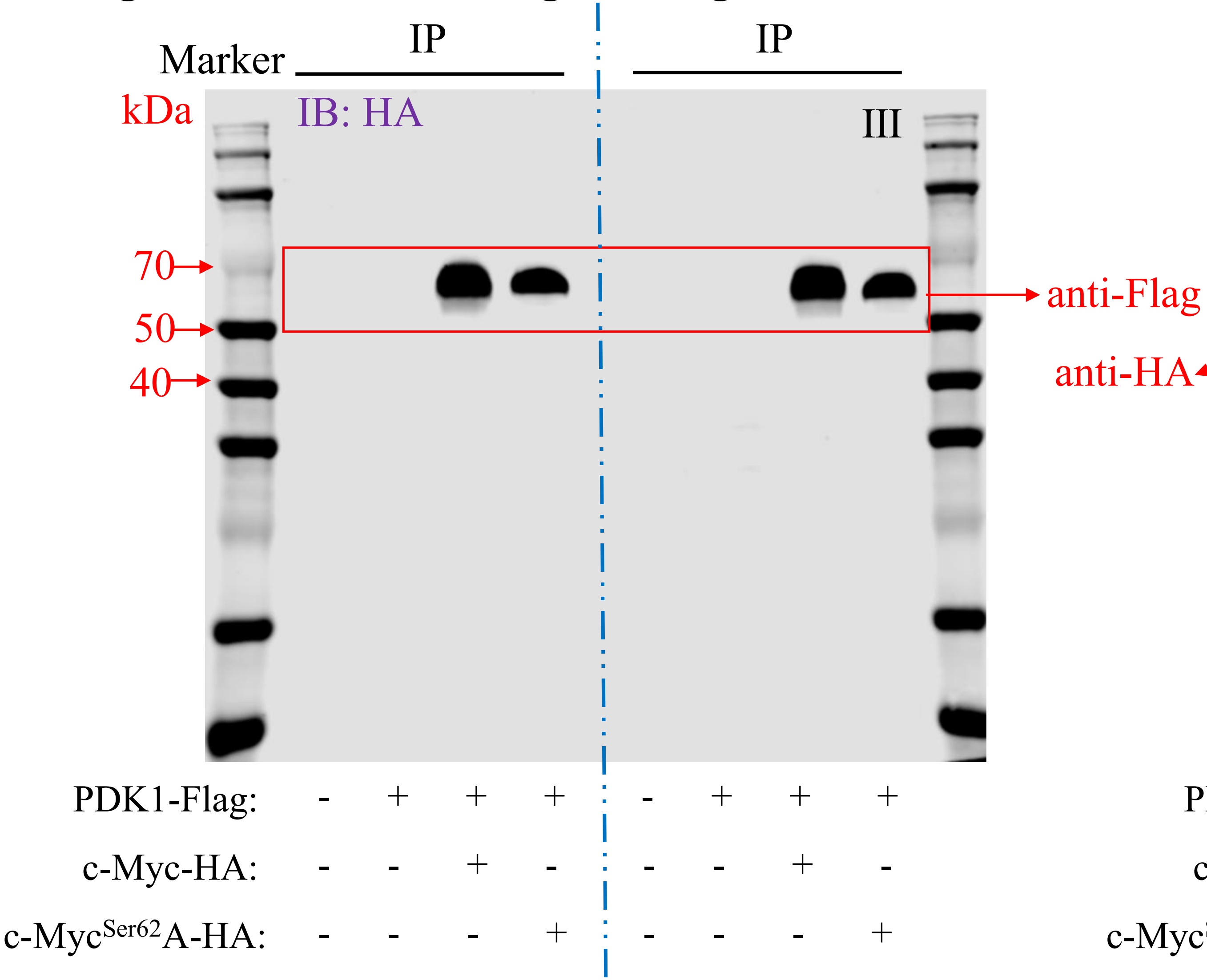

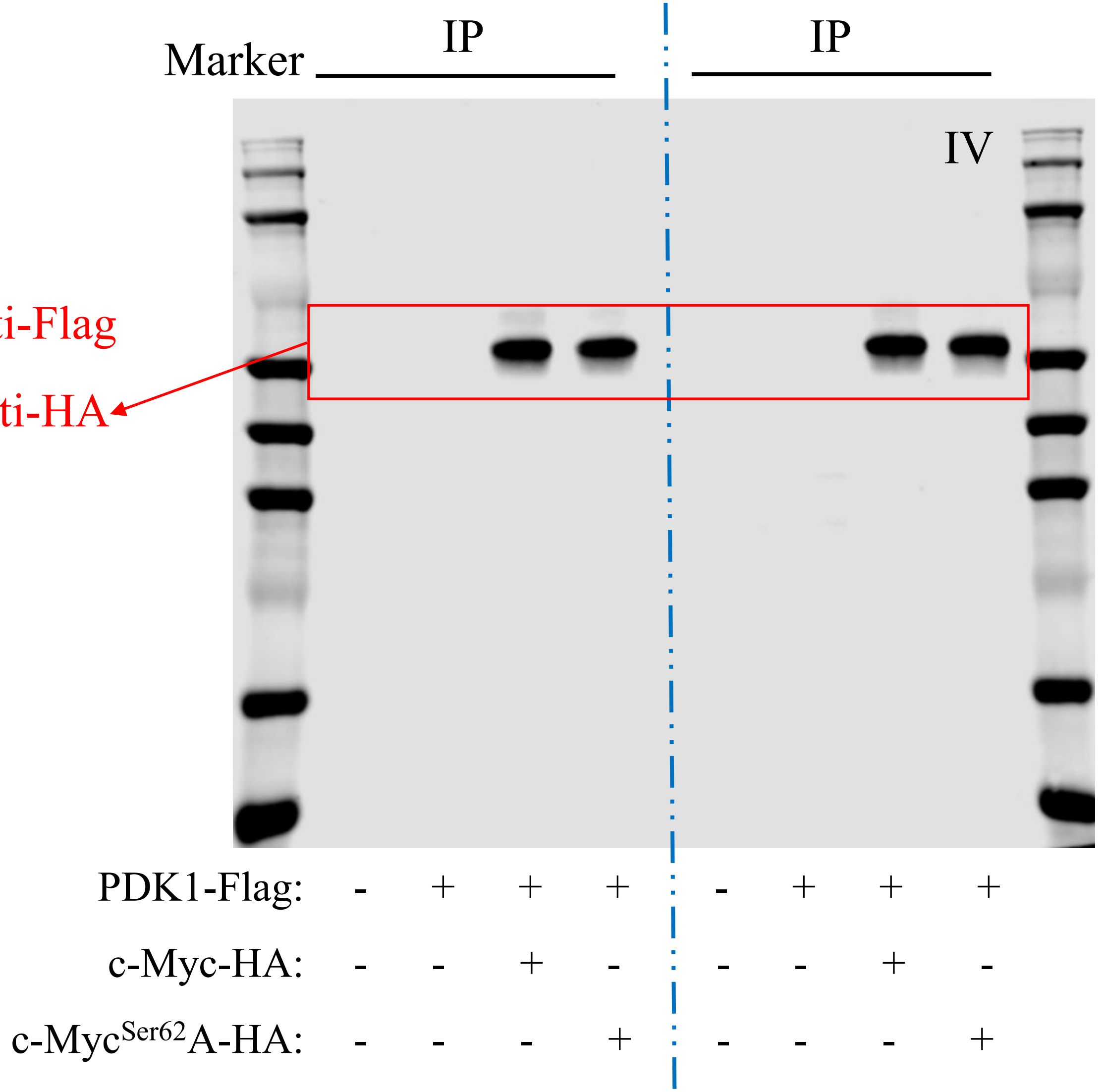

III and IV : obtained from the same membrane as in Figure S7G.

Original Western Blot Images for Figure S7G

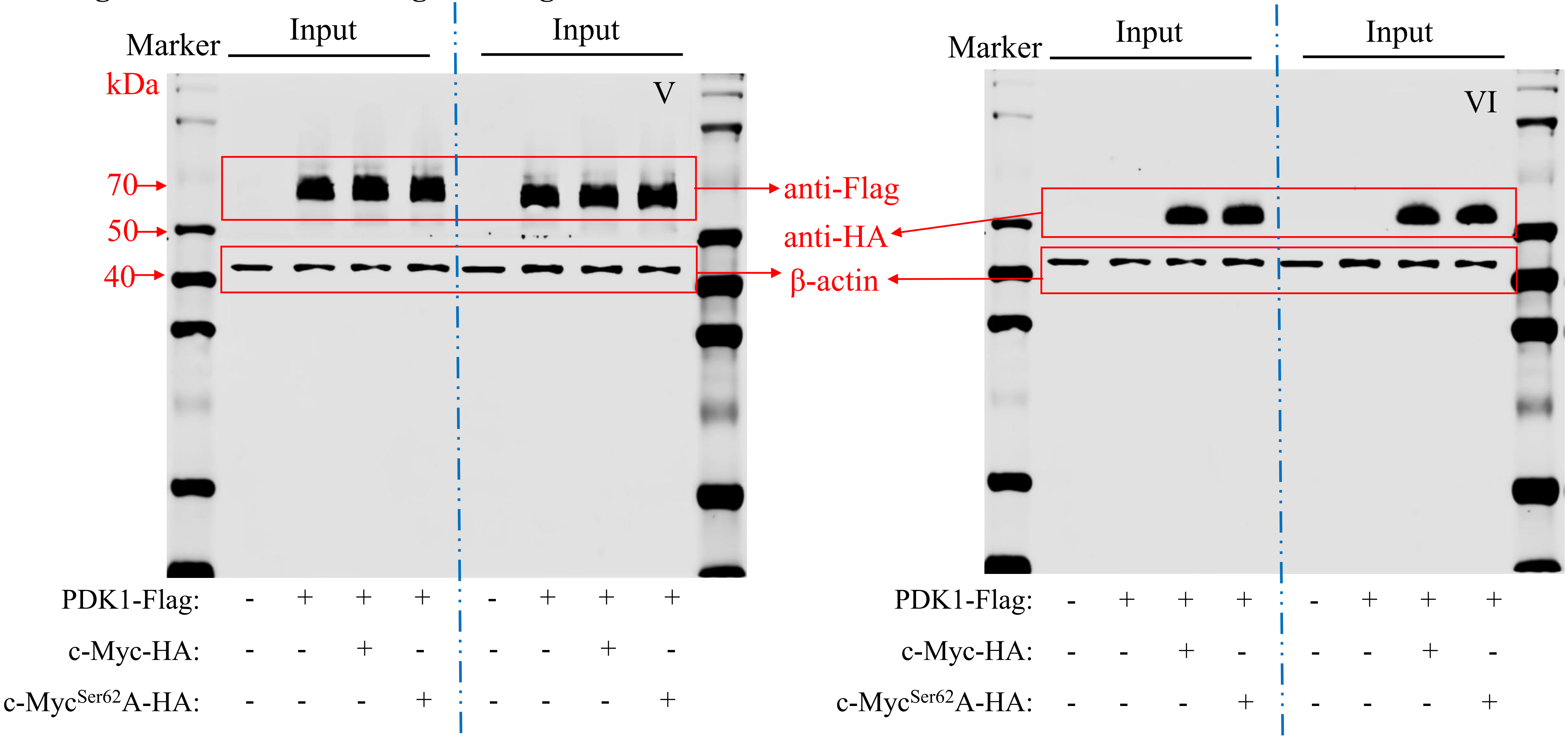

V and VI: obtained from the same membrane as in Figure S7G.

Original Western Blot Images for Figure S7G

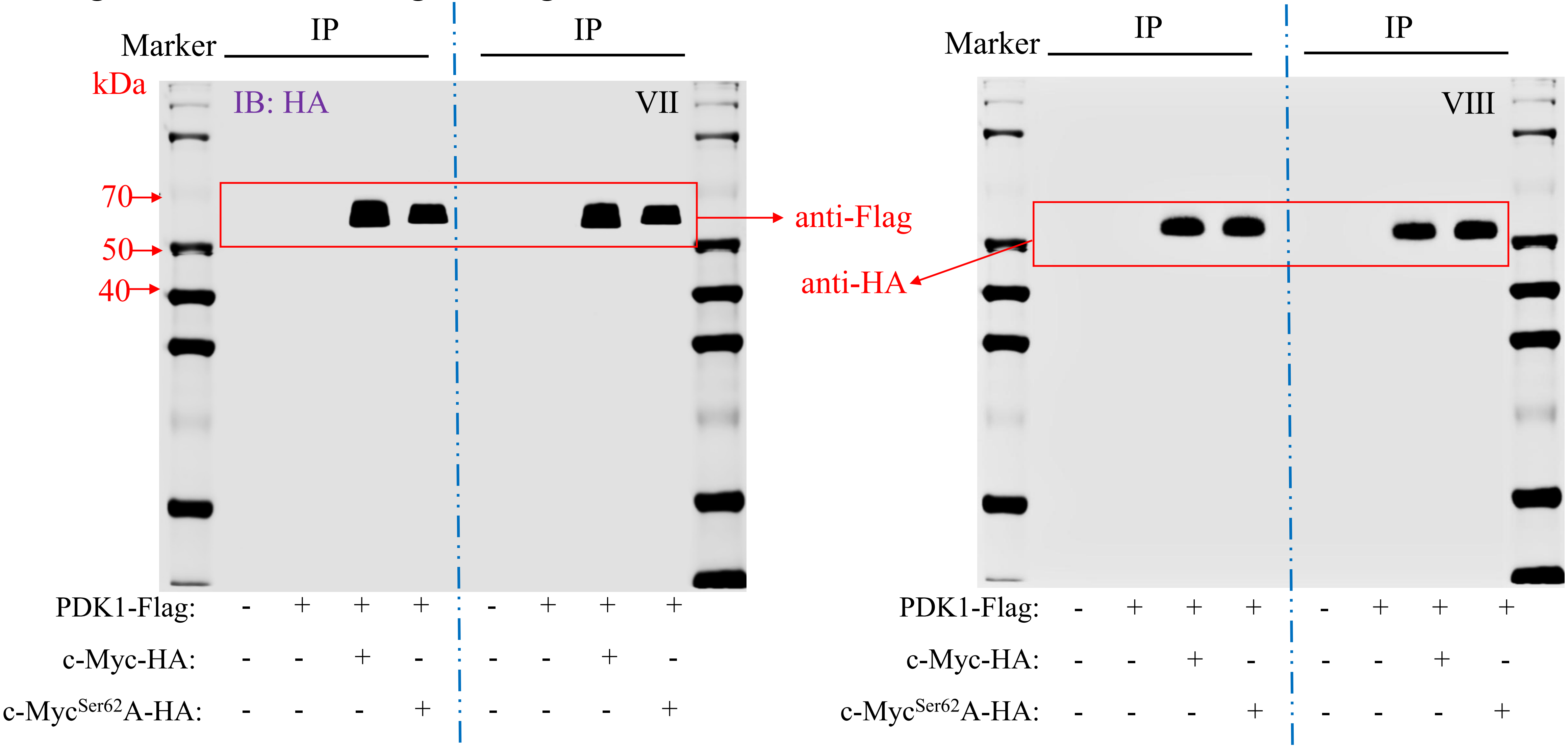

VII and VIII : obtained from the same membrane as in Figure S7G.

Original Western Blot Images for Figure S8A

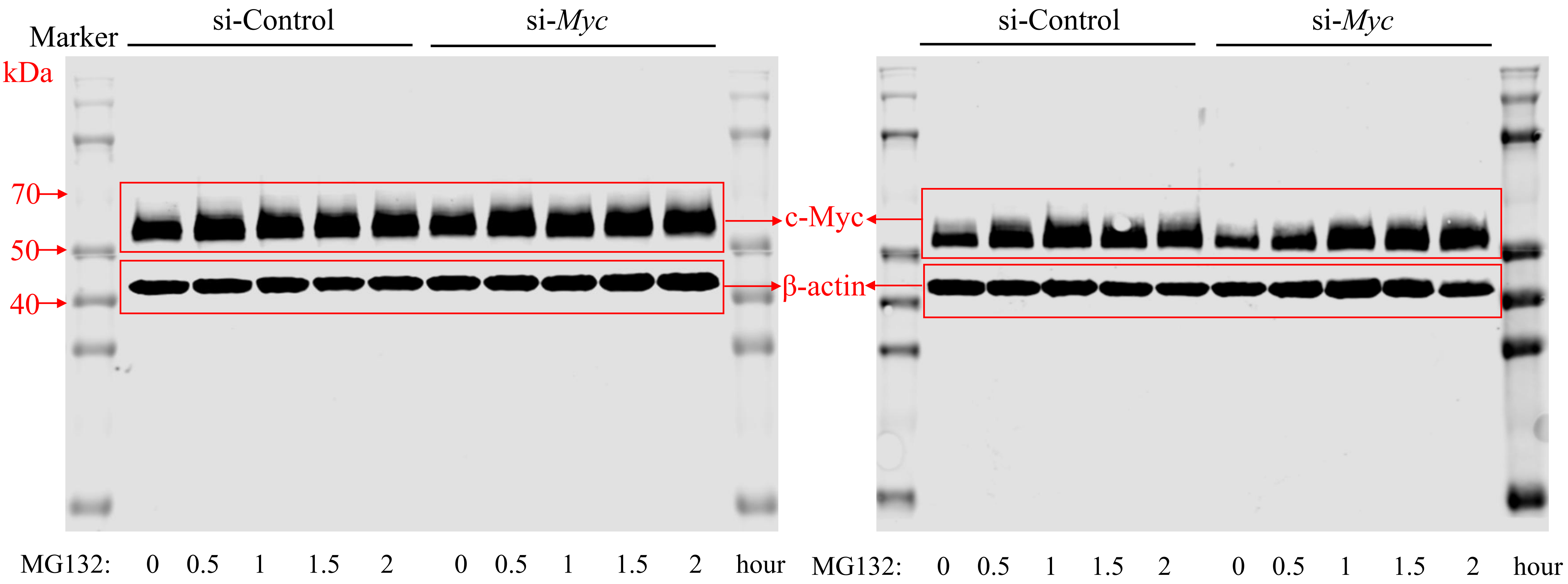

Original Western Blot Images for Figure S8A&S8C

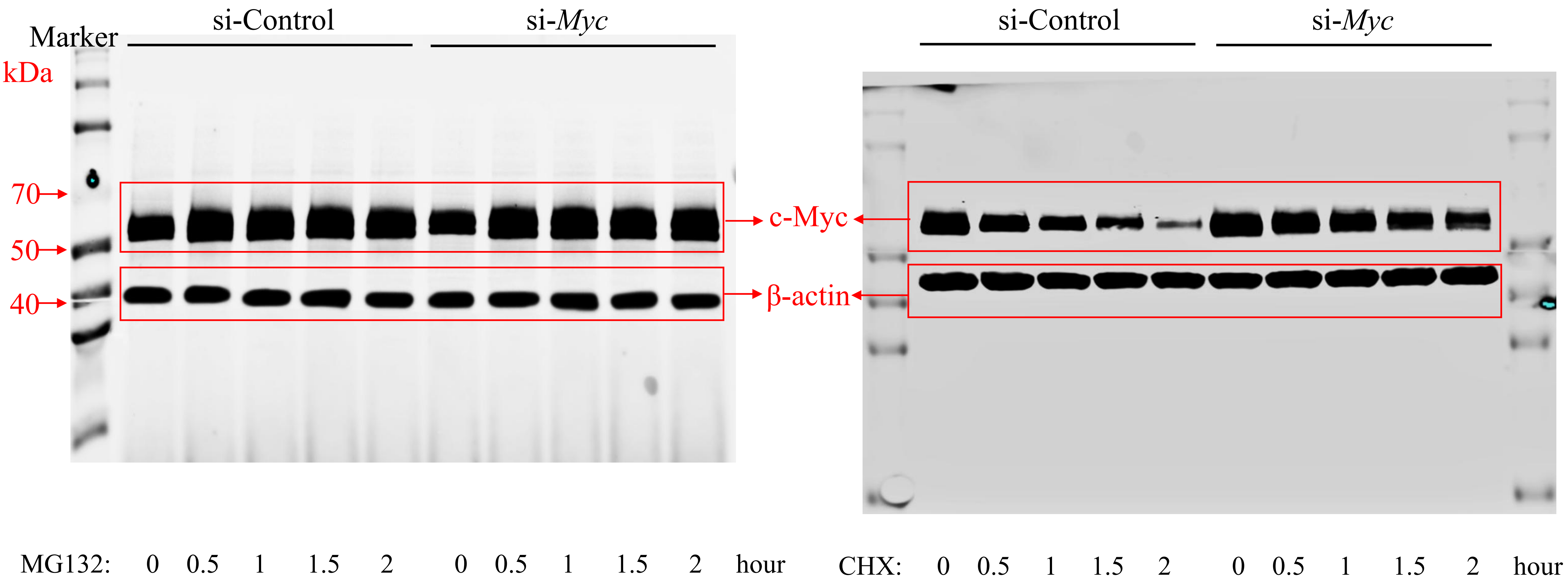

Original Western Blot Images for Figure S8C

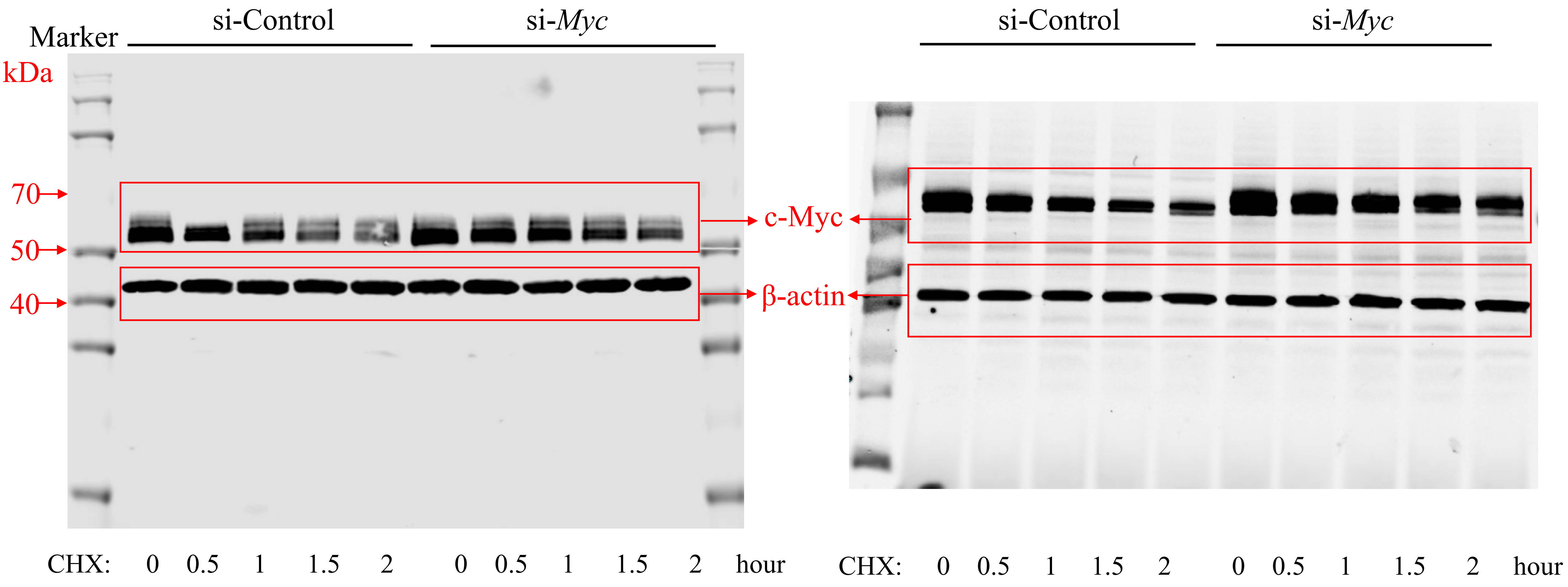

Supplement: Supplementary file 2 — Supporting File 2: advs75281‐sup‐0002‐Data.zip. [file ADVS-13-e16426-s002.zip › Supporting Information 2_All Original Western Blot Images.pdf]
