# Supplementary figures and images for: Targeting the PDK1/c‐Myc/SOX10 Signaling in Oligodendrocytes Alleviates Neuropathic Pain
Source: Adv Sci (Weinh). 2026 Apr 16;13(39):e16426. doi: 10.1002/advs.202516426 (PMC13334879; doi:10.1002/advs.202516426)

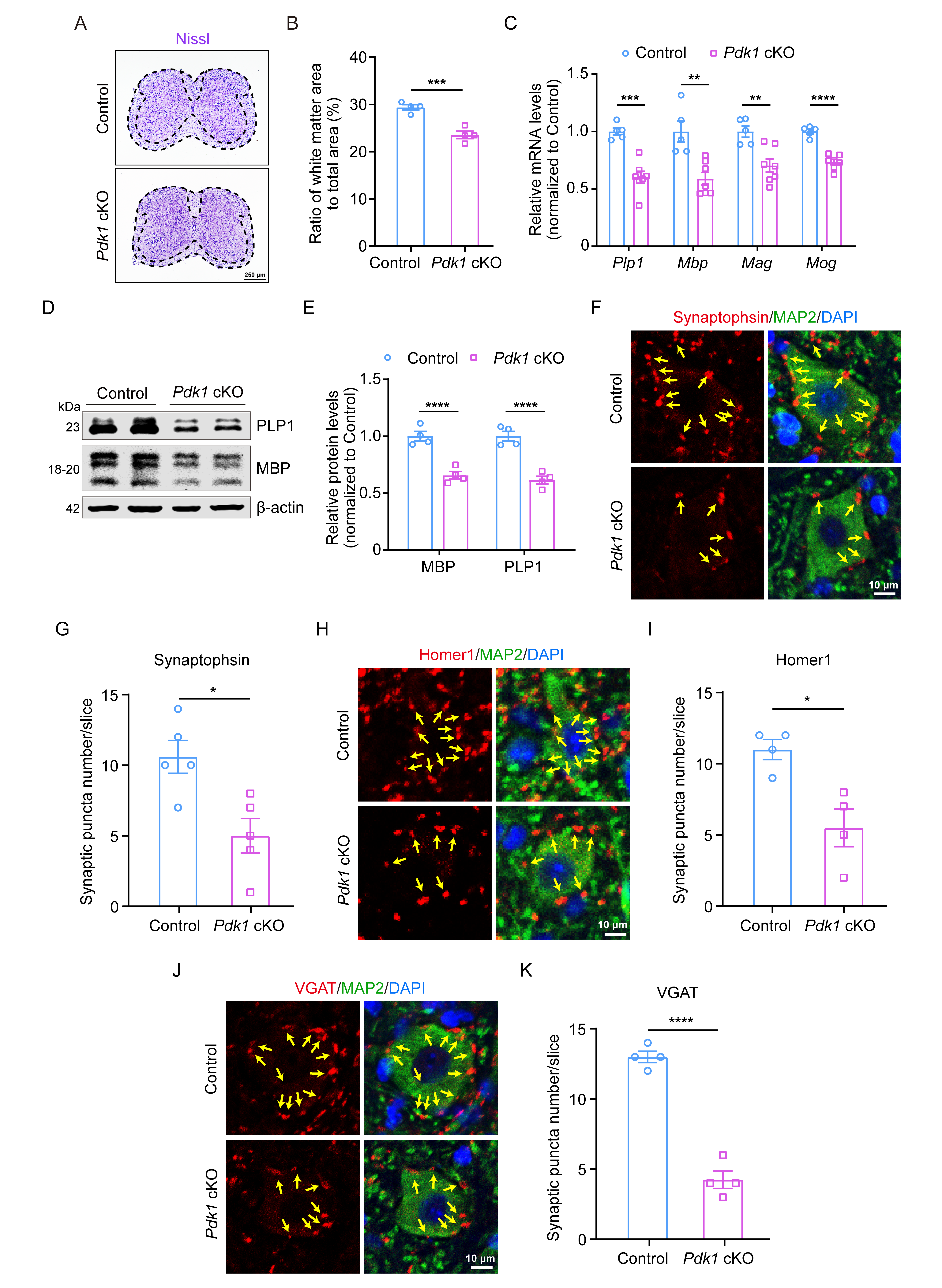

Supplement: Supplementary file 2 — Supporting File 2: advs75281‐sup‐0002‐Data.zip. [file ADVS-13-e16426-s002.zip › Figure.S4.tif]

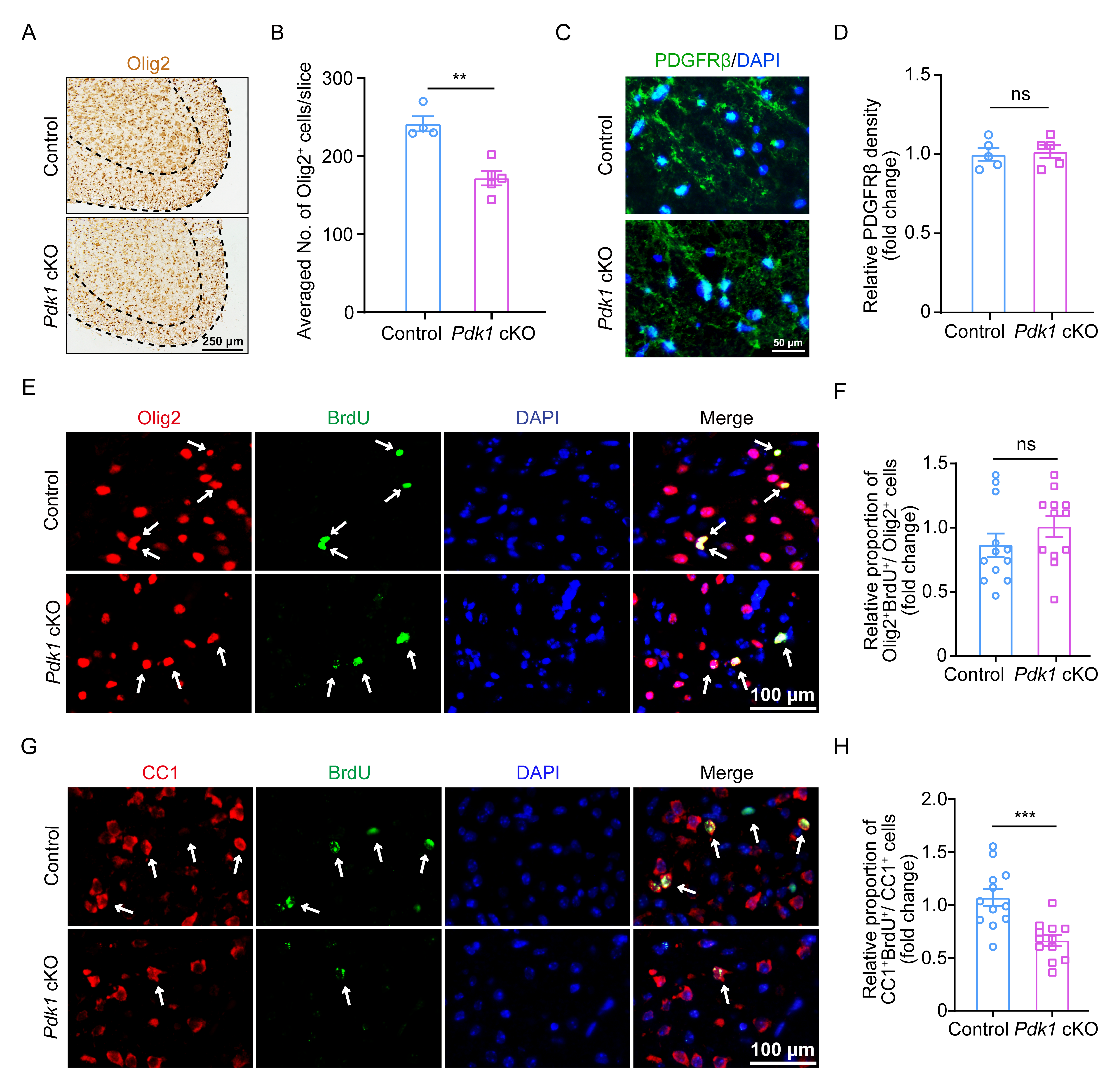

Supplement: Supplementary file 2 — Supporting File 2: advs75281‐sup‐0002‐Data.zip. [file ADVS-13-e16426-s002.zip › Figure.S5.tif]

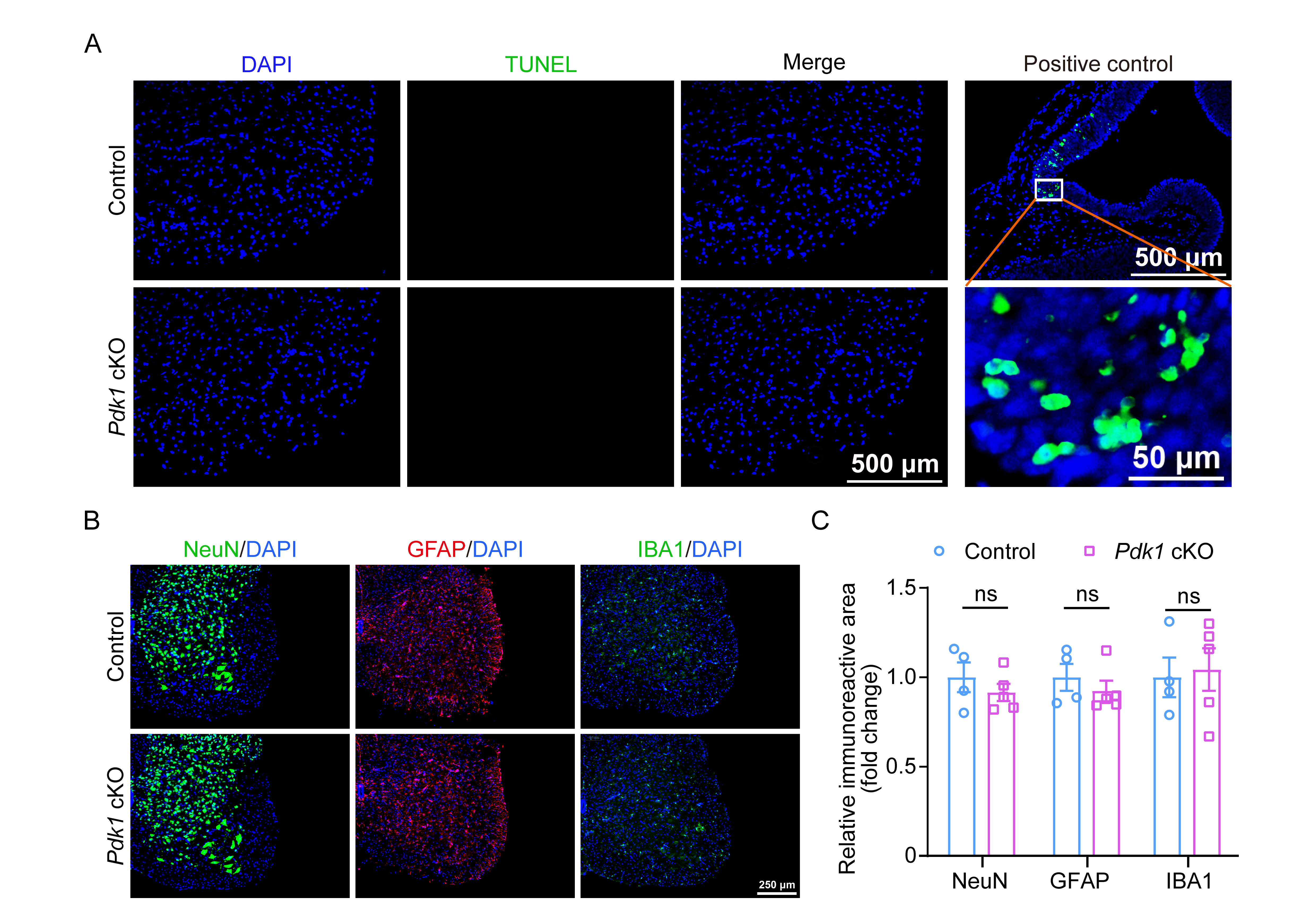

Supplement: Supplementary file 2 — Supporting File 2: advs75281‐sup‐0002‐Data.zip. [file ADVS-13-e16426-s002.zip › Figure.S6.tif]

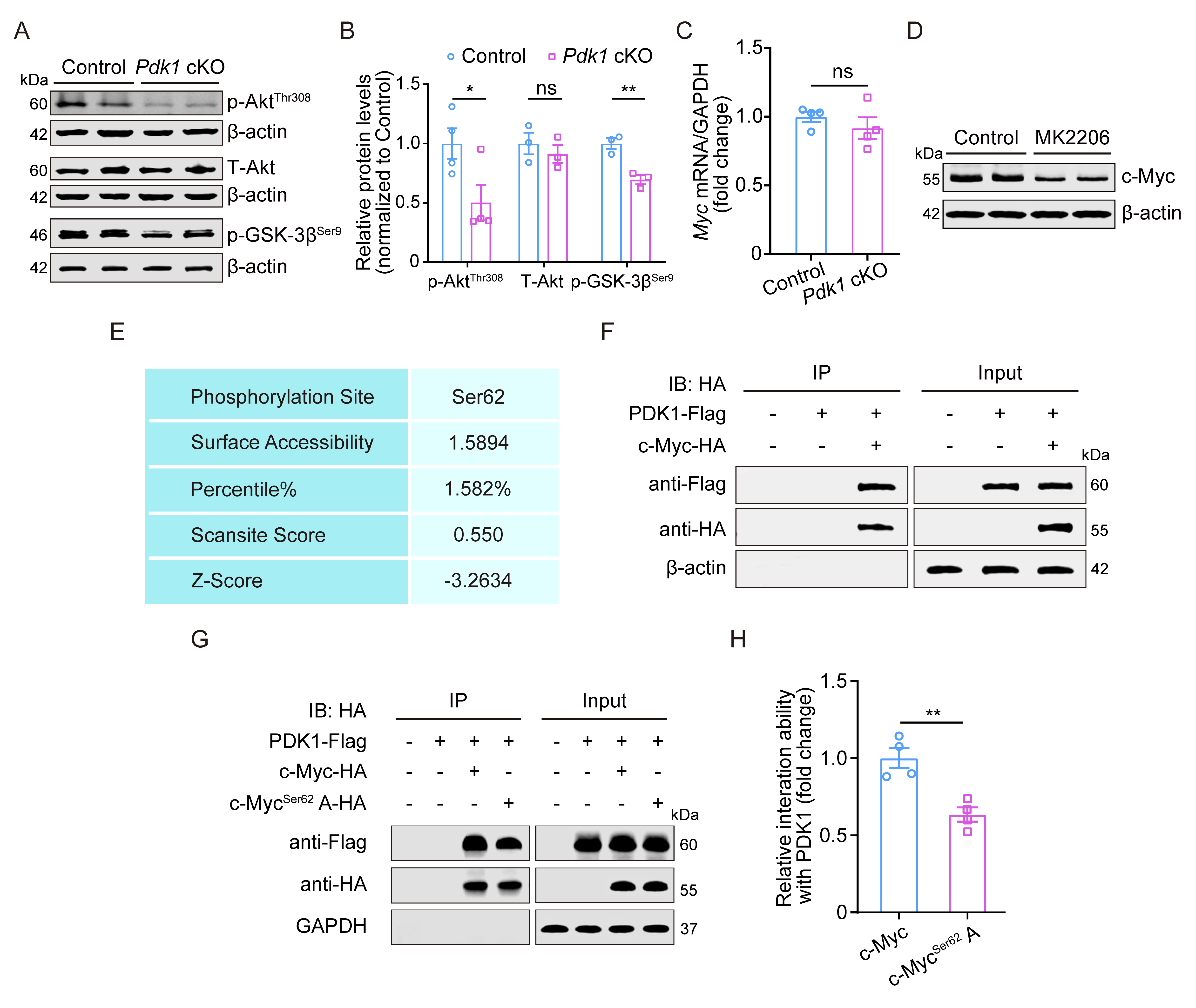

Supplement: Supplementary file 2 — Supporting File 2: advs75281‐sup‐0002‐Data.zip. [file ADVS-13-e16426-s002.zip › Figure.S7.tif]

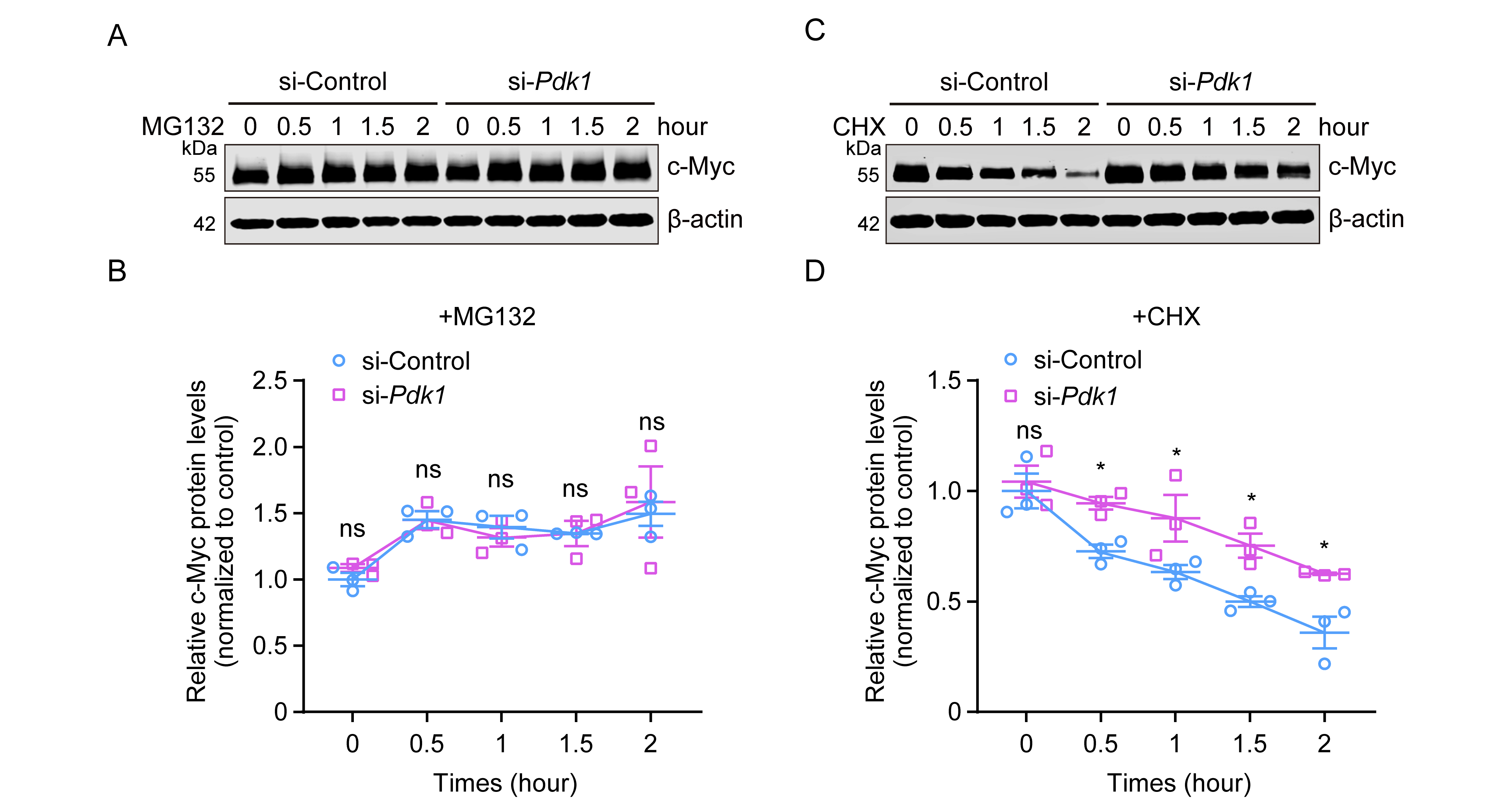

Supplement: Supplementary file 2 — Supporting File 2: advs75281‐sup‐0002‐Data.zip. [file ADVS-13-e16426-s002.zip › Figure.S8.tif]

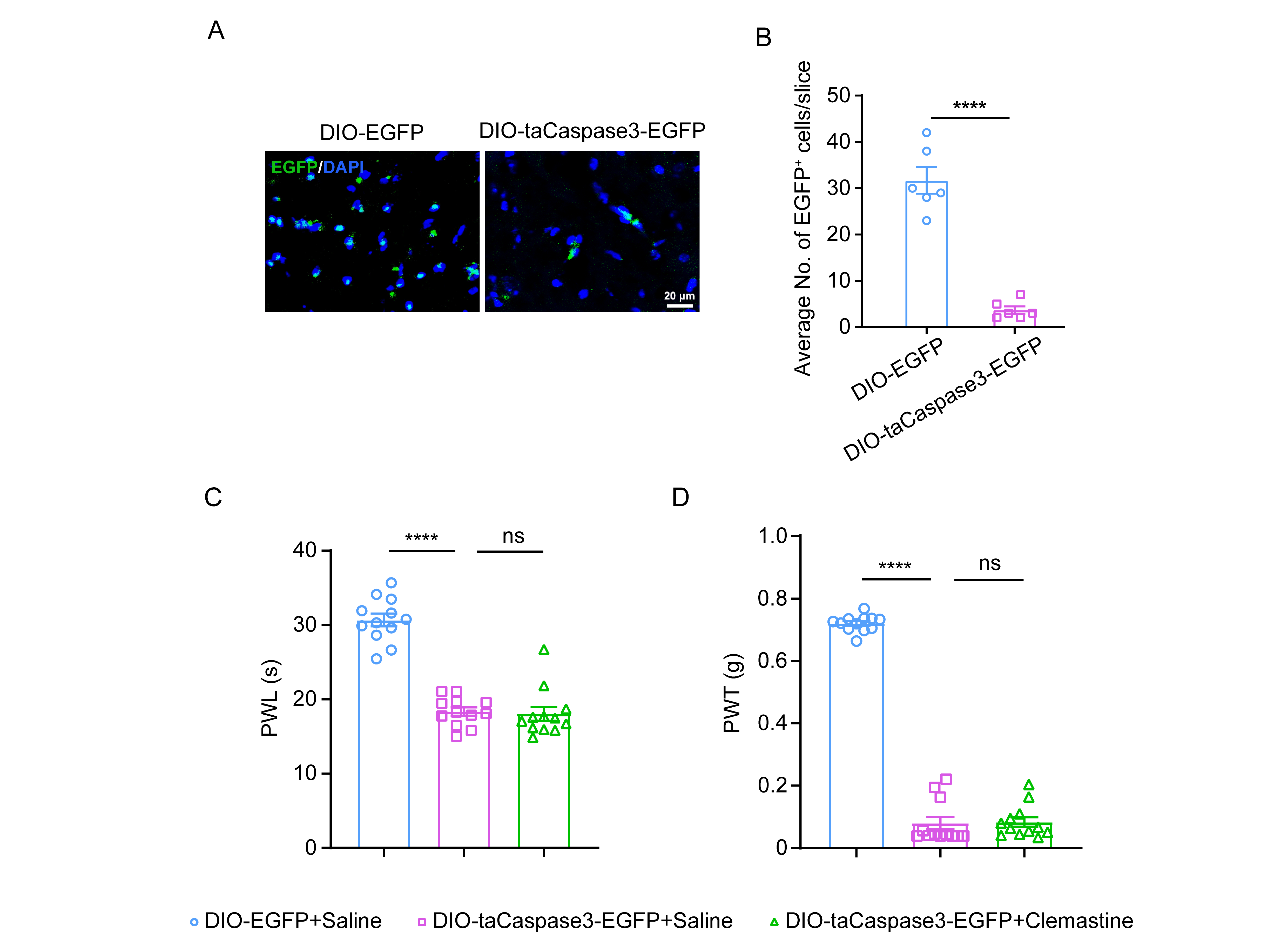

Supplement: Supplementary file 2 — Supporting File 2: advs75281‐sup‐0002‐Data.zip. [file ADVS-13-e16426-s002.zip › Figure.S9.tif]

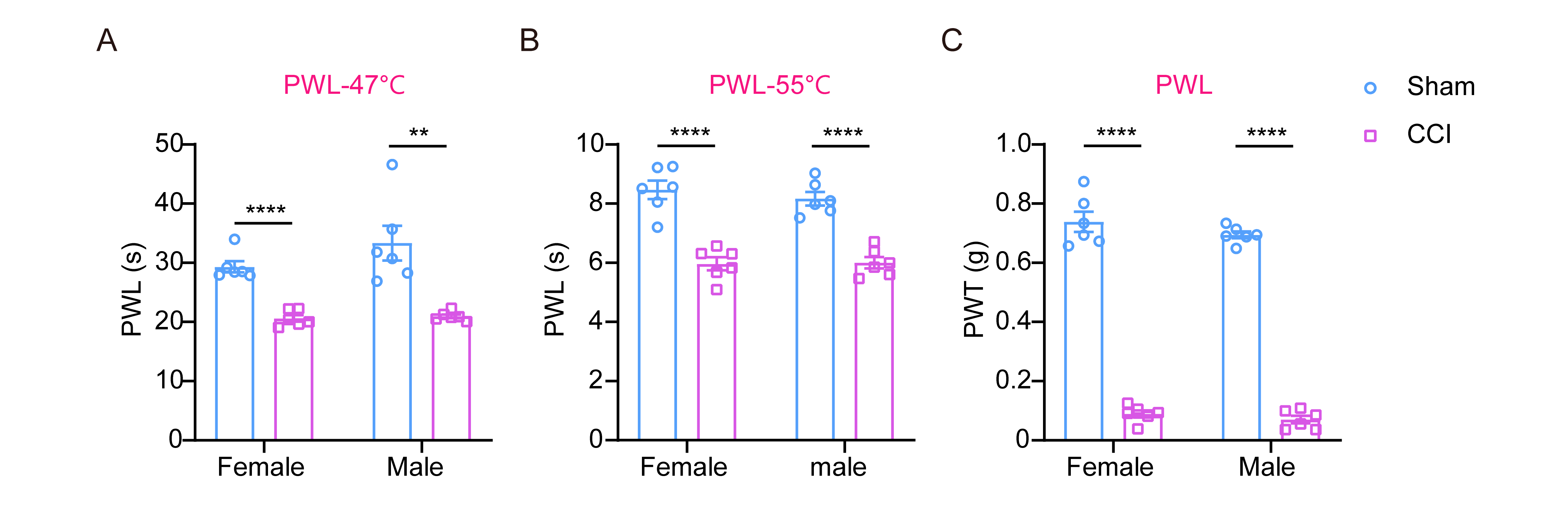

Supplement: Supplementary file 2 — Supporting File 2: advs75281‐sup‐0002‐Data.zip. [file ADVS-13-e16426-s002.zip › Figure.S1.tif]

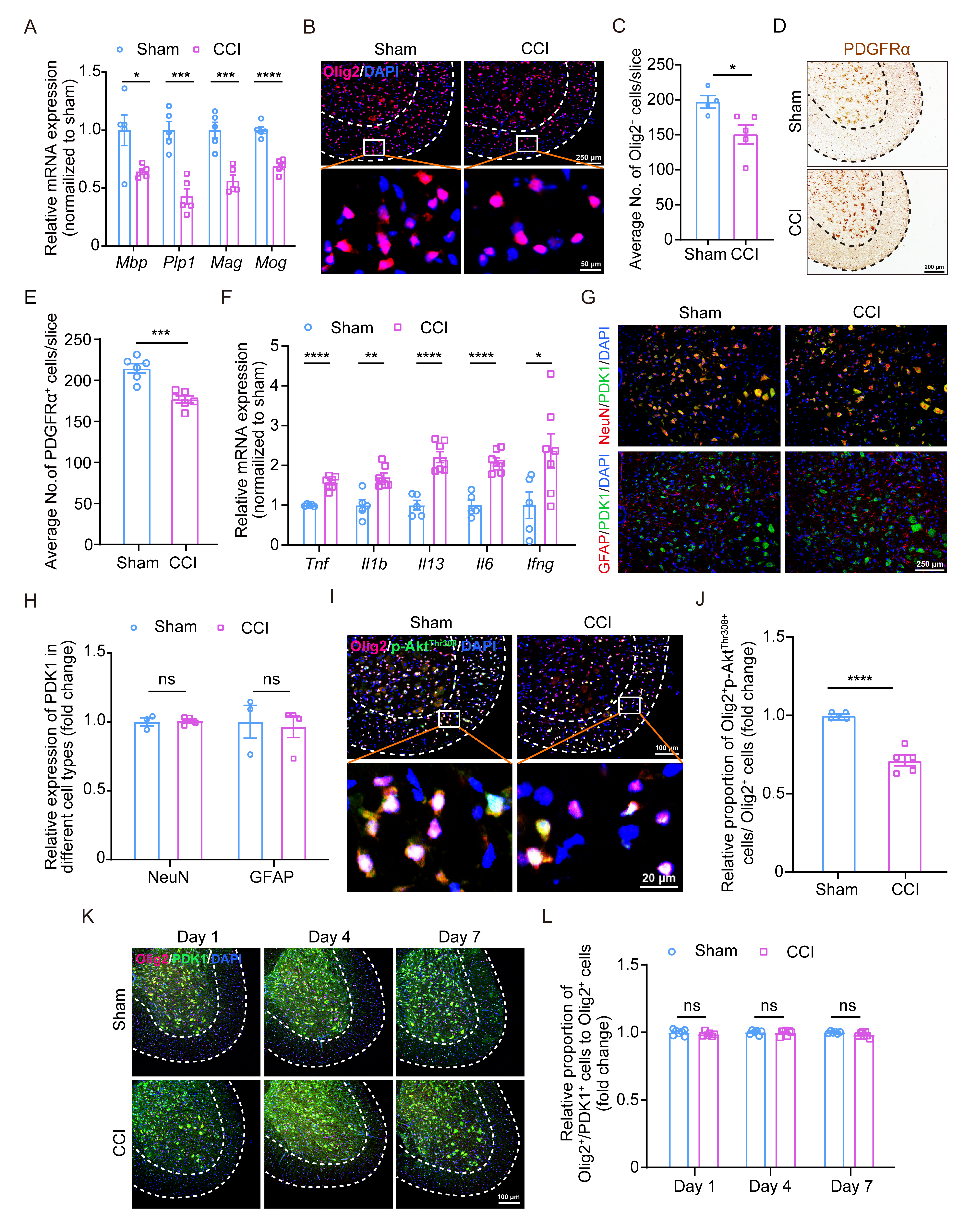

Supplement: Supplementary file 2 — Supporting File 2: advs75281‐sup‐0002‐Data.zip. [file ADVS-13-e16426-s002.zip › Figure.S2.tif]

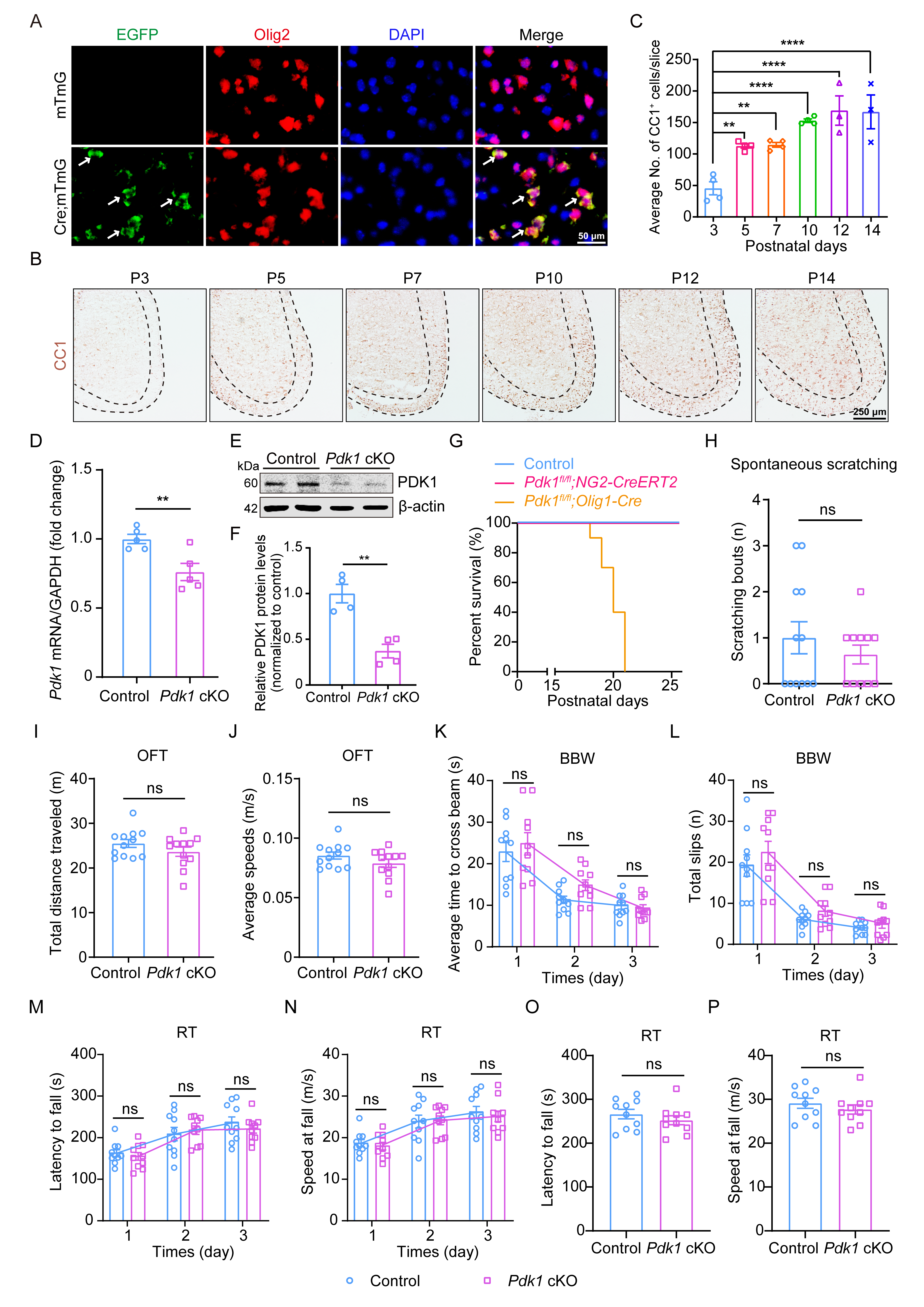

Supplement: Supplementary file 2 — Supporting File 2: advs75281‐sup‐0002‐Data.zip. [file ADVS-13-e16426-s002.zip › Figure.S3.tif]
